# Application of culturomics in fungal isolation from mangrove sediments

**DOI:** 10.1186/s40168-023-01708-6

**Published:** 2023-12-11

**Authors:** Meng Li, Mubashar Raza, Shuang Song, Lingwei Hou, Zhi-Feng Zhang, Min Gao, Jun-En Huang, Fang Liu, Lei Cai

**Affiliations:** 1 State Key Laboratory of Mycology, Institute of Microbiology, Chinese Academy of Sciences, Beijing, 100101 China; 2 College of Life Sciences, University of Chinese Academy of Sciences, Beijing, 100049 China; 3 Key Laboratory of Integrated Pest Management On Crops in Northwestern Oasis, Ministry of Agriculture and Rural Affairs, Institute of Plant Protection, Xinjiang Academy of Agricultural Sciences, Urumqi, 830091 China; 4 Key Lab of Space Nutrition and Food Engineering, China Astronaut Research and Training Center, Beijing, 100094 China; 5 Southern Marine Science and Engineering Guangdong Laboratory (Guangzhou), Guangzhou, 511458 China

**Keywords:** Enrichment cultivation, In situ cultivation, Mangrove sediments, Rare dark matter fungi, Novel taxa

## Abstract

**Background:**

Fungi play a crucial role in ecosystems, and they have been widely considered a promising source for natural compounds that are crucial for drug discovery. Fungi have a high diversity, but about 95% of them remain unknown to science. The description rate of fungi is very low, mainly due to the inability of most fungi to grow in artificial media, which could not provide a sufficiently similar environment to their natural habitats. Moreover, many species in nature are in a state of low metabolic activity which cannot readily proliferate without proper resuscitation. Previously developed culturomics techniques are mostly designed and applicable for bacteria, with few attempts for fungal isolation because of their significantly larger cell size and hyphal growth properties.

**Results:**

This study attempted to isolate previously uncultured and rare fungi from mangrove sediments using newly developed fungal enrichment culture method (FECM) and fungal isolation chips (FiChips). Comparison of fungal community composition at different enrichment stages showed that FECM had great influence on fungal community composition, with rare taxa increased significantly, thus improving the isolation efficiency of previously uncultured fungi. Similarly, in situ cultivation using FiChips has a significant advantage in detecting and culturing rare fungi, as compared to the conventional dilution plate method (DPM). In addition, based on morphological comparisons and phylogenetic analyses, we described and proposed 38 new ascomycetous taxa, including three new families, eight new genera, 25 new species, and two new combinations (presented in additional file 1).

**Conclusions:**

Our study demonstrated that mangrove sediments harbor a high diversity of fungi, and our new isolation approaches (FECM and FiChips) presented a high efficiency in isolating hitherto uncultured fungi, which is potentially usable for fungal isolation in other similar environments﻿.

Video Abstract

**Supplementary Information:**

The online version contains supplementary material available at 10.1186/s40168-023-01708-6.

## Background

The fungal kingdom is one of the most diverse and oldest clades of the eukaryotic life, with an estimated 2.2–3.8 million species that play important roles in the terrestrial and aquatic ecosystems [1, 2]. However, fungi have the lowest rate of description among the major eukaryotic groups [3]. The proportion of fungal species that have been described is 3–6%, compared to 83–96% and 19% for plants and animals, respectively [3]. With the rapid development of high-throughput sequencing (HTS), increasing studies have indicated that the diversity of fungi in the natural environments may be even higher than previously estimated [4, 5]. Traditional isolation protocols, such as dilution plate method (DPM), have been widely used for decades but are increasingly recognized as a bottleneck hindering the understanding of true fungal diversity, because to date only a small fraction of fungi were successfully cultured [6, 7]. Exploring new techniques for fungal isolation is of great importance for revealing fungal diversity in various environments and for accessing new biological resources for various application industries.

Most microorganisms in the environment cannot grow under artificial laboratory conditions [8], and they are commonly referred to as the “dark matter” of microorganisms, suggesting that little is known about them, although sure that they exist [9, 10]. Recent studies have shown that some previously uncultured microorganisms may grow in the laboratory if they can be provided nutrients completely similar to those in the natural environment [11]. However, the main obstacle is that the chemical composition and physical parameters required for growth of the various microorganisms are different, and in most cases, they remain unclear, let alone providing them in laboratory cultures. Therefore, a better access to the “dark matter” microorganisms may require design of more innovative isolation protocol different from the traditional methods. Several techniques have recently been developed, such as diffusion chambers [11] and isolation chips (iChips) [12], used in in situ cultivation. In these approaches, microorganisms could absorb nutrients and growth factors from the environments but grow in a restricted chamber. These approaches resulted in a 30,000% increase in microbial recovery compared with traditional methods [12–15]. Nichols et al. found that the species composition obtained through iChips and Petri dishes were significantly different, and the number of novel organisms obtained from iChips substantially exceeded that from Petri dish [12].

In addition, some microorganisms need to grow with interaction with other microorganisms [16]. For these microbes, simply providing natural environmental factors is insufficient to guarantee their growth, for example, previous studies have shown that some isolates can only be grown with mixed cultures [11, 17]. Enrichment cultivation (also called mixed cultivation or co-cultivation) has been first applied for bacterial isolation in 1895 by Sergei Winogradsky [18]. Enrichment cultivation can provide a simple community enabling the communication among microorganisms and has been considered a good strategy to isolate previously uncultured microorganisms [17, 19, 20]. By using the enrichment cultivation, several rare taxa and higher-order taxa have been successfully isolated [21].

Although there have been studies of isolating microorganisms through in situ techniques, they mainly focused on bacteria, with few attempts on fungal isolation [22]. This is mainly because these emerging isolation techniques were designed based on bacterial characteristics and are not suitable for fungi. As an important group of eukaryote, fungi presents in natural environments in complex forms, including sexual spores, asexual spores, mycelia, and even more complex fruit bodies [23]. No matter in what forms, fungal cells are constantly much larger than bacterial cells, not to mention that they are mostly multicellular, even in their simplest forms, i.e., sexual and asexual spores. Therefore, previously designed devices, especially those involving certain filter membrane, mostly do not suit fungi. Furthermore, the growth rate of fungi varies largely [24]; therefore, the enrichment cultivation of fungi requires a consideration of inhibiting fast-growing fungi, thus to obtain those rare ones. The widely used compartmentalizing clonal populations in microtiter plate wells of microfluidics are also unfeasible for fungi, as even using these expensive automated colony pickers and liquid handling robots; it is impossible to manipulate filamentous fungi because they usually form dense mycelia or leathery colonies [25].

The marine environment, including the sediments, is a huge reservoir of low-abundance dormant microorganisms, with the potential to recover with environmental changes [26, 27]. Marine fungi have rarely been studied, with the number of known species increased from 174 to 1692 species over the past 50 years [28], among which a significant proportion was from mangroves. Over the past 25 years, there has been 395 species documented from decomposing mangrove wood and 193 from mangrove sediments [28]. The unique environment of mangrove sediments harbors a large number of unique fungal species [29, 30], and a growing number of studies have suggested that fungi derived from mangrove sediments not only exert important ecological functions but also have great potential in biotechnological application [31, 32].

Overall, investigations of fungal diversity in mangrove sediments are very limited, particularly in attempts to isolate hitherto uncultured species. This study aims to apply our self-designed FECM and FiChips protocols to fungal isolation from mangrove sediments, and to evaluate the isolation efficiencies of different isolation protocols by comparing the species composition revealed by different protocols and high-throughput sequencing. Furthermore, we performed morphological observations and phylogenetic analyses of the strains isolated only from FECM and FiChips and described 38 new taxa of ascomycetes, including three new families, eight new genera, 25 new species, and two new combinations.

## Results

### Enrichment cultivation improves the efficiency of fungal isolation

**Fig. 1:**
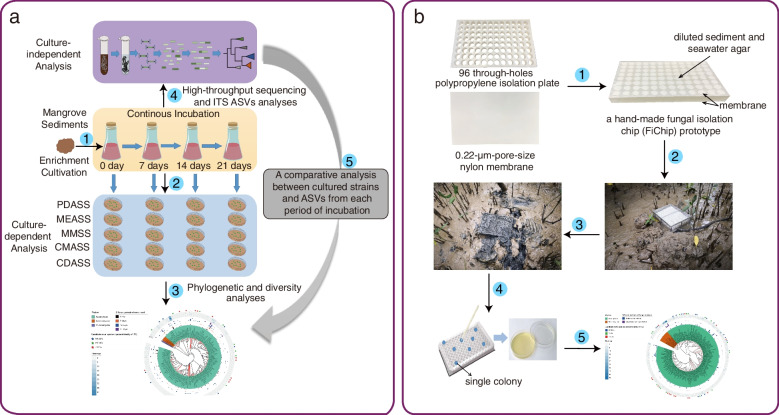
A workflow for enrichment cultivation and in situ incubation of mangrove sediments. **a** Schematic diagram of the fungal enrichment cultivation method (FECM). The process incorporates the following steps: continuous incubation (0–21 days), culture-dependent research, and culture-independent research. **b** Handmade fungal isolation chips (FiChip) prototype for in situ incubation. The process consists of the following steps: FiChips assembly, in situ incubation, fungal isolation, and purification. A detailed protocol is described in Additional file 3

**Fig. 2:**
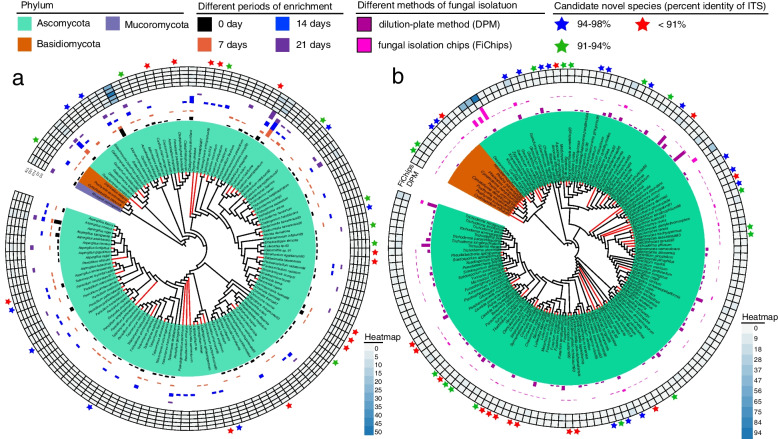
Phylogenetic tree of species isolated through FECM and FiChips, based on full-length ITS sequences. **a** Phylogenetic tree of fungi cultured from the ZJ-1, ZJ-2, and ZJ-3 mangrove sediment samples and correlation analysis of cultured fungi during FECM. **b** Phylogenetic tree of fungi cultured from the SZ-1 and SZ-2 mangrove sediment samples and correlation analysis of cultured fungi by in situ cultivation. Major phylum names are indicated and marked in different colors. Red branches on the trees indicate that the species was isolated after FECM or from FiChips, while black tree branches indicate that the species was isolated by directly culturing on five media. Candidate novel species (blue stars indicate ITS sequence similarity between 94 and 98% comparing to the NCBI database, green stars indicate similarity between 91 and 94%, and red stars indicate similarity below 91%) are indicated by colored stars outside the heatmap. The bar chart and the heatmap indicate the strains number of that species. In addition, detailed information of each species is shown in Additional file 2: Table S1, Table S2, Table S3, and Table S4

**Fig. 3:**
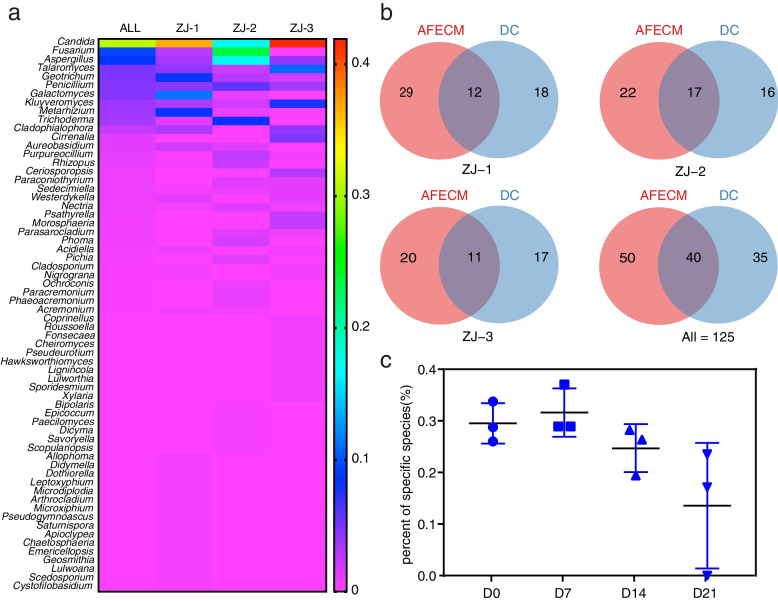
Comparative analyses among culturable fungi during FECM of three sediment samples. **a** The heatmap for generic diversity from three sediment samples. The label ALL indicates the total generic diversity of the isolates. **b** Venn diagram of species between direct cultivation (DC) and after FECM (AFECM). The label DC indicates day 0 of FECM. **c** Percent of specific species during each stage of FECM from three sediment samples (*n* = 3, error bars are s.e.m.). D0–D21 indicate 0, 7, 14, and 21 days of FECM, respectively

Three mangrove sediment samples (ZJ-1, ZJ-2, and ZJ-3) were used for continuously enrichment in shake flasks for 21 days (Fig. 1a). A total of 660 fungal strains were isolated from the enriched suspensions that were collected at day 0, 7, 14, and 21. Preliminary taxonomic assignments based on ITS sequence similarities indicated that these isolates belonged to 125 species in 3 phyla, 9 classes, 23 orders, 41 families, and 64 genera (Fig. 2a, Additional file 2: Table S1), including 29 potential novel species, of which 8 have ITS sequence similarity between 94 and 98% compared to the closest hits in NCBI database, 8 with similarity between 91 and 94%, and 13 with similarity < 91%. The preliminary identification and ITS sequences of each species are listed in Additional file 2: Table S1. Among all isolates, the most dominant fungal phylum was Ascomycota (648 isolates, representing 121 species) with species belonging to four main classes: Saccharomycetes (297 strains, 12 species), Sordariomycetes (164 strains, 43 species), Eurotiomycetes (141 strains, 42 species), and Dothideomycetes (43 strains, 21 species), followed by Basidiomycota and Mucoromycota with 12 isolates (1.8%) represented by Agaricomycetes (2 species), Tremellomycetes (1 species), and Mucoromycetes (1 species) (Fig. 2a, Additional file 2: Table S1). At the generic level, the most abundant (with isolation frequency > 5%) genera in descending order are *Candida*, *Fusarium*, *Aspergillus*, *Talaromyces*, *Geotrichum*, *Penicillium*, and *Galactomyces* (Fig. 3a). These genera have been frequently isolated in previous studies and are considered as common genera in mangrove sediments [30, 33].

Of the 125 species, 35 were isolated only from the original sample (day 0 of FECM), while 50 were isolated only after FECM (AFECM, Figs. 2a and 3b, Additional file 2: Table S2), and other 40 species could be isolated at all periods of FECM. Additionally, some specific species can only be isolated at certain stage of enrichment cultivation (Fig. 2a, Additional file 3: Figure S1). The number and compositions of specific species varies at different periods of FECM. The percentage of specific species increased from day 0 to day 7 but declined thereafter, and nearly one-fold more different species could be obtained from the same sediment samples after FECM (days 7, 14, and 21 of FECM) compared with direct cultivation (DC, day 0 of FECM) (Fig. 3c).

Enrichment cultivation exhibited similar characteristics among different samples: (a) *Candida tropicalis* is the most abundant species in all samples at various enrichment stages (Fig. 2a, Additional file 3: Figure S1). (b) Some potential novel species, some even representing higher rank novel taxa, could be isolated only from enrichment cultivation (Fig. 2a). These results suggested that FECM does improve the efficiency of isolating previously uncultured fungi that could not be isolated from direct dilution plate.

### FECM significantly enriched the rare taxa in the community

To explore the changes of fungal composition during the FECM, the ITS2 locus was amplified and sequenced using the samples collected every 7 days. After quality trimming, a total of 4,356,628 reads were generated, yielding a normalized dataset containing 1725 ASVs.

**Fig. 4:**
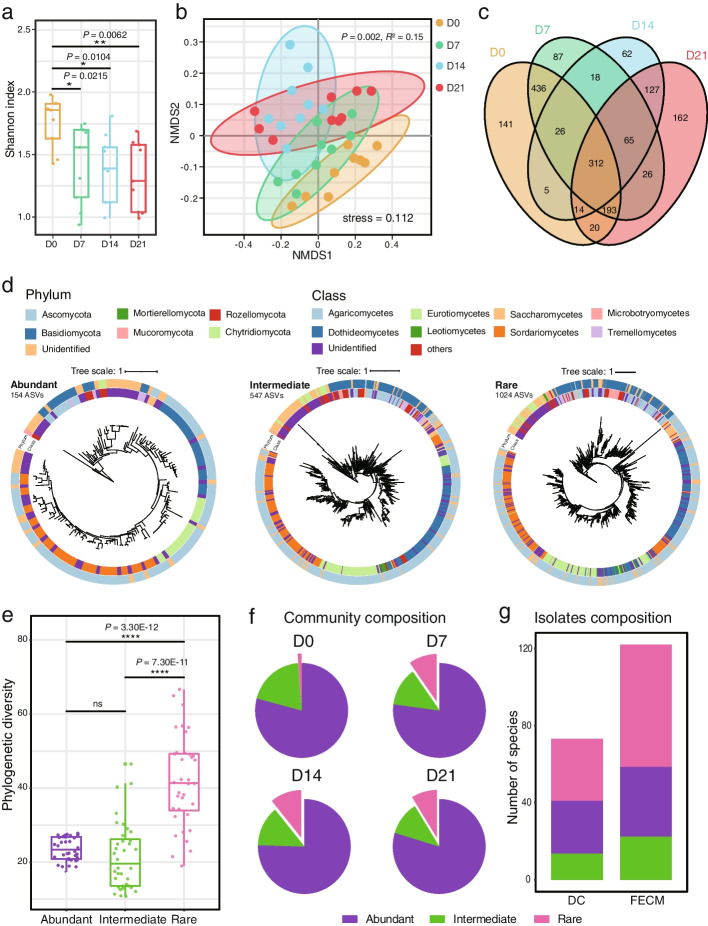
Composition of fungal communities in different stages of FECM of three sediment samples by means of high-throughput sequencing of fungal amplicons biomarker. **a** Shannon index of fungal communities. D0–D21 indicate 0, 7, 14, and 21 days of FECM, respectively. The significance of difference was determined by nonparametric Wilcoxon test. **b** NMDS of fungal communities based on weighted UniFrac metrics from four stages of enrichment cultivation. The significance of factors on community dissimilarity was tested with nested PERMANOVA based on weighted UniFrac distances. D0–D21 indicate 0, 7, 14, and 21 days of FECM, respectively. **c** Venn diagram of ASVs from different periods of enrichment cultivation. D0–D21 indicate 0, 7, 14, and 21 days of FECM, respectively. **d** Phylogenetic distribution of 154 abundant taxa, 547 intermediate taxa, and 1024 rare taxa (ASVs with a relative abundance above 0.1% across total sequences were considered as “abundant” taxa, those with relative abundances below 0.01% were considered as “rare” taxa, and those with relative abundances between 0.01 and 0.1% were “intermediate” ASVs). **e** Difference of phylogenetic diversity among abundant taxa, intermediate taxa, and rare taxa. The significance of difference was determined by nonparametric Wilcoxon test. **f** Pie plot showing the community compositions of each period of FECM based on the criteria of abundant taxa, intermediate taxa, and rare taxa. D0–D21 indicate 0, 7, 14, and 21 days of FECM, respectively. **g** Bar plot showing the isolates compositions between DC and FECM based on the criteria of abundant taxa, intermediate taxa, and rare taxa

The Shannon index of fungal community in day 0 samples was relatively higher than that of the later samples (Fig. 4a). Nonmetric multidimensional scaling (NMDS) based on the weighted UniFrac metrics showed that FECM influenced the beta diversity during the course of enrichment (Fig. 4b). In addition, PERMANOVA analysis also revealed the significant effect on the fungal community exerted by the different periods of FECM (*P* = 0.002, *R*^2^ = 0.15). Furthermore, new and unique ASVs were detected from different stages of enrichment (Fig. 4c).

All of the 1725 ASVs were divided into three groups, including 154 abundant taxa (accounting for 8.9% of the total ASVs), 547 intermediate taxa (31.7%), and 1024 rare taxa (59.4%) (Fig. 4d). The phylogenetic diversity index analysis showed that rare taxa have significantly higher phylogenetic diversity than abundant and intermediate taxa (Fig. 4e). All the three groups were dominated by members from phylum Ascomycota, with Sordariomycetes, Dothideomycetes, and Eurotiomycetes accounting for about 50% of total ASVs (Fig. 4d). Remarkably, phylogenetic relationships within most unidentified classes were closely related to phyla Chytridiomycota, Mucoromycota, Mortierellomycota, and Rozellomycota (Fig. 4d). The community composition at different enrichment stages changed with incubation time, especially the proportion of rare taxa increased significantly (Fig. 4f).

**Fig. 5:**
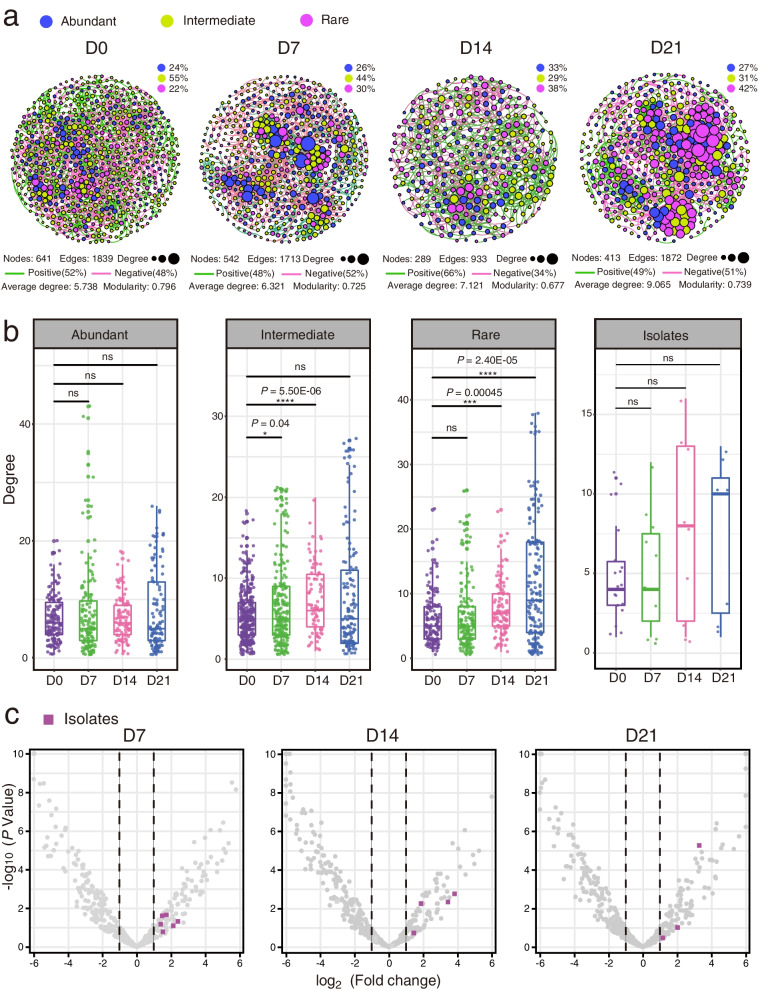
Fungal co-occurrence networks and volcano plot of different enrichment periods. **a** Fungal co-occurrence networks showing the assembly of fungal community during different periods of FECM. The nodes are colored according to abundant taxa, intermediate taxa, and rare taxa. Node size indicates the degree of connection. Edge color represents positive (green) and negative (red) correlations. **b** The degree of abundant taxa, intermediate taxa, rare taxa, and isolates showing the higher complexity of the rare taxa and isolates in the network with the extension of enrichment period. The significance of difference was determined by nonparametric Wilcoxon test. **c** Volcano plot illustrating the enrichment and depletion patterns of fungal microbiomes compared with 0-day enrichment period. Square nodes in purple indicate the isolated species obtained by increasing relative abundance by FECM

We further performed network analysis to investigate the co-occurrence patterns of fungal taxa at each enrichment stage. In the network analyses (Fig. 5a), nodes were assigned to abundant, intermediate, and rare taxa using the same criteria as in Fig. 4d. The networks of different enrichment stages were recorded by the number of edges and nodes, the average degree and the modularity (Fig. 5a). Intriguingly, with the extension of enrichment, the average degree of the network increased (5.738 in D0, 6.321 in D7, 7.121 in D14, and 9.065 in D21; Fig. 5a). Moreover, at the day 14, the number of nodes and edges was less than that of other stages, with the highest positive correlations (66%) and minimal modularity (0.677) between members (Fig. 5a). Additionally, we found that the proportion of rare taxa and their correlations with other taxa significantly increased in the network with the enrichment course (Fig. 5a and b). Moreover, the degree of isolates showed similar trend with rare taxa (Fig. 5b), although the data are limited compared to the ASVs.

### High efficiency of FiChip in situ cultivation in revealing rare dark matter fungi

A handmade FiChip was designed for in situ fungal cultivation (Fig. 1b). In this operation, a 50-fold dilution of the sediment suspension from two sediment samples (SZ-1 and SZ-2) ensures that most cells of the FiChips contain only one cell. After 1-month in situ cultivation, a total of 480 colonies were seen and picked up from 2496 wells, among which 475 colonies (99%) were considered single pure culture and the other five impure colonies were subcultured to 10 pure colonies. Meanwhile, a total of 220 strains were isolated from DPM using the same batch of two sediment samples.

**Fig. 6:**
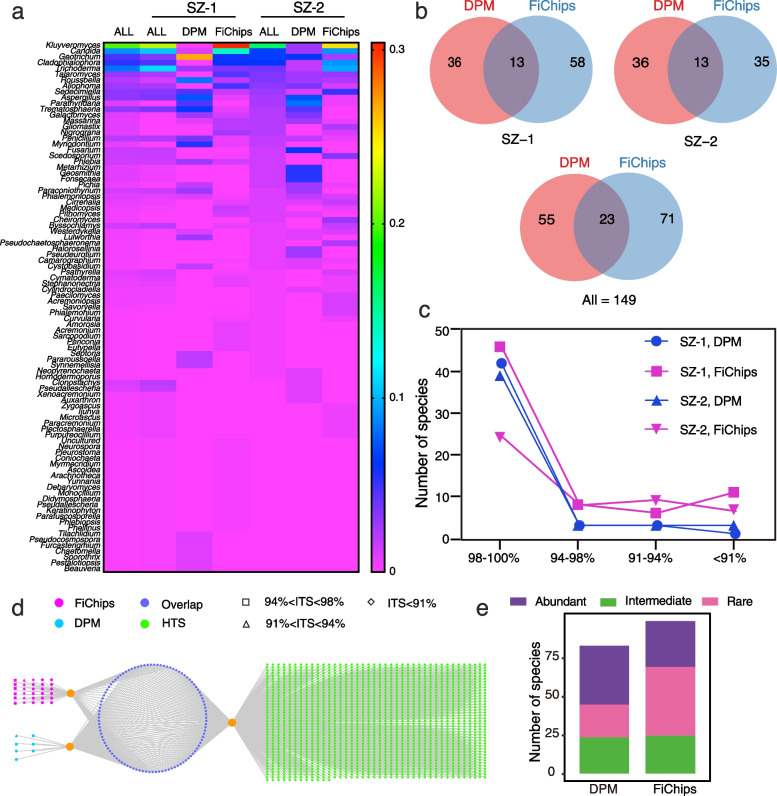
Comparative analyses among culturable fungi isolated by dilution-plate method (DPM), fungal isolation chips (FiChips), and high-throughput sequencing (HTS) from SZ-1 and SZ-2 sediment samples. **a** The heatmap for generic diversity from two sediment samples. The label ALL indicates the corresponding total generic diversity of the isolates. **b** Venn diagram of species between DPM and FiChips from two sediment samples. **c** Novelty of fungal strains grown in FiChips and DPM. The *x*-axis shows the sequence novelty, in percentage of diversion from the known species. The *y*-axis shows the number of species cultivated via each indicated method. Fungal strains were more novel as compared with strains cultivated using standard dilution-plate methods. **d** The interactive network of ITS2 locus sequenced from DPM, FiChips, and HTS. The nodes indicate different ITS2 genes. The nodes in green, pink, and blue indicate the ITS2 locus sequenced from HTS, FiChips, and DPM, respectively. The nodes in purple indicate the ITS2 locus sequenced from any two methods. Candidate novel species are indicated by different shape of nodes (square nodes indicate ITS sequence similarity between 94 and 98% comparing to the NCBI database, triangular nodes indicate similarity between 91 and 94%, and rhombic nodes indicate similarity below 91%). **e** The composition of species isolated from DPM and FiChips based on the criteria of abundant taxa, intermediate taxa, and rare taxa

A total of 705 fungal strains were isolated, with 220 and 485 isolated from DPM and FiChips respectively. Preliminary taxonomic assignments based on ITS sequence similarities showed that these isolates belonged to 149 species in 2 phyla, 8 classes, 27 orders, 54 families, and 91 genera (Fig. 2b, Additional file 2: Table S3), including 46 potential novel species, of which 16 have ITS sequence similarity between 94 and 98% compared to the closest hit in NCBI database, 15 with similarity between 91 and 94%, and 15 with similarity < 91%. The preliminary identification and ITS sequences of each species are listed in Additional file 2: Table S3. Among these isolates, the most abundant phylum was Ascomycota, with 678 isolates (representing 140 species) belonging to four main classes: Saccharomycetes (255 strains, 17 species), Sordariomycetes (185 strains, 60 species), Eurotiomycetes (116 strains, 29 species), and Dothideomycetes (102 strains, 25 species). Only 26 out of 704 isolates were classified as Basidiomycota, with 24 isolates belonging to Agaricomycetes (8 species) and 2 isolates belonging to Cystobasidiomycetes (1 species) (Fig. 2b). Furthermore, the heatmap of generic diversity from two sediment samples showed similar diversity among the high abundant genera (Fig. 6a).

A comparative analysis between FiChips and DPM was conducted. In this study, 94 species were obtained from FiChips, higher than that of 78 species from DPM (Figs. 2b and 6b, Additional file 2: Table S4). Moreover, for SZ-1 sample, only 13 species are overlapped among isolates obtained from DPM and FiChips, and this is the same case for SZ-2 sample (Fig. 6b, Additional file 2: Table S4). Preliminary identification of fungal strains showed that 44 and 13 potential novel species were respectively isolated from FiChips and DPM, suggesting much greater potential of FiChips in isolating rare and hitherto uncultured fungi (Figs. 2b and 6c). These findings indicated that species isolated by traditional DPM were limited, while species obtained from FiChips were highly unique and novel. These findings are consistent with Nichols [12] who reported that the iChip-based method enabled growth of a substantial fraction of bacterial collections with high phylogenetic novelty.

### Comparison among HTS, FiChips, and DPM

After quality control and removal of chimeras and singletons, a total of 1,590,328 reads were obtained from SZ-1 and SZ-2 sediment samples (each with three replicates). The remaining non-chimeric reads were assigned to 3488 ASVs. The number of fungal reads ranged from 30,022 to 151,395 among different samples after removal of non-fungal reads, resulting in a normalized dataset containing 1754 fungal ASVs.

To visualize the comparison among HTS, FiChips, and DPM, we conducted a local BLAST analysis using the ITS2 of 149 cultured species against ITS2 library of two sediment samples. Cultured species with 100% sequence similarity to the ITS2 libraries were considered the same species. A network graph was plotted based on ITS2 sequence for the shared taxa among the HTS, FiChips, and DPM (Fig. 6d). It is shown that most of the isolated species (75%) could be detected in the sequence library from HTS (Fig. 6d and Additional file 3: Figure S2). Seven species isolated from DPM could not be detected through HTS and FiChips, one of which is potential new species (Fig. 6d). By contrast, 30 species isolated from FiChips were not detected through HTS and DPM (Fig. 6d), and nearly half of which (13) were potential novel species (Fig. 6d). Our results indicated that fungal diversity in mangrove sediments far exceeds that could be revealed by DPM (Fig. 6d and Additional file 3: Figure S2). More species have been isolated by using FiChips, and notably, some of these species could not be detected even by high-throughput sequencing. Furthermore, most of these species are potential novel species, some of which may represent higher-rank novel taxa (Fig. 6d). In summary, our results showed that FiChips in situ cultivation is a promising protocol for culturing the rare and hitherto uncultured fungi from the mangrove sediments.

### Comparison between LSFI and NFCT

To further verify that new fungal culturomics techniques (NFCT) could effectively improve our understanding of species diversity and obtain previously uncultured fungi, we compared the fungal diversity obtained by large-scale fungal isolation (LSFI) from 66 samples and that obtained by NFCT from 5 of the 66 samples. Combined with 1365 isolates from five samples in this study, a total of 407 species were isolated from 66 sediment samples (Additional file 3: Figure S3, Additional file 2: Table S5). Preliminary taxonomic assignments based on ITS sequences similarities indicated that 407 species belonged to 3 phyla, 11 classes, 39 orders, 89 families, and 185 genera (Additional file 3: Figure S3, Additional file 2: Table S5), including 107 potential novel species (ITS sequence similarity less than 98% compared to the closest hits in NCBI database). The preliminary identification and ITS sequences of each species are listed in source data file.

Comparative analyses showed that 195 species were obtained from 5-sample fungal culturomics using NFCT, and 308 species were obtained from 66-sample LSFI using DPM (Additional file 3: Figure S4). Moreover, 94 species are found overlapped in isolates obtained from DPM and NFCT (Additional file 3: Figure S4). Preliminary identification of these fungal strains showed that 64 and 60 potential novel species was respectively isolated from NFCT and DPM, suggesting much greater potential of NFCT in isolating previously uncultured fungi (Additional file 3: Figure S4). These results showed NFCT have significantly improved efficiency of species discovery and higher novelty of isolated species.

### Taxonomy

In this study, 42 preliminarily identified potential novel species were isolated through FECM or FiChips, among which 34 belong to Ascomycota. Further phylogenetic analyses based on multi-locus sequences and morphological comparison showed that two of them actually represent known species, and seven failed to sporulate or subculture in laboratory and thus were bypassed in subsequent analyses. We therefore, based on molecular phylogenetic relationships and morphological features, formally described 25 novel species and introduced 8 new genera and 3 new families (Additional file 1).

## Discussion

Low-nutrient medium has been shown to be useful in cultivating previously uncultured microorganisms [27, 34]; thus, in our enrichment cultivation, we used 1/10-strength PDB (potato dextrose broth). The number of obtained species gradually decreased with the increasing of incubation time (Additional file 3: Table S6), possibly due to the reduction of available nutrients and the accumulation of metabolic waste [20]. However, we found that two strains (representing two species and accounting for 22% of the potentially novel species obtained after FECM cultivation) could not be further subcultured in the laboratory. These species may not be able to adapt to artificial media and may enter into a dormant state once again [21]. Further studies may be needed to identify the factors responsible for resuscitation.

The diversity of species isolated through direct culturing was overall much lower than that from enrichment cultivation, although a few species were specific (Fig. 3b, c). Approximately, one-fourth of total species could only be isolated from day 0 of FECM, and some of which represent novel taxa. Some of these original compositions of species might be inhibited in subsequent enrichment incubation, due to their inability to adapt changed conditions during the enrichment [20, 35]. Therefore, to explore a more real and comprehensive fungal diversity, multiple stages in the enrichment cultivation should be sampled and analyzed. However, the mechanism of enrichment cultivation remains poorly understood. A recent investigation through metatranscriptomics and comparative genomics surprisingly suggested that approximately 80% of the obtained bacterial isolates through enrichment cultivation were due to the resuscitation mechanism [27], but it remains unknown whether fungi present similar pattern in the enrichment cultivation.

To answer the above question, volcano plot was constructed to illustrate the enrichment and depletion patterns of the mycobiomes during the course of enrichment (Fig. 5c). We found that the relative abundance of only a few isolated species (13/125) was increased during enrichment cultivation (Fig. 5c), indicating that other mechanisms may played a greater role in isolating previously uncultured fungi, such as resuscitation [19, 27]. Future study may design experiments employing metatranscriptomic techniques to test this hypothesis.

In the co-occurrence network analysis of enrichment cultivation samples, the average degree increased with the extension of enrichment, indicating an enhanced mutual communication in the fungal community. Theoretical modeling and simulation data have suggested that microbial networks with proper ties of greater modularity, lower positive correlations, and higher negative correlations are more stable [36, 37]. Intriguingly, the lowest modularity and highest positive correlation were both observed in the day 14 community (Fig. 5a), indicating the most unstable network during the enrichment process, which is most likely a reflect of community reassembly induced by the nutrition decrease. The results of community reassembly have embodied in more frequent microbial interactions, higher network stability, and increased proportion and correlation of rare species, as shown in the microbial network at day 21.

Recently, Dai et al. [38] proposed a “hunger games” hypothesis in which the abundant taxa in the network predominate in the nutrient-sufficient environments, counteracting the biotic harshness generated by competitors. However, in oligotrophic environments, microbes are more likely to cooperate with each other to enable full utilization of nutrients [39]. In our network analyses, from day 0 to day 7, due to the sufficient nutrients, the degree and closeness centrality of abundant taxa increased significantly, fulfilling the hypothesis of “hunger games” (Fig. 5a and Additional file 3: Figure S5). Moreover, the fungal networks reached the highest degree and closeness centrality at day 21, suggesting that cooperation in fungal communities was enhanced with nutrient depletion (Fig. 5a).

Various in situ cultivation methods with similar basic principles have been applied to different environmental samples and have shown higher bacterial cultivation efficiencies compared with traditional methods [40]. Some previously uncultured, phylogenetically novel, and industrially important bacteria were isolated using these methods [41, 42]. However, few of these studies elucidated the mechanisms why in situ cultivation yields more phylogenetically novel and diverse microbial isolations than conventional method [12, 43]. Certainly, the mechanisms of growth promotion of fungi in the in situ cultivation also remain unknown.

FiChips enabled cultivating some unique fungi that have rarely been isolated through traditional approaches (Fig. 6b–d). One reasonable explanation is that the inoculated microorganisms obtained necessary growth elements from the natural environments that are absent in artificial media. Recently, Jung et al. [43] in their in situ cultivation of bacteria found that the potential growth initiation factors stimulate microbial resuscitation from a non-growth state. Furthermore, previous studies showed that only a small portion of bacteria isolated through in situ cultivation are filamentous actinomycetes [44]. Probably, the traditional diffusion chamber inoculated with a mix of agar and diluted environmental sample is not suitable for the growth of filamentous microorganisms. In our FiChips, each chamber was inoculated with agar and diluted sediment suspension separately, allowing fungal cells to form initiating filamentous colonies on the agar surface, thereby facilitating the growth of aerial hyphae.

Although in situ cultivation shows great advantage in mining previously uncultivated fungi, we found that eight strains (representing three species and accounting for 12% of potential novel species from in situ cultivation) could not be further subcultured in the laboratory. These fungal strains appeared to have lost their growth activity after several subculturing processes. Clearly, these fungi are yet not adapted to the conditions of artificial culture, and growing them in in situ cultures for multiple rounds may be useful for their domestication [42]. Future study should identify their key growth factors and test their effects in the subcultivation.

Although several in situ cultivation protocols have been shown to be able to improve the efficiency of microbial isolation and cultivation, these methods also have some limitations that need to be further optimized. Most media use agar as a solidifying agent, while in some cases, agar can inhibit microbial reproduction [45]. It has been shown that the number of colony varied by 1–3 orders of magnitude when using different curing agents, with the highest number obtained using cold glue [44] and silica gel being more preferable for oligocarbotrophic fungi [46]. The mechanism of microbial adaptation to colony formation on solid medium remains largely unknown, which is one of the main reasons why most microorganisms are not culturable in the laboratory [47]. Therefore, it is necessary to further develop new cultivation methods to explore the unknown diversity of bacteria and fungi. For example, the recently developed diffusion bioreactor uses liquid media for in situ cultivation, allowing for the growth of a variety of previously uncultured soil bacteria [48]. However, many microorganisms rely on the products of their co-trophic partners for growth [19]^.^ In this case, the use of an isolation chip that separates an individual microorganism in a fully sealed chamber may actually prevent its successful cultivation. Although diffusion bioreactor provides communication between microbial communities, the slow-growing microbes may still be inhibited by fast-growing microbes. To overcome this limitation, a previous study designed a nanoporous microscale microbial incubator system, which integrated microfluidic and membrane diffusion-based technologies [49]. Once sealed, each chamber physically separates individual cells, but the permeable chamber walls allow the transfer of growth factors and signaling compounds among all cells in the slide sheet by passive diffusion. Although the nanoporous microscale microbial incubator system appears to have strong potential for cultivating interesting co-trophic organisms in the field, no successful examples have been published [50].

Rare taxa are generally defined as taxa with low relative abundance in the microbial communities due to poor competitive abilities, most of which are slower in growth rate [51]. Under in vitro conditions, slow-growing microorganisms are often inhibited by the nutrient starvation caused by the fast-growing microorganisms [52]. Thus, in situ cultivation not only provides more natural environmental conditions but also largely avoids competition and inhibition among microorganisms. In this study, our in situ cultivation protocol is potentially a very useful alternative to obtaining the rare portion of the fungal diversity from mangrove sediments, as compared to the traditional methods.

In the FECM, some rare fungal taxa were enriched but could not subsequently be isolated from the five culture media. One possible reason is the differences in the environmental factors between the enrichment conditions and the culture conditions, such as oxygen concentration and nutrient composition [27]. Another reason may be that some fungi need to synergize with other fungi or bacteria to grow [16]. Our results showed that fungal diversity and composition change during the enrichment process, especially the emergence of more rare taxa. This suggests that mangrove sediment is a good “seed bank” of high fungal diversity [26], and that a large number of rare species in the sediment are barely viable until the emergence of proper nutrients [53, 54]. Furthermore, FECM altered the assembly of fungal communities, especially for rare taxa (Fig. 5a), possibly by facilitating their communication in the community (dormancy resuscitation).

Rare taxa are often overlooked [55, 56], but accumulating evidences suggested that rare taxa may play an important role in maintaining the stability of fungal communities and their ecological functions in crop ecosystems [57, 58]. In our study, both FECM and FiChips could significantly increase the number of isolated rare taxa (Figs. 4g and 6e). Further applications of these protocols in a variety of environments are expected to significantly advance our understanding on the ecological functions of rare fungal taxa.

The diversity and distribution of marine microbiota have not been unveiled until recently [59, 60]. Mangrove sediments are the main habitat for marine microorganisms, and recent studies have shown a higher proportion of unknown microorganisms in mangrove sediments [61]. Comparison between LSFI and NFCT showed our FECM and FiChips significantly improved the efficiency and novelty of isolated species (Additional file 3: Figures S3 and S4). Moreover, the time and labor costs of NFCT are lower; thus, they have advantages in mining rare dark matter fungi.

Although the omics-driven findings can greatly improve our understanding of microbial life, it remains important to isolate and culture microorganisms from these previously uncultured lineages to test the omics-based predictions of their phenotypic traits, physiological functions, and ecological roles in ecosystems. Taxonomic study on our isolates revealed that seven new species obtained from FiChips represent two newly established families in Hypocreales. Interestingly, the spore sizes of these fungal species are much smaller than most species in Hypocreales, being only about 1.5 µ in diameter. When more different environments are studied using culturomics approaches, mycologists will be able to provide important amendments for the fungal tree of life.

Although this study shows that FECM and FiChips outperform conventional DPM in mining rare dark matter fungi, almost all obtained fungal isolates belong to the two most common phyla, i.e., Ascomycota and Basidiomycota. One explanation is that Ascomycota and Basidiomycota are dominant in the mangrove sediment (Fig. 4d). However, Chytridiomycota also occupies a certain proportion of fungal communities in mangrove sediments (Fig. 4d). Previous studies have revealed that most members in Chytridiomycota are parasitic fungi, some of which could be isolated by cellulosic bait and algae [62, 63]. Therefore, future design of enrichment cultivation and in situ cultivation with cellulosic bait and algae may facilitate the isolation and culture of Chytridiomycota, thus updating knowledge of these early diverging fungi in mangrove sediments.

The conventional dilution plate method only allowed for the cultivation of 2.57 to 4.35% of ASVs in the original samples. However, when fungal culturomics techniques were combined, the cultivation efficiency increased to 5.87 to 7.25% of ASVs. This improved isolation efficiency led to the discovery of a higher number of novel fungi. By increasing the efficiency of fungal cultivation, we can gain a better understanding of the nutritional features and bioremediation capabilities of fungi in mangrove ecosystems. For instance, Gupta and Das found that *Aspergillus* sp., isolated from mangroves, could provide soluble phosphorus not only for themselves but also for other organisms [64]. Furthermore, *Aspergillus* sp. and *Alternaria alternata*, isolated from the mangrove sediments, have demonstrated the ability to remove chromium, lead, and cadmium from polluted mangrove ecosystems [65, 66]. Therefore, we believe that further investigation into the metabolic and nutritional interactions of fungi in mangrove sediment is necessary. Isolation and identification of potential new species from FECM and FiChips are valuable for mining secondary metabolites and inferring their ecological functions. Teixobactin reported in 2015 was a new class of antibiotics discovered in nearly 30 years, which was derived from a new bacterium isolated from in situ cultivation that can kill superbugs [40, 67, 68]. Given the increasing importance of fungi in the discovery of new antibiotics, the new species described in this study and more in future will provide valuable new genetic resources for mining more and new natural compounds.

## Conclusion

In this study, we developed protocols of enrichment cultivation and in situ cultivation for high-efficiency isolation of fungi from mangrove sediments. Our workflow enables large-scale cultivation and identification of fungi, which greatly improves our understanding of culturable mycobiota in mangrove sediments. The fungi isolated from five sediment samples through the two newly developed techniques represent 193 species and 103 genera, much higher than that isolated from five sediment samples through traditional DPM (131 species and 72 genera). Meanwhile, this study increases the known species diversity of fungi in mangrove sediments by 94%, compared to the 193 species reported by 2021 [28]. Through FECM, FiChips, and DPM, a total of 75 potential new species were isolated from five sediment samples, and 62 out of which were from FECM and FiChips, indicating an advantage in acquiring rare fungi. Furthermore, FECM can significantly influence the composition of fungal communities with time, as well as the community assembly of rare taxa. These results suggested that FECM and FiChips have significant advantages in cultivating rare and dark matter fungi.

Moreover, culture-dependent and culture-independent approaches revealed different fungal community compositions from mangrove sediments, indicating the need for complementary approaches to better understand fungal diversity. Future studies are expected to reveal the mechanism through which previously uncultured fungi acquire recovery and resuscitation by combining currently applied methods with metatranscriptomic data.

## Methods

### Sample collection

Three mangrove sediment samples used for FECM and DPM were collected from National Mangrove Nature Reserve of Zhanjiang, Guangdong province, China (ZJ1-1, 21°06′15″ N 110°18′47″ E; ZJ2-1, 21°06′03″ N 110°18′25″ E; ZJ3-1, 21°05′27″ N 110°16′57″ E) on 20 November 2019 (Additional file 3: Figure S6a). Two mangrove sediment samples for FiChips and DPM were collected from National Mangrove Nature Reserve of Futian, Shenzhen, Guangdong province, China (SZ-1, 22°31′39″ N 113°59′52″ E and SZ-2, 22°31′36″ N 113°59′52″ E) on 27 September 2020 (Additional file 3: Figure S6b). All sediment samples were collected from the depth of 0–10 cm by a surface sediment sampler (Additional file 3: Figure S6c). Other 61 sediment samples used for large-scale fungal isolation (LSFI) of DPM were collected from five mangrove nature reserves (National Mangrove Nature Reserve of Zhanjiang, National Mangrove Nature Reserve of Futian, Mangrove Nature Reserve of Dianbai, Mangrove Nature Reserve of Zhuhai, and Mangrove Nature Reserve of Enping) in Guangdong province, China. For each sediment sample, five replicates were respectively collected and mixed together. Following collection, each sample was placed into two sterile sampling bags, which was preserved at 4 °C until they were processed (within 24 h), and another one was transported on dry ice to the laboratory and stored at − 80 °C for DNA extraction. Meanwhile, seawater near the sediment samples (ZJ-1, ZJ-2, ZJ-3, SZ-1, and SZ-2) was collected for subsequent experiments.

### Fungal isolation

Five different culture media supplemented with 3.5% sea salt, 0.005% ampicillin, and 0.005% streptomycin were prepared for DPM: i.e., PDASS (potato dextrose agar-sea salt), MEASS (malt extract agar-sea salt), MMSS (Martin medium-sea salt), CMASS (corn meal agar-sea salt), and CDASS (Czapek Dox Agar-sea salt) [30]. All the sediment samples were diluted for 50 times with sterile seawater and spread onto five different culture media. Three replicates were set up for each isolation medium.

A low-nutrient medium was used for FECM, which consisted of the following ingredients in 1-L sterilized seawater: 0.05% potato starch, 0.15% dextrose, 0.1% peptone, 0.05% NaCl, 0.005% ampicillin, and 0.005% streptomycin. Enrichment incubation was performed at 25 °C for 21 days in separate 500-mL sterile flasks (filled with 400-mL medium and 20 g of sediment sample, Fig. 1a). Thirty-six subsamples of enrichment cultures were collected from flasks on the 0th, 7th, 14th, and 21st days for fungal isolation and amplicon sequencing (Fig. 1a). The subsamples were diluted, and aliquots of the homogenate were subsequently inoculated on five culture media (PDASS, MEASS, MMSS, CMASS, and CDASS) with 3.5% sea salt to isolate fungi. A detailed protocol is described in Additional file 3.

For in situ cultivation, we designed a modified handmade fungal isolation chip (FiChip) [42] suitable for fungal isolation. It consisted of two sterile elements: a customized polypropylene plate with 96 through-holes and two precut rectangular pieces of 0.22-µm-pore-size nylon membrane (Fig. 1b). In each cell of the FiChips, 100-µL seawater agar (prepared with 1-L seawater, 15-g agar, 50-mg ampicillin, and 50-mg streptomycin) was firstly added, and 10-µL sediment suspension (diluted for 50 times with sterile seawater) was subsequently added after agar solidification. A stainless steel mesh frame with 15 FiChips was placed at each sampling site for 1-month incubation. A detailed protocol is described in the supplementary file.

All the pure cultures isolated from different methods were incubated at room temperature (25 ± 2 °C) for 2–4 weeks. All fungal strains were stored in sterile water with 3.5% sea salt at 4 °C for further studies and have been deposited in LC culture collection (personal culture collection held at the lab of Dr. Lei Cai in State Key Laboratory of Mycology, IMCAS).

### DNA extraction and amplification sequencing

Genomic DNA was extracted from fungal mycelia of each isolate growing on PDA, using a modified CTAB protocol as described in Liu et al. [69]. The full-length ITS (5.8S nuclear ribosomal RNA gene with the two flanking internal transcribed spacer) was amplified using the forward primer ITS1 (5′-TCCGTAGGTGAACCTGCGG-3′) or ITSIF (5′-CTTGGTCATTTAGAGGAAGTAA-3′) and the reverse primer ITS4 (5′-TCCTCCGCTTATTGATATGC-3′) [70]. The PCR amplifications were performed in a reaction mixture consisting of 12.5 μL 2 × Taq PCR Master Mix (Vazyme Biotech Co., Ltd., Nanjing, China), 1 μL each of 10 μM primers, and 1**–**10 ng genomic DNA, adjusted to a final volume of 25 μL with distilled deionized water. The reaction condition was as follows: 95 °C for 5 min, 35 cycles at 95 °C for 30 s, 52 °C for 30 s, 72 °C for 15 s, 72 °C for 10 min, and held at 4 °C. Sequencing was conducted by SinoGenoMax Company Limited (Beijing, China). Consensus sequences were obtained using SeqMan of the Lasergene software package v. 14.1 (DNAstar, Madison, WI, USA).

Total genomic DNA was extracted for two sediment samples used for in situ cultivation and 36 subsamples at different periods of enrichment incubation (0, 7, 14, and 21 days), using a FastDNA® Spin Kit following the manufacturer’s instructions (MP Biomedicals, Solon, OH, USA). The fungal ITS2 region was amplified using primers fITS7 (5′-GTGARTCATCGAATCTTTG-3′) and ITS4 (5′-TCCTCCGCTTATTGATA TGC-3′) [35]. The PCR reaction for each sample was performed with 0.2-μM forward primer, 0.2-μM reverse primer, 10 ng of template DNA, and 12.5 μL of 2 × Taq Plus Master Mix (Vazyme Biotech Co., Ltd., Nanjing, China). The reaction condition was as follows: 95 °C for 3 min, 36 cycles at 95 °C for 30 s, 55 °C for 30 s and 72 °C for 60 s, 72 °C for 10 min, and held at 4 °C. Amplicon libraries were sequenced on the Illumina NovaPE250 platform (MAGIGENE Biological Company, Guangdong, China).

### Sequencing data processing

The full-length ITS sequences of all isolates used for classification analysis were trimmed and filtered using MEGA 7.0 [71]. All obtained sequences were BLASTn searched in NCBI database and assigned to potential genera and species. The strains with the closest similarities of ITS gene sequences higher than 98% were tentatively assigned a name, while those below 98% were subjected to further polyphasic identification. To do further analyses of comparison with amplicon sequencing data, we extracted ITS2 regions from the full-length ITS gene sequences of all strains.

For amplicon data, the ITS2 sequences were processed using USEARCH 10.0 and VSEARCH 2.14 software respectively [72, 73]. All ITS paired-end reads were quality filtered using the *fastq_filter* command and joined by the *fastq_mergepairs* command. Chimeric sequences were detected and removed using UNITE CHIME reference database [74]. The non-chimeric sequences were clustered into different amplicon sequence variants (ASVs) with 100% similarity level using unoise3 command [72, 75]. Then, the un-fungal sequences were removed using ITSx software [76]. The remaining sequences were reassigned to formulate a final ASV table using the *otutab* command. The representative sequences of each fungal ASV were blasted against the UNITE database to assign taxonomic annotation [74].

### Phylogenetic analyses and statistical analyses

To reveal the fungal diversity and visualize correlation analysis of cultured fungi across different approaches, maximum likelihood (ML) method was used to construct phylogenetic tree. ITS2 alignment was generated using MUSCLE [77] and trimmed using trimAL [78]. ML analyses were performed using IQ-TREE, and the parameter MFP (ModelFinder Plus) was set up for automated testing and selection of the best alternative model [79]. The robustness of branches was assessed by bootstrap analysis with 1000 replicates. An online tool iTOL (http://itol.embl.de) [80] and table2itol.R script (https://github.com/mgoeker/table2itol) were used for the display, manipulation, and annotation of phylogenetic trees.

The statistical analyses for fungal community composition and diversity were performed using the vegan package as implemented in the R environment (version 3.6.1) unless stated otherwise [81, 82]. The ASV tables were rarefied to the smallest number of reads among all samples using the *rrarefy* command to allow comparison on an equal basis before calculating the diversity indices. Subsequent analysis of alpha diversity and beta diversity were all performed based on this output normalized data.

Alpha diversity of enrichment samples from different periods was calculated for Shannon–Wiener indexes via the diversity command [82]. The differences among samples of each period of enrichment cultivation were tested using Wilcoxon rank-sum tests. The significant changes in beta diversity at each period of enrichment cultivation were estimated using the weighted UniFrac metrics [83] and visualized with the nonmetric multidimensional scaling (NMDS) ordinations. The significance of enrichment factors on fungal community was tested with permutational multivariate analysis of variance (PERMANOVA) using “adonis” vegan R package [84].

To estimate the efficiency of optimized culturomics approaches in the cultivation of rare taxa, ASVs were divided into three groups according to their relative abundance. ASVs with a relative abundance above 0.1% across total sequences were considered as “abundant” taxa, those with relative abundances below 0.01% were considered as “rare” taxa, and those with relative abundances between 0.01 and 0.1% were “intermediate” ASVs [85]. To visualize the phylogenetic relationships, phylogenetic tree based on ML analysis was constructed as mentioned above. A local BLAST analysis of ITS2 fragments of isolated species against ITS2 libraries of different stages of enrichment was conducted, and 100% sequence similarity with ITS2 libraries was considered as the same species.

To visualize the dynamic changes of fungal communities among different enrichment periods, a co-occurrence network analysis based on Spearman correlation scores was performed using the CoNet app in Cytoscape v3.7.1 [86, 87], and only the Spearman’s correlation coefficient (ρ) above 0.70 and *P* < 0.05 was considered as robust. The Benjamini–Hochberg procedure was performed to minimize false-positive signals before network construction [88]. The networks were visualized using Gephi 0.9.2 [89].

All statistical analyses were carried out in R (http://www.r-project.org). Nonparametric statistical tests have been run to evaluate the significance of difference (Wilcoxon test, *P* < 0.05).

### Identification and description of novel taxa

All potential novel taxa of filamentous Ascomycota isolated through FECM and FiChips were further identified based on multi-locus phylogenetic analyses and morphological comparison with known species. For molecular analyses, ITS, the small subunit (SSU) rDNA, the large subunit (LSU) rDNA, the translation elongation factor 1-alpha (*tef1*), RNA polymerase I subunit (*rpb1*), RNA polymerase II subunit (*rpb2*), β-tubulin (*tub2*), and calmodulin (*cam*) regions were amplified using primer pairs ITS1/ITS4 [70], NS1/NS4 [70], LR0R/LR5 [90], 983F/2218R [91], RPB1-F7/RPB1-G2R [92], RPB2-5F2/fRPB2-7cR [93, 94], Bt2a/Bt2b [95], and CF1/CF4 [96], respectively. Amplification reactions were performed in a 25-μL reaction volume including 0.2-μM forward primer, 0.2-μM reverse primer, 10 ng of template DNA, and 12.5 μL of 2 × Taq Plus Master Mix (Vazyme Biotech Co., Ltd., Nanjing, China). The detailed information of primers and reaction conditions is shown in Additional file 3: Table S7. Sequencing reactions were performed by SinoGenoMax Company Limited (Beijing, China).

To determine the phylogenetic relationships of novel species, analyses were performed based on three to five loci according to different fungal taxa. Alignments for each locus were generated using MAFFT (http://www.ebi.ac.uk/Tools/msa/maft/) [97] and then manually edited in MEGA v. 7.0 when necessary. Alignments of each loci were concatenated and used in the subsequent phylogenetic analysis. Ambiguously, aligned regions were excluded from all analyses.

Maximum likelihood (ML) and Bayesian inference (BI) methods were used to construct the phylogenetic trees. ML analyses were performed using RAxML-HPC v. 8.2.7 [98] with 1000 replicates under the GTR-GAMMA model. BI analyses were performed with MrBayes v. 3.2.1 [99]. Appropriate nucleotide substitution models and parameters were calculated using jModelTest v. 2.1.7 [100, 101] Phylogenetic trees were visualized using FigTree v1.4.0 [102].

For morphological studies, single spore isolations were obtained for the represented strains of novel species and then according to different species transferred to new plates of different media (PDA, PDASS, OA, MEA, CAM, YES, DG18, CYA, and SNA) for incubation at room temperature (25 ± 2 °C). Growth rates were evaluated after 7 days, while slow growing strains were measured after 2 weeks or even 4 weeks. Colony colors on the surface and reverse of different media were assessed according to the Methuen Handbook of color. Morphological characters were observed using a Nikon 80i microscope with differential interference contrast (DIC) illumination. Measurements for each structure were made as described in Zhang et al. [103].

### Supplementary Information

**Additional file 1.** Descriptions of novel taxa proposed in this study. (PDF 9407 kb)

** Additional file 2:** **Table S1.** The detailed information of each species on phylogenetic tree based on FECM. **Table S2.** Species on phylogenetic tree isolated from ZJ-1, ZJ-2 and ZJ-3 sediment samples during different periods of FECM. **Table S3.** The detailed information of each species on phylogenetic tree based on* in situ* cultivation. **Table S4.** Species on phylogenetic tree isolated from SZ-1 and SZ-2 sediment samples through DPM and FiChips. **Table S5.** The detailed information of each cultured species on phylogenetic tree based on isolation of 66 sediment samples.(XLSX 42965 kb)

** Additional file 3.** The extended experimental procedures of fungal enrichment culture method (FECM) and *in situ* cultivation using fungal isolation chips (FiChips) and supplementary files. **Supplementary Fig. 1.** The heatmap for the diversity of species from three sediment samples during each stage of enrichment incubation (color ranging represents strains number). D0, D7, D14, and D21 indicate the isolation results of FECM on days 0, 7, 14 and 21, respectively. Source data are provided as a Source data file. **Supplementary Fig. 2.** The heatmap for the diversity of species from sediment samples SZ-1 and SZ-2 (color ranging represents relative abundance (%)). HTS, DPM, and FiChip indicate the results of high through-put sequencing, dilution-plate method, and *in situ* cultivation, respectively. Source data are provided as a Source data file. **Supplementary Fig. 3.** Phylogenetic tree of species isolated from 66 sediment samples. The phylogenetic tree was constructed from the full-length ITS gene sequences. Major phylum names are indicated and marked in different colors. Red branches on the trees indicate that the species was isolated only from new fungal culturomics techniques (FECM and FiChip). Candidate novel species are indicated by stars (blue stars indicate ITS sequence similarity between 94% and 98% comparing to the NCBI database, green stars indicate similarity between 91% and 94%, and red stars indicate similarity below 91%). In addition, detailed information of each species is shown in Additional file2: Table S5. **Supplementary Fig. 4.** Venn diagram between new fungal culturomics techniques (NFCT) and large-scale fungal isolation (LSFI). (a). Comparison of species diversity between NFCT and LSFI. (b). Comparison of the number of potential novel species between NFCT and LSFI. **Supplementary Fig. 5.** Comparison of node-level topological features in Fig. 5a (degree and closeness centrality) demonstrating the high degree and closeness centrality for the unidentified and other taxa at class level with the extension of enrichment period. **Supplementary Fig. 6.** Scenes of visited mangrove forests in Guangdong province, China. a. National Mangrove Nature Reserve of Zhanjiang. b. National Mangrove Nature Reserve of Futian Shenzhen. c. A surface sediment sampler. **Supplementary Table 6.** Cultivable fungal numbers at different periods of enrichment cultivation. **Supplementary Table 7.** Primers and PCR programs used in this study. **Supplementary Table 8.** Strain and sequence accession numbers of new species.

## Data Availability

The raw data of ITS2 datasets generated in this study have been deposited in the Sequence Read Archive under accession numbers BioProject PRJNA951956. For novel species, sequences generated in this study are deposited in GenBank (Additional file 3: Table S8), typifications, and novel taxonomic descriptions in fungal names (https://nmdc.cn/fungalnames/). Type specimens of new species were deposited in the Herbarium of Microbiology, Academia Sinica (HMAS), and ex-type living cultures were deposited in the China General Microbiological Culture Collection Center (CGMCC).
