# Supplementary material for: Application of culturomics in fungal isolation from mangrove sediments
Source: Microbiome. 2023 Dec 11;11:272. doi: 10.1186/s40168-023-01708-6 (PMC10712113; doi:10.1186/s40168-023-01708-6)
Supplement: Supplementary file 2 — Additional file 1. Descriptions of novel taxa proposed in this study. (PDF 9407 kb) [file 40168_2023_1708_MOESM1_ESM.pdf]

Additional file 1: Descriptions of novel taxa proposed in this study

Table of contents

Phylum Ascomycota Caval.-Sm., Biological Reviews Cambridge 73: 247 (1998)

Class Dothideomycetes O.E. Erikss & Winka, Myconet 1(1): 5 (1997)

Subclass Dothideomycetidae P.M. Kirk, P.F. Cannon, J.C. David & Stalpers ex C.L. Schoch, Spatafora, Crous & Shoemaker, Mycologia 98(6): 1045 (2007) [2006]

Capnodiales Woron., Annls Mycol. 23(1/2): 177 (1925)

Extremaceae Quaedvl. & Crous, Fungal Systematics and Evolution 3: 127 (2019)

Pseudocastanedospora M. Li, M. Raza & L. Cai, ***gen. nov.***

Pseudocastanedospora guangdongensis M. Li, M. Raza & L. Cai, ***sp. nov.***

Subclass Pleosporomycetidae C.L. Schoch, Spatafora, Crous & Shoemaker, Mycologia 98(6): 1048 (2007) [2006]

Pleosporales Luttr. ex M.E. Barr, Prodr. Cl. Loculoasc. (Amherst): 67 (1987)

Macrodiplodiopsidaceae Voglmayr, Jaklitsch & Crous, IMA Fungus 6(1): 178 (2015)

Pseudochaetosphaeronema Punith., Nova Hedwigia 31(1-3): 126 (1979)

Pseudochaetosphaeronema irregulare M. Li & L. Cai, ***sp. nov.***

Roussoellaceae Jian K. Liu, Phook., D.Q. Dai & K.D. Hyde, Phytotaxa 181(1): 7 (2014)

Neoroussoella Jian K. Liu, Phook. & K.D. Hyde, Phytotaxa 181(1): 21 (2014)

Neoroussoella sedimenticola M. Li & L. Cai, ***sp. nov.***

Nothoroussoella M. Li & L. Cai, ***gen. nov.***

Nothoroussoella irregularis M. Li & L. Cai, ***sp. nov.***

Class Eurotiomycetes O.E. Erikss & Winka, Myconet 1(1): 6 (1997)

Subclass Eurotiomycetidae Tehler, Cladistics 8: 236 (1988)

Eurotiales G.W. Martin ex Benny & Kimbr., Mycotaxon 12(1): 23 (1980)

Aspergillaceae Link, Abh. dt. Akad. Wiss. Berlin: 165 (1826) [1824]

Penicillium Link, Mag. Gesell. naturf. Freunde, Berlin 3(1–2): 16 (1809)

Penicillium limoniforme M. Li & L. Cai, ***sp. nov.***

Penicillium sedimenticola M. Li & L. Cai, ***sp. nov.***

Talaromyces C.R. Benj., Mycologia 47(5): 681 (1955)

Talaromyces ellipsoideus M. Li & L. Cai, ***sp. nov.***

Talaromyces phialiformis M. Li & L. Cai, ***sp. nov.***

Onygenales Cif. ex Benny & Kimbr., Mycotaxon 12(1): 8 (1980)

Apinisiaceae M. Li, M. Raza & L. Cai, ***fam. nov.***

Apinisia La Touche 1968

Apinisia keratinophilum (Samson & Polon) M. Li & L. Cai, ***comb. nov.***

Apinisia pyriforme M. Li, M. Raza & L. Cai, ***sp. nov.***

Arachnotheca Arx, Persoonia 6(3): 376 (1971)

Arachnotheca pulvereum M. Li, M. Raza & L. Cai, ***sp. nov.***

Onygenaceae Berk., Intr. crypt. bot. (London): 272 (1857)

Auxarthron G.F. Orr & Kuehn, Can. J. Bot. 41: 1439 (1963)

Auxarthron pyriforme M. Li, M. Raza & L. Cai, ***sp. nov.***

Class Sordariomycetes O.E. Erikss & Winka, Myconet 1(1): 10 (1997)

Subclass Hypocreomycetidae O.E. Erikss & Winka, Myconet 1(1): 6 (1997)

Hypocreales Lindau, Nat. Pflanzenfam., Teil. I (Leipzig) 1(1): 343 (1897)

Acremoniopsiaceae M. Li, M. Raza & L. Cai, ***fam. nov.***

Nothoacremoniopsis M. Li, M. Raza & L. Cai, ***gen. nov.***

Nothoacremoniopsis irregularis M. Li, M. Raza & L. Cai, ***sp. nov.***

Nothoacremoniopsis sedimenticola M. Li, M. Raza & L. Cai, ***sp. nov.***

Phaeocollarina M. Li, M. Raza & L. Cai, ***gen. nov.***

Phaeocollarina guttulata M. Li, M. Raza & L. Cai, ***sp. nov.***

Bionectriaceae Samuels & Rossman, Stud. Mycol. 42: 15 (1999)

Pseudosynnemellisia M. Li, M. Raza & L. Cai, ***gen. nov.***

Pseudosynnemellisia favida M. Li, M. Raza & L. Cai, ***sp. nov.***

Sedecimiellaceae M. Li, M. Raza & L. Cai, ***fam. nov.***

Sedecimiella K.L. Pang, Alias & E.B.G. Jones, in Pang, Alias, Chiang, Vrijmoed & Jones, Bot. Mar. 53(6): 495 (2010)

Sedecimiella alba M. Li, M. Raza & L. Cai, ***sp. nov.***

Sedecimiella funiculosus M. Li & L. Cai, ***sp. nov.***

Sedecimiella minutispora (Sukapure & Thirum.) M. Li & L. Cai, ***comb. nov.***

Sedecimiella subulata M. Li & L. Cai, ***sp. nov.***

Heteroacremonium M. Li, M. Raza & L. Cai, ***gen. nov.***

Heteroacremonium album M. Li & L. Cai, ***sp. nov.***

Heteroacremonium rugosum M. Li, M. Raza & L. Cai, ***sp. nov.***

Nectriaceae Tul. & C. Tul., Select. fung. carpol. (Paris) 3: 3 (1865)

Fusarium Link, Mag. Gesell. naturf. Freunde, Berlin 3(1-2): 10 (1809)

Fusarium aseptatum M. Li & L. Cai, ***sp. nov.***

Microascales Luttr. ex Benny & R.K. Benj., Mycotaxon 12(1): 40 (1980)

Halosphaeriaceae E. Müll. & Arx ex Kohlm., Can. J. Bot. 50: 1951 (1972)

Okeanomyces K.L. Pang & E.B.G. Jones, Bot. J. Linn. Soc. 146(2): 228 (2004)

Okeanomyces guttulatus M. Li, M. Raza & L. Cai, ***sp. nov.***

Subclass Lulworthiomycetidae Dayar., E.B.G. Jones & K.D. Hyde, Fungal Diversity 72: 208 (2015)

Lulworthiales Kohlm., Spatafora & Volkm.-Kohlm., Mycologia 92(3): 456 (2000)

Paramoleospora M. Li & L. Cai, ***gen. nov.***

Paramoleospora guttulata M. Li & L. Cai, ***sp. nov.***

Subclass Sordariomycetidae O.E. Erikss. & Winka, Myconet 1(1): 10 (1997)

Coniochaetales Huhndorf, A.N. Mill. & F.A. Fernández, Mycologia 96(2): 378 (2004)

Coniochaetaceae Malloch & Cain, Can. J. Bot. 49: 878 (1971)

Coniochaeta (Sacc.) Cooke, Grevillea 16 (no. 77): 16 (1887)

Coniochaeta aurantiaca M. Li & L. Cai, ***sp. nov.***

Sordariales Chadef. ex D. Hawksw. & O.E. Erikss., Syst. Ascom. 5(1): 182 (1986)

Schizotheciaceae Y. Marin & Stchigel, Microorganisms 8(9, no. 1430): 24 (2020)

Neomorinagamyces M. Li & L. Cai, ***gen. nov.***

Neomorinagamyces pyriformis M. Li & L. Cai, ***sp. nov.***

Phylum Ascomycota Caval.-Sm., Biological Reviews Cambridge 73: 247 (1998)

Class Dothideomycetes O.E. Erikss & Winka, Myconet 1(1): 5 (1997)

Subclass Dothideomycetidae P.M. Kirk, P.F. Cannon, J.C. David & Stalpers ex C.L. Schoch, Spatafora, Crous & Shoemaker, Mycologia 98(6): 1045 (2007) [2006]

Capnodiales Woron., Annls mycol. 23(1/2): 177 (1925)

Extremaceae Quaedvl. & Crous, Fungal Systematics and Evolution 3: 127 (2019)

Extremaceae was established by Quaedvlieg et al. (2014) to accommodate teratosphaeriaceous asexual fungi occurring in extreme habitats. Currently, the members of the family are morphologically and ecologically highly heterogeneous, being filamentous or yeast-like. Morphologically, *Extremaceae* is characterized by sympodially proliferating conidiogenous cells and 1-2 transverse septate conidia (Quaedvlieg et al. 2014). Here, we introduce a new genus *Pseudocastanedospora* in *Extremaceae* (Fig. SD-1).

Pseudocastanedospora M. Li, M. Raza & L. Cai, ***gen. nov.***

Fungal Names: FN571495

Etymology: Referring to the close phylogenetic relationship with the genus *Castanedospora.*

Asexual morph *Conidiophores* solitary, terminal, lateral or intercalary on the hyphae, frequently branched, thick-walled, pale brown to medium brown, micro- to macronematous, septate, constricted at septa, subcylindrical, straight to geniculate, or irregularly curved. *Conidiogenous cells* terminal, intercalary or lateral, straight, darkened, thickened, subcylindrical. *Conidia* solitary or in chains, subglobose to irregularly-shaped, pale brown, smooth- and thick-walled, 0–1 septate. **Sexual morph** not observed.

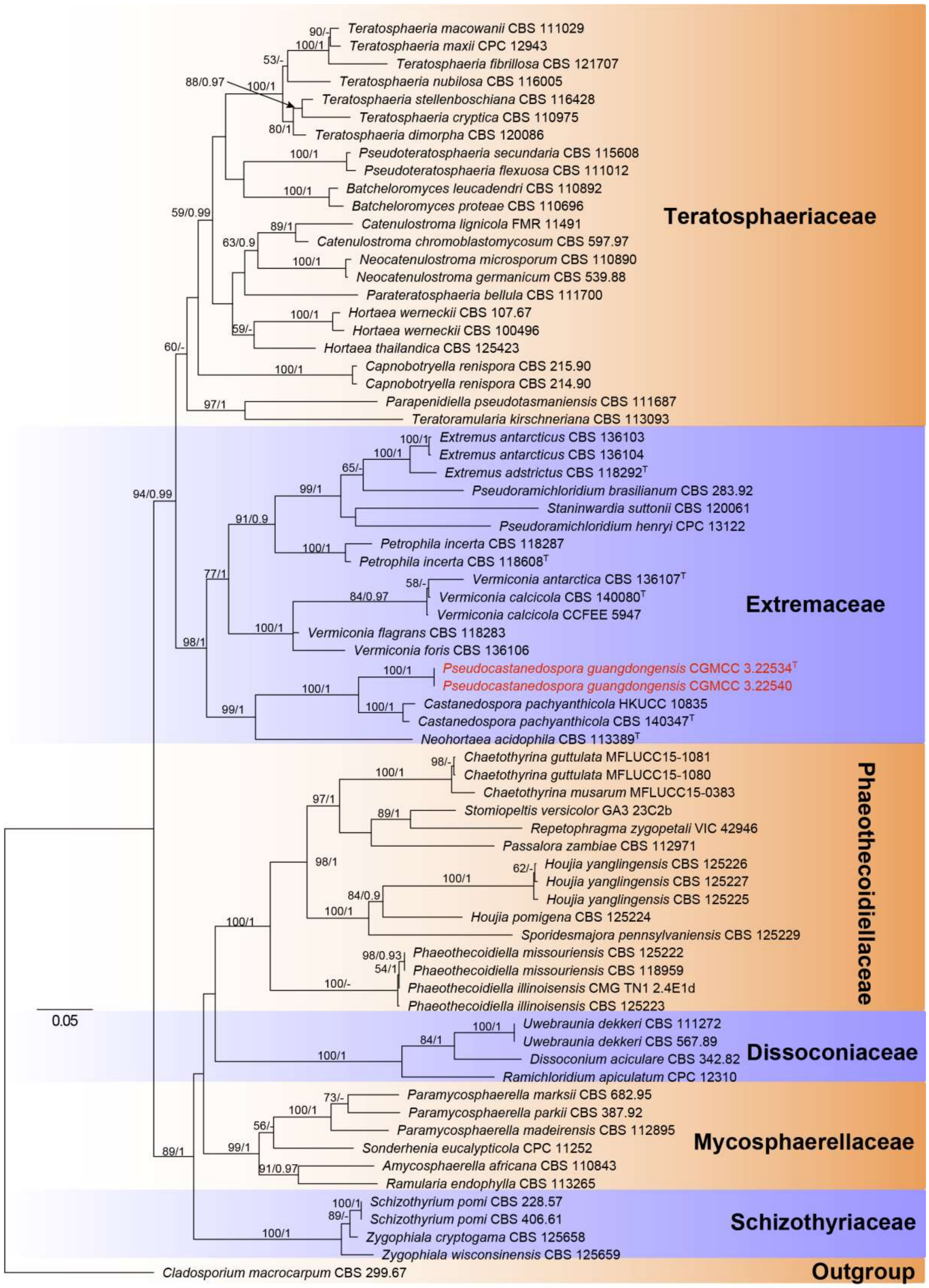

Fig. SD-1 Maximum likelihood tree of *Extremaceae* and allied families based on ITS and LSU sequences. The RAxML bootstrap support values (BS) above 50% and Bayesian posterior probabilities (BPP) above 0.90 are presented at the nodes BS/BPP. The tree was rooted to *Cladosporium macrocarpum* CBS 299.67. Ex-type cultures are indicated with a letter "T" after the accession number. The new species are printed in red font.

Type: Pseudocastanedospora guangdongensis M. Li, M. Raza & L. Cai

Notes: *Pseudocastanedospora* is herein introduced to accommodate *P. guangdongensis*. Phylogenetically, two strains representing *P. guangdongensis* clustered in an independent clade sister to *Castanedospora* (Fig. SD-1). Morphologically, two genera are different in the shape of conidiophores, conidiogenous cells and conidia (smooth conidiophores, subcylindrical conidiogenous cells and 0–1 septate conidia in *Pseudocastanedospora*; verrucose conidiophores doliiform or cylindrical conidiogenous cells and 31-200 septate conidiain *Castanedospora*).

Pseudocastanedospora guangdongensis M. Li, M. Raza & L. Cai, ***sp. nov.***

Fungal Names: FN571496; Fig. SD-2

*Etymology:*Named after the type locality , Guangdong province.

Hyphae septate, branched, verruculose, thick-walled, hyaline to pale brown, 1.5–3.0 µm wide. **Asexual morph** *Conidiophores* solitary, terminal, lateral or intercalary on the hyphae, frequently branched, thick-walled, pale to medium brown, micro- to macronematous, 1–7 septate, constricted at septa, subcylindrical, straight to geniculate, or irregularly curved, 7.5–62 × 2.0–3.5 µm. *Conidiogenous cells* terminal, intercalary or lateral, straight, darkened, thickened, subcylindrical, 4.0–9.0 × 2.0–3.0 µm. *Conidia* solitary or in long chains up to 8 units, subglobose to irregularly-shaped, 1–2 flat tipped loci that can be subdenticulate, pale brown, smooth- and thick-walled, 0–1 septate, 3.0–10 × 2.0–3.5 µm (av. = 5.2 ± 1.85 × 2.8 ± 0.36 µm, n = 30). **Sexual morph** not observed.

Culture characteristics—Colonies on PDA attaining 6–7 mm diam. after 4 weeks, felty, raised at center, margin entire, olive to black, aerial mycelia sparse. Reverse black. Colonies on MEA attaining 10–12 mm diam. after 4 weeks, flat, felty to pulverulent, margin slightly undulate, olive to black, aerial mycelia sparse. Reverse dark brown to black. Sporulation within 4 weeks on MEA. Colonies on OA attaining 10–11 mm diam. after 4 weeks, flat, felty, margin entire, black, aerial mycelia sparse. Reverse black.

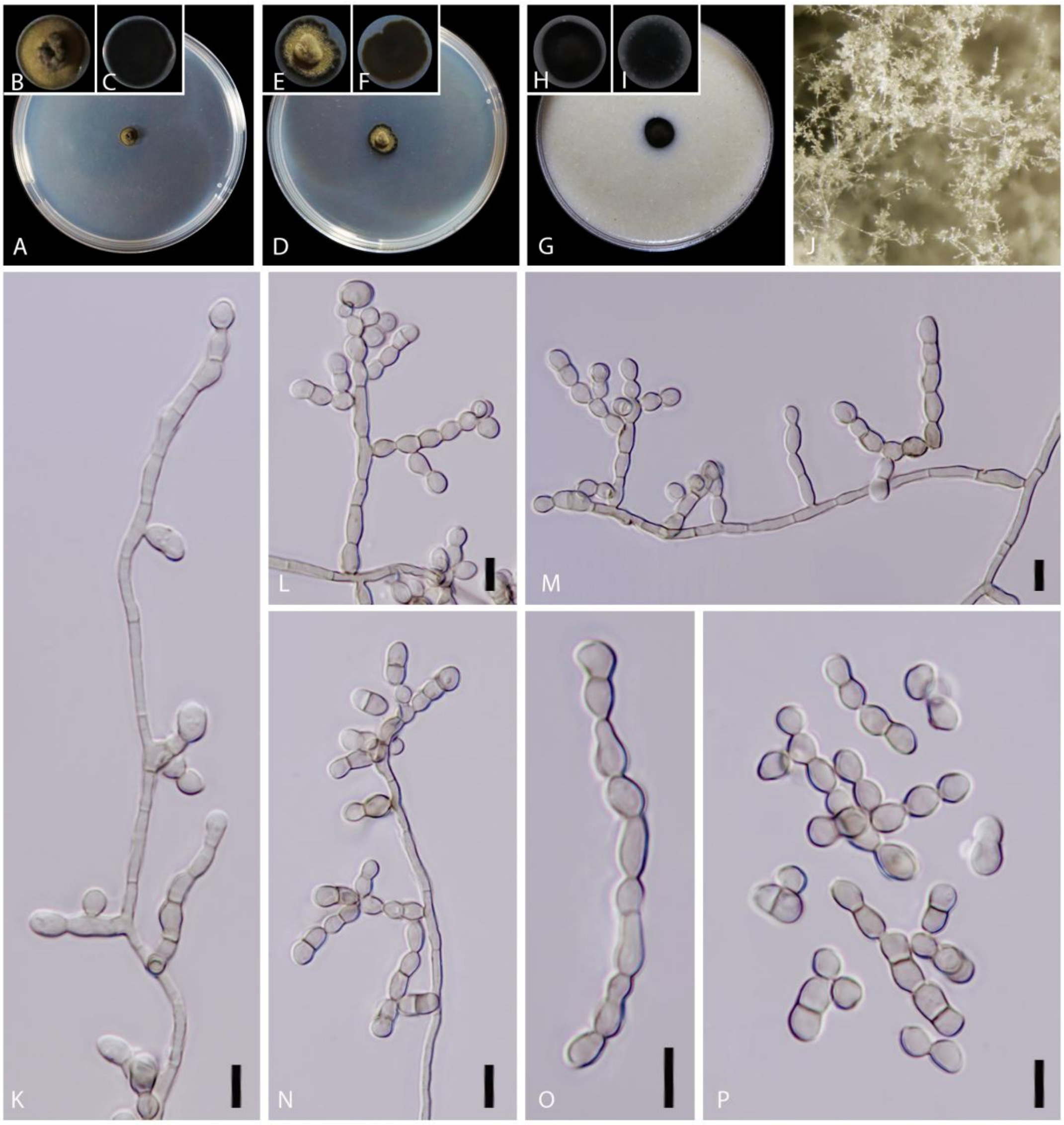

Fig. SD-2 ***Pseudocastanedospora guangdongensis*** **(from ex-holotype CGMCC 3.22534)**. **A–I** Surface and reverse of colony on PDA, MEA, and OA. **J**, Conidiomata on MEA. **K–N** Conidiophores and conidiogenous cells. **O–P** Conidia. Scale bars: **K–P** = 5 μm.

Material examined: CHINA, Guangdong, National Mangrove Nature Reserve of Zhanjiang, isolated from sediment, November 2019, M. Li and J. E. Huang, HMAS 352454 (holotype designated here, dried culture), ex-type living culture CGMCC 3.22534 = LC15942; ibid., CGMCC 3.22540 = LC15943.

Notes: *Pseudocastanedospora guangdongensis* is currently the only species in the genus *Pseudocastanedospora*. Based on the multi-locus phylogenetic analyses, our new species belong to *Extremaceae*. (Fig. SD-1). Two strains representing *Pseudocastanedospora guangdongensis* formed an independent clade, clearly

separated from species in *Castanedospora* (Delgado et al. 2018). Morphologically, *P. guangdongensis* can be distinguished from *C. pachyanthicola* in having smooth conidiophores and obviously constricted septa in conidiophores. Furthermore, the conidiophores are much narrower than that of *C. pachyanthicola* (2.0–3.5 µm *vs*. 3–6 µm). Additionally, the shape and size of conidiogenous cells are distinguishable (subcylindrical, 4–9 × 2.0–3.0 µm in *P. guangdongensis*; doliiform or cylindrical, 5–8 × 3–4 µm in *C. pachyanthicola*). Moreover, conidia in *P. guangdongensis* are solitary or 0–1 septate in chains, while in *C. pachyanthicola* conidia are solitary and 31–200 septate. Also, their conidial length are much different (5–8 µm in *P. guangdongensis*; 172–825 µm in *C. pachyanthicola*).

Subclass Pleosporomycetidae C.L. Schoch, Spatafora, Crous & Shoemaker, Mycologia 98(6): 1048 (2007) [2006]

Pleosporales Luttr. ex M.E. Barr, Prodr. Cl. Loculoasc. (Amherst): 67 (1987)

Macrodiplodiopsidaceae Voglmayr, Jaklitsch & Crous, IMA Fungus 6(1): 178 (2015)

Macrodiplodiopsidaceae was introduced by Crous et al. (2015) to accommodate *Macrodiplodiopsis* and *Pseudochaetosphaeronema* in the suborder Massarineae. Subsequently, Tanaka et al. (2015) and Ariyawansa et al. (2015) placed the genera *Camarographium* and *Pseudomonodictys* in *Macrodiplodiopsidaceae*. Morphologically, *Macrodiplodiopsidaceae* is characterized by dark brown, obovoid, straight to inequilateral, asymmetric, eu- and distoseptate ascospores, and the asexual morph is globose to collabent conidiomata and ellipsoid to obovoid or clavate conidia (Crous et al. 2015).

Pseudochaetosphaeronema Punith., Nova Hedwigia 31(1-3): 126 (1979)

The genus *Pseudochaetosphaeronema*, described based on asexual morphology, was introduced by Punithalingam (1979), and its sexual morph was recently found and described by Boonmee et al. (2021). The asexual morphology of *Pseudochaetosphaeronema* is characterized by black obpyriform pycnidia with a long neck, hyaline and phialidic conidiophores, and unicellular subspherical to ellipsoidal

conidia. While the sexual morphology of *Pseudochaetosphaeronema* is characterized by uni-loculate, black, globose to subglobose ascomata, 8-spored and bitunicate asci, and hyaline, fusiform and 1-septate ascospores. Here, we introduce a new species *P. irregulare* isolated from mangrove sediment through *in situ* cultivation (Fig. SD-3).

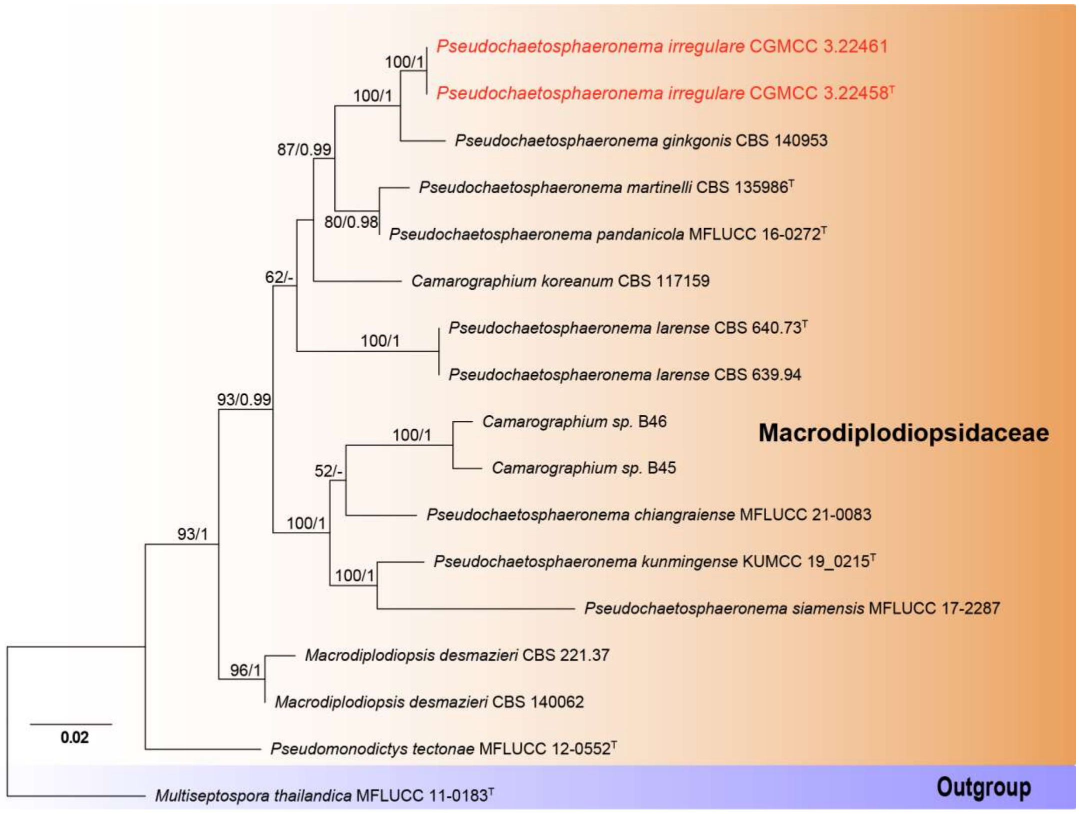

Fig. SD-3 Maximum likelihood tree of *Macrodiplodiopsidaceae* based on ITS, LSU, SSU, *tef1* and *rpb2* sequences. The RAxML BS above 50% and BPP above 0.90 are presented at the nodes BS/BPP. The tree was rooted to *Multiseptospora thailandica* MFLUCC 11-0183. Ex-type cultures are indicated with a letter "T" after the accession number. The new species are printed in red font.

Pseudochaetosphaeronema irregulare M. Li & L. Cai, ***sp. nov.***

Fungal Names: FN571497; Fig. SD-4

Etymology: Referring to the irregular shape of its pycnidia.

Hyphae hyaline, septate, rough, branched, 1.4–2.0 μm wide. **Asexual morph** *Conidiomata* pycnidial, superficial or semi-immersed, scattered and solitary, globose to subglobose, hemisphaerical to subconical, lageniform to irregularly-shaped with age, dark brown to black, covered with abundant mycelial outgrowths, 180–430 × 220–470 μm; non-papillate or with up to one papillate ostiole; pycnidial wall

pseudoparenchymatous, 30–85 µm thick, outer layers composed of brown to dark brown cells of 25–49 µm diam, flattened at the base. *Conidiophores* reduced to conidiogenous cells. *Conidiogenous cells* enteroblastic, phialidic, hyaline, smooth, unbranched, cylindrical to cylindric-clavate, 6.5–14.0 × 1.0–2.0 µm. *Conidia* ellipsoidal, oval, spherical, thin- and smooth-walled, hyaline, aseptate, 2.0–4.0 × 1.0–2.0 µm (av. = 3.1 ± 0.39 × 1.6 ± 0.14 µm, n = 50). *Conidial matrix* hyaline. **Sexual morph** not observed.

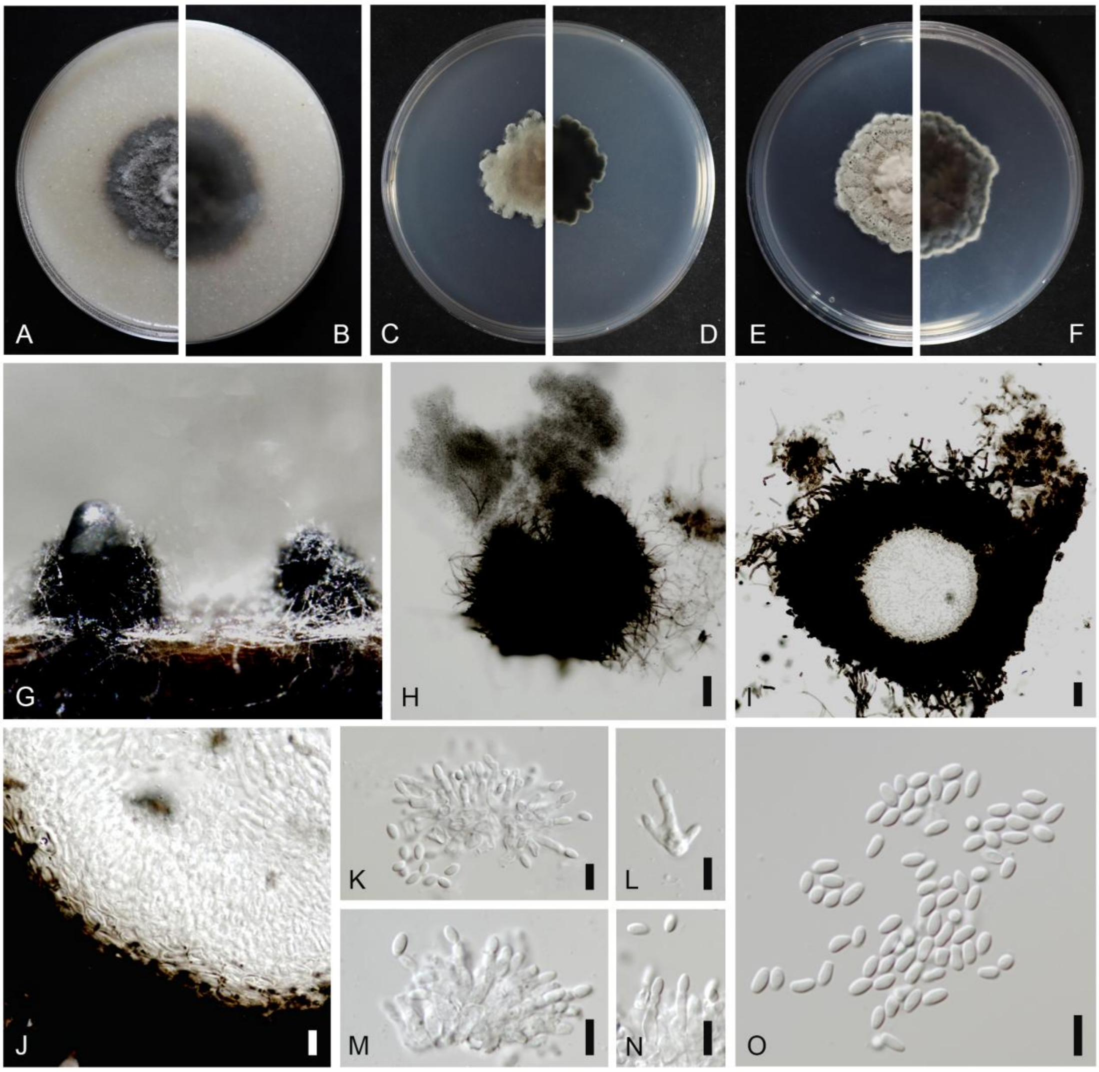

Fig. SD-4 *Pseudochaetosphaeronema irregulare* (from ex-holotype CGMCC 3.22458). **A–F** Surface and reverse of colony on OA, MEA and PDA. **G** Pycnidia forming on SNA. **H** Pycnidium. **I** Section through pycnidium. **J** Section of pycnidial wall. **K–N** Conidophores, conidiogenous cells and conidia. **O** Conidia. Scale bars: **H** = 50 µm, **I** = 20 µm, **J–O**=5 µm.

Culture characteristics—Colonies after 4 weeks at 25 °C, on OA reaching 39–41

mm diam, flat, floccose, margin irregular, grey to black, aerial mycelia abundant. Reverse brown to black. On MEA reaching 30–32 mm diam, flat, felty, margin irregular, bracken green to brown, aerial mycelium moderate abundant. Reverse black with white edge. On PDA reaching 38–40 mm diam, flat, felty, plicated, effuse, margin fimbriate, grey to pale grown, aerial mycelium sparse. Reverse black with white edge. NaOH spot test: black discolouration on MEA plate.

Material examined: CHINA, Guangdong, National Mangrove Nature Reserve of Futian Shenzhen, isolated from sediment, September 2020, M. Li and Z. F. Zhang, HMAS 352445 (holotype designated here, dried culture), ex-type living culture CGMCC 3.22458 = LC15924; ibid., CGMCC 3.22461 = LC15925.

Notes: Based on the multi-locus phylogenetic analyses, *Pseudochaetosphaeronema irregulare* forms an independent clade clearly separated from *P. ginkgonis* (Fig. SD-3). *P. irregulare* shares low sequence similarity with *P. ginkgonis* (95.2% similarity, 23 base pairs (bp) difference in 475 bp of ITS; 99.2% similarity, 7 bp difference in 845 bp of LSU; 99.7% similarity, 3 base pairs (bp) difference in 1026 bp of SSU; 97.8% similarity, 17 base pairs (bp) difference in 777 bp of *tef1*). Morphologically, *P. subconicum* is characterised by subconical, large and thick-walled pycnidia densely covered by long hairs, and ostioles with up to one papilla with hyaline conidial matrix.

Roussoellaceae Jian K. Liu, Phook., D.Q. Dai & K.D. Hyde, Phytotaxa 181(1): 7 (2014)

Roussoellaceae was established by Liu et al. (2014) to accommodate *Neoroussoella*, *Roussoella* and *Roussoellopsis.* However, the phylogenetic relationship among three genera remained unresolved. Subsequently, the family has been treated as a synonym of *Thyridariaceae* (Jaklitsch and Voglmayr 2016). However, based on increased taxon sampling in the phylogenetic analysis, *Roussoellaceae* appeared to be a well-resolved clade in *Pleosporales,* and several more genera *Thyridaria*, *Parathyridaria*, *Cycasicola*, *Pararoussoella*, *Pseudoneoconiothyrium*, and *Pseudoroussoella* were included in the family

(Tibpromma et al. 2018; Wanasinghe et al. 2018; Jiang et al. 2019; Karunarathna et al. 2019; Phookamsak et al. 2019; Mapook et al. 2020). In this study, we provide an updated phylogenetic analysis for *Roussoellaceae* based on LSU, SSU, ITS, *rpb2*, and *tef1* sequences, and we describe a new genus *Nothoroussoella* and a new species *N. lageniformis* (Fig. SD-5).

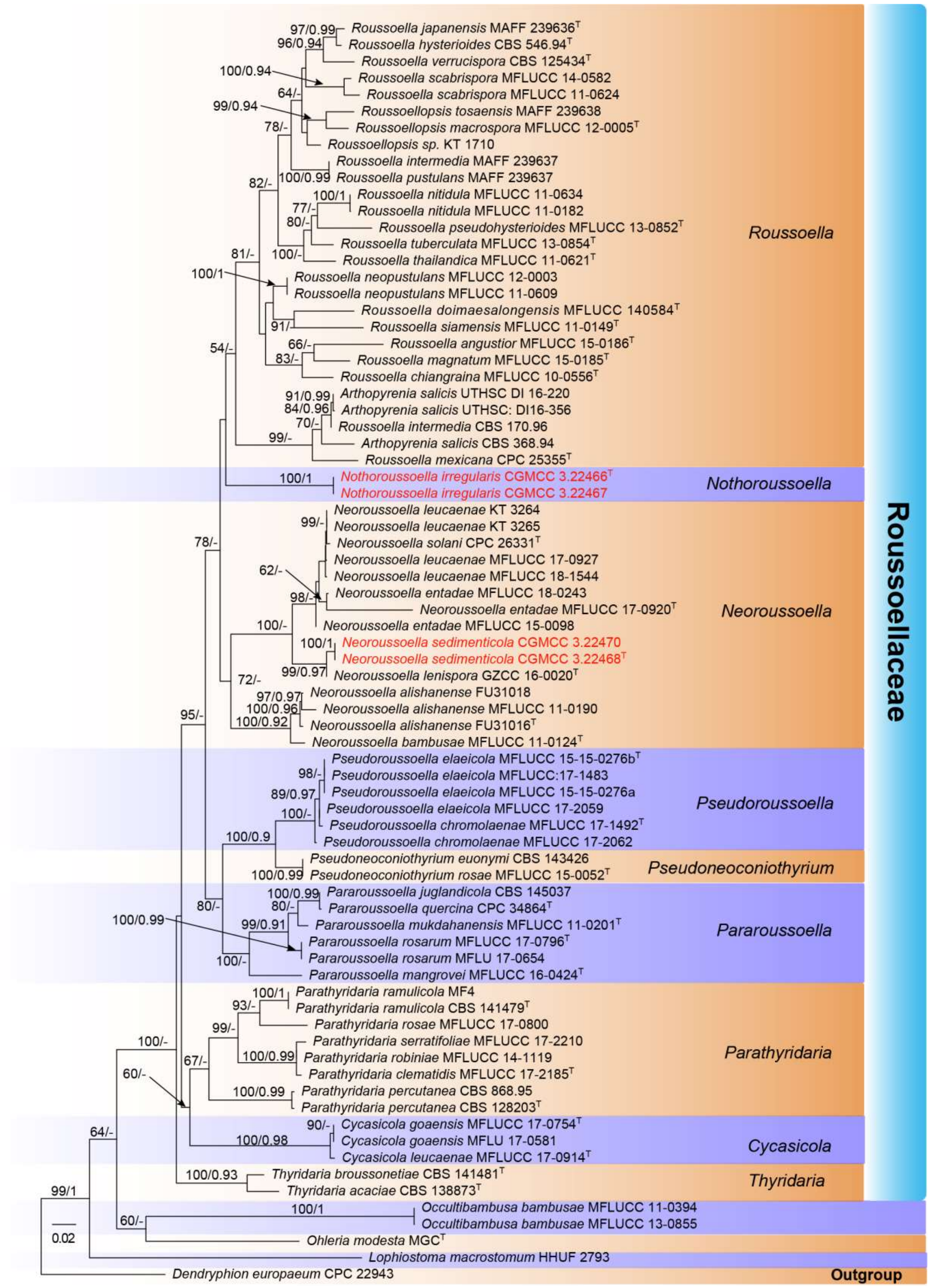

Fig. SD-5 Maximum likelihood tree of *Roussoellaceae* based on ITS, LSU, SSU, *rpb2* and *tef1*

sequences.The RAxML BS above 50% and BPP above 0.90 are presented at the nodes BS/BPP. The tree was rooted to *Dendryphion europaeum* CPC 22943. Ex-type cultures are indicated with a letter "T" after the accession number. The new species are printed in red font.

Neoroussoella Jian K. Liu, Phook. & K.D. Hyde, Phytotaxa 181(1): 21 (2014)

The genus *Neoroussoella* was introduced with a single species *N. bambusae* by Liu et al. (2014). It is characterized by asexual morph producing relatively smaller, hyaline and smooth-walled conidia. Our multi-locus phylogenetic analysis (Fig. SD-5) reveals one new species *N. sedimenticola* from mangrove sediments.

Neoroussoella sedimenticola M. Li & L. Cai, ***sp. nov.***

Fungal Names: FN571498; Fig. SD-6

Etymology: Referring to the substrate in which this fungus was isolated.

Hyphae hyaline, septate, smooth, branched, 1.0–2.0 µm wide. **Asexual morph** *Conidiomata* pycnidial, superficial or semi-immersed, scattered or aggregated, mostly confluent, sometimes solitary, globose, subglobose, lageniform to irregularly-shaped with age, brown to dark brown, covered by hyphal outgrowths, 100–260 × 100–270 µm; non-papillate or with up to one papillate ostiole; pycnidial wall pseudoparenchymatous, 3–5 layers, 35–50 µm thick, outer layers composed of brown to dark brown cells of 18–40 µm diam. *Conidial matrix* black. *Conidiophores* hyaline, branched, usually reduced to conidiogenous cells. *Conidiogenous cells* phialidic, hyaline, smooth, globose, subglobose, ampulliform or lageniform, 5.0–7.0 × 3.5–4.5 µm. *Conidia* ellipsoidal, oval, thin- and smooth-walled, hyaline, aseptate, 2.0–4.0 × 1.5–3.0 µm (av. = 3.3 ± 0.33 × 2.1 ± 0.25 µm, n = 50), 1–2-guttulate. **Sexual morph** not observed.

Culture characteristics—Colonies after 3 weeks at 25 °C, on OA reaching 42–44 mm diam, flat, felty to pulverulent, margin entire, white to isabelline, aerial mycelium sparse. Reverse seagreen near the centre, pale brown with white edge. On MEA reaching 25–27 mm diam, flat, coriarious, margin undulate, white to olive, aerial mycelium extremely sparse. Reverse white to olive. On PDA reaching 32–34 mm diam, flat, felty to pulverulent, plicated at edge, margin fimbriate, white to bisque,

aerial mycelium sparse. Reverse bisque to yellowish brown. NaOH spot test negative on MEA.

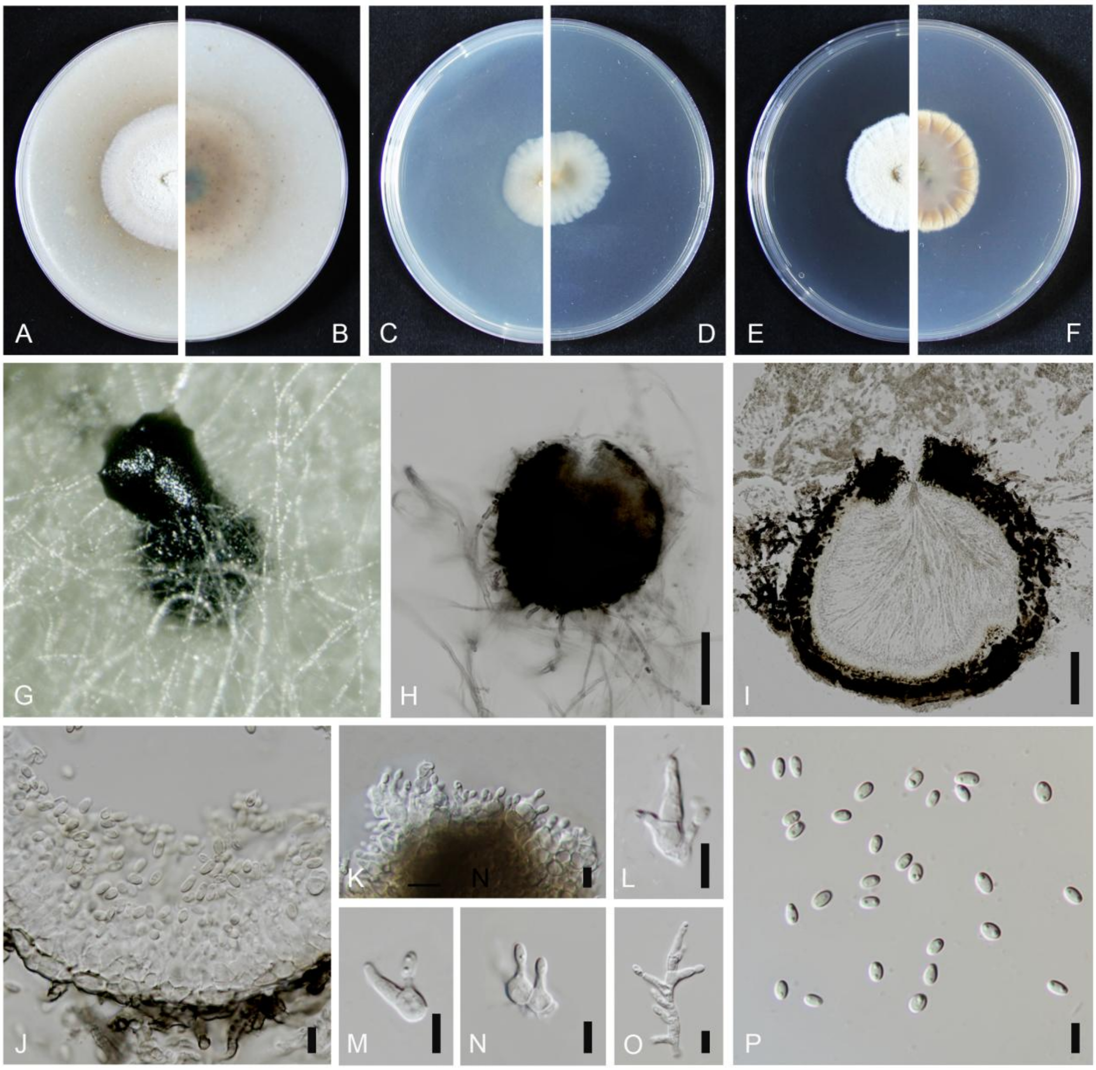

Fig. SD-6 ***Neoroussoella sedimenticola*** **(from ex-holotype CGMCC 3.22468)**. **A–F** Surface and reverse of colony on OA, MEA and PDA **G** Pycnidia forming on SNA. **H** Pycnidium. **I** Section through pycnidium. **J** Section of pycnidial wall. **K–O** Coniodiphores, conidiogenous cells and conidia. **P** Conidia. Scale bars: **H–I** = 50 µm, **J–P**=5 µm.

Material examined: CHINA, Guangdong, National Mangrove Nature Reserve of Futian Shenzhen, isolated from sediment, September 2020, M. Li and Z. F. Zhang, HMAS 352447 (holotype designated here, dried culture), ex-type living culture CGMCC 3.22468 = LC15928; ibid., CGMCC 3.22470 = LC15929.

Notes: Based on the multi-locus phylogeny, *Neoroussoella sedimenticola* forms an independent clade sister to *N. lenispora* (Fig. SD-5). *N. sedimenticola* only

produced asexual morph while in contrast *N. lenispora* only has sexual morph. Also, the size of conidiogenous cells of *N. sedimenticola* is larger than all known species in *Neoroussoella* (Liu et al. 2014; Jayasiri et al. 2019; Phukhamsakda et al. 2020).

Nothoroussoella M. Li & L. Cai, ***gen. nov.***

Fungal Names: FN571499

Etymology: Referring to the close phylogenetic relationship with the genus *Roussoella.*

Asexual morph *Conidiomata* pycnidial, semi-immersed, scattered or aggregated, globose to subglobose, oval to irregularly-shaped when confluent, brown to dark brown, unilocular, covered by abundant long hyphal outgrowths; with one papillate ostiole, sometimes elongated to a short neck; pycnidial wall pseudoparenchymatous, multi-layered, outer layers composed of brown, flattened, polygonal cells. *Conidiogenous cells* phialidic, hyaline to light yellow, smooth, ampulliform, lageniform or subconical. *Conidia* ellipsoidal to oblong, thin- and smooth-walled, hyaline becoming pale brown, aseptate, guttulate. **Sexual morph** not observed.

Type: Nothoroussoella irregularis M. Li & L. Cai

Notes: *Nothoroussoella* is herein introduced to accommodate the new species *N. lageniformis*. Two trains clustered together in an independent clade closely related to *Roussoella* (Fig. SD-5). Morphologically, *Nothoroussoella* is characterized by unilocular pycnidium, wheras *Roussoella* produces multilocular pycnidium (Liu et al. 2014).

Nothoroussoella irregularis M. Li & L. Cai, ***sp. nov.***

Fungal Names: FN571500; Fig. SD-7

Etymology: Referring to the irregular shape of its conidiogenous cells.

Hyphae hyaline, septate, smooth, branched, 1.0–2.0 µm wide. **Asexual morph** *Conidiomata* pycnidial, semi-immersed, scattered or aggregated, globose, subglobose, oval to irregularly-shaped when confluent, brown to dark brown, unilocular, covered by abundant long hyphal outgrowths, 330–610 × 260–570 µm; with one papillate ostiole, sometimes elongated to a short neck; pycnidial wall pseudoparenchymatous,

5–8 layers, 20–30 µm thick, outer layers composed of brown, flattened, polygonal cells of 12–21 µm diam. *Conidiogenous cells* phialidic, hyaline to light yellow, smooth, ampulliform, lageniform or subconical, 3.5–9.5 × 2.0–4.5 µm. *Conidia* ellipsoidal to oblong, thin- and smooth-walled, hyaline, becoming pale brown with time, aseptate, 2.5–4.5 × 1.0–2.0 µm (av. = 3.9 ± 0.37 × 1.7 ± 0.2 µm, n = 30), guttulate. *Conidial matrix* black. **Sexual morph** not observed.

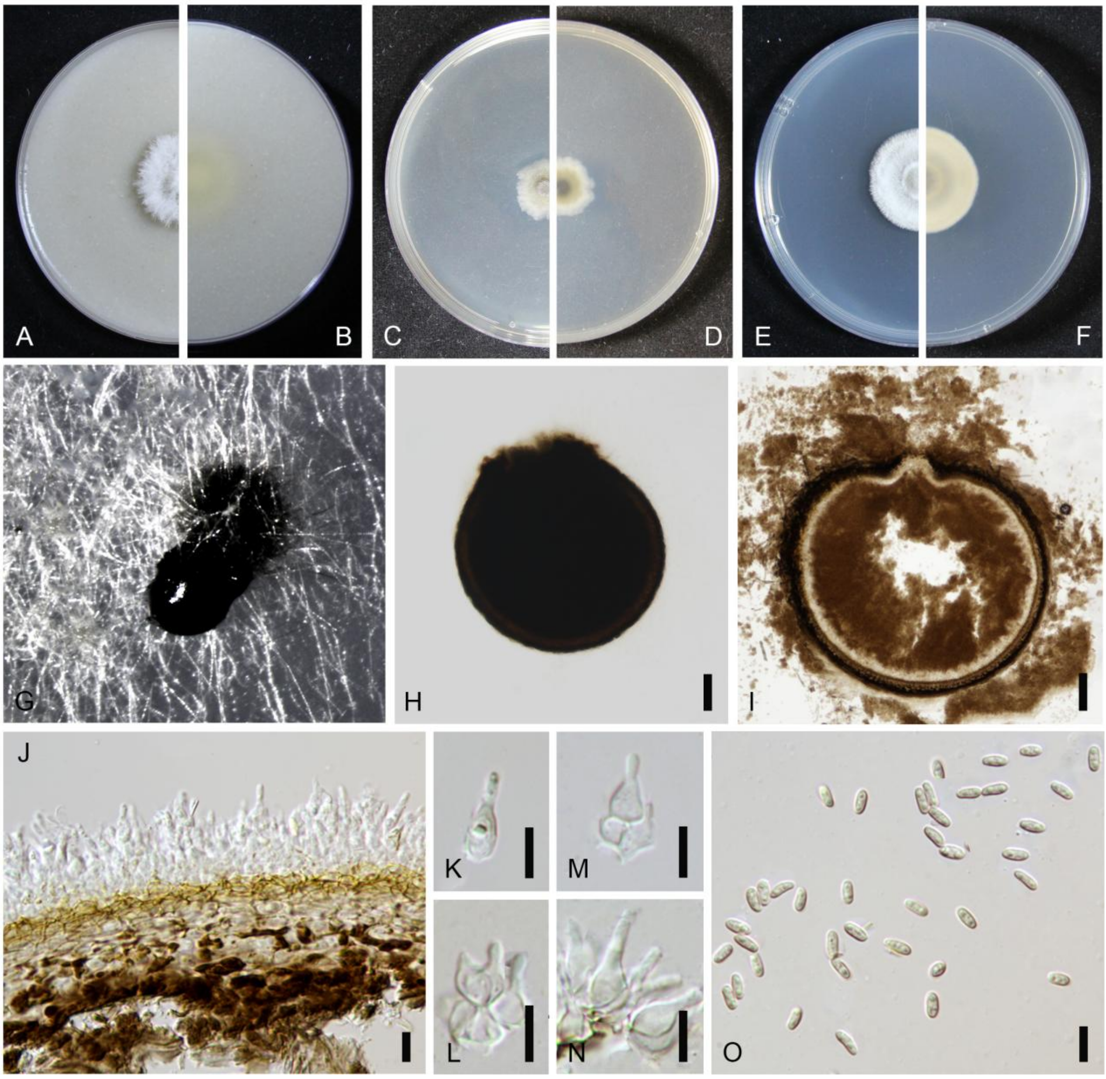

Fig. SD-7 ***Nothoroussoella irregularis*** **(from ex-holotype CGMCC 3.22466)**. **A–F** Surface and reverse of colony on OA, MEA and PDA **G** Pycnidia forming on SNA. **H** Pycnidium. **I** Section through pycnidium. **J** Section of pycnidial wall. **K–N** Conidiogenous cells. **O** Conidia. Scale bars: **H–I** = 50 µm, **J–O**=5 µm.

Culture characteristics—Colonies after 2 weeks at 25 °C, on OA reaching 27–29 mm diam, floccose, raised at center, margin fimbriate, white, aerial mycelium

abundant. Reverse white to pale yellow. On MEA reaching 16–18 mm diam, floccose, raised at center, margin irregular, white to olive, aerial mycelium sparse. Reverse black near the centre, olive with white edge. On PDA reaching 24–29 mm diam, floccose, raised at center, annular, margin fimbriate, white to greyish white, aerial mycelium abundant. Reverse white to greyish white. NaOH spot test negative on MEA.

Material examined: CHINA, Guangdong, National Mangrove Nature Reserve of Futian Shenzhen, isolated from sediment, September 2020, M. Li and Z. F. Zhang, HMAS 352446 (holotype designated here, dried culture), ex-type living culture CGMCC 3.22466 = LC15926; ibid., CGMCC 3.22467 = LC15927.

Notes: Phylogenetic analyses based on ITS, LSU, SSU, *tef1* and *rpb2* sequences showed that our new species should be classified in *Roussoellaceae*. (Fig. SD-5). Two strains representing *Nothoroussoella irregularis* clustered in well-supported clade and closely related to *Roussoella*. Morphologically, *N. irregularis* is characterised by unilocular pycnidia with a short neck and a distinct ostiole with one papilla, while *Roussoella* usually produces multilocular pycnidia without ostiole or has indistinct ostioles (Liu et al. 2014).

Class Eurotiomycetes O.E. Erikss & Winka, Myconet 1(1): 6 (1997)

Subclass Eurotiomycetidae Tehler, Cladistics 8: 236 (1988)

Eurotiales G.W. Martin ex Benny & Kimbr., Mycotaxon 12(1): 23 (1980)

Aspergillaceae Link, Abh. dt. Akad. Wiss. Berlin: 165 (1826) [1824]

Aspergillaceae was first established by Link (1826) and re-instated by Houbraken and Samson (2011) based on multi-locus phylogeny. Species in *Aspergillaceae* are mainly saprophytic and usually occur in soil, and some of them are known to have positive or negative effects on human activities (Houbraken et al. 2014).

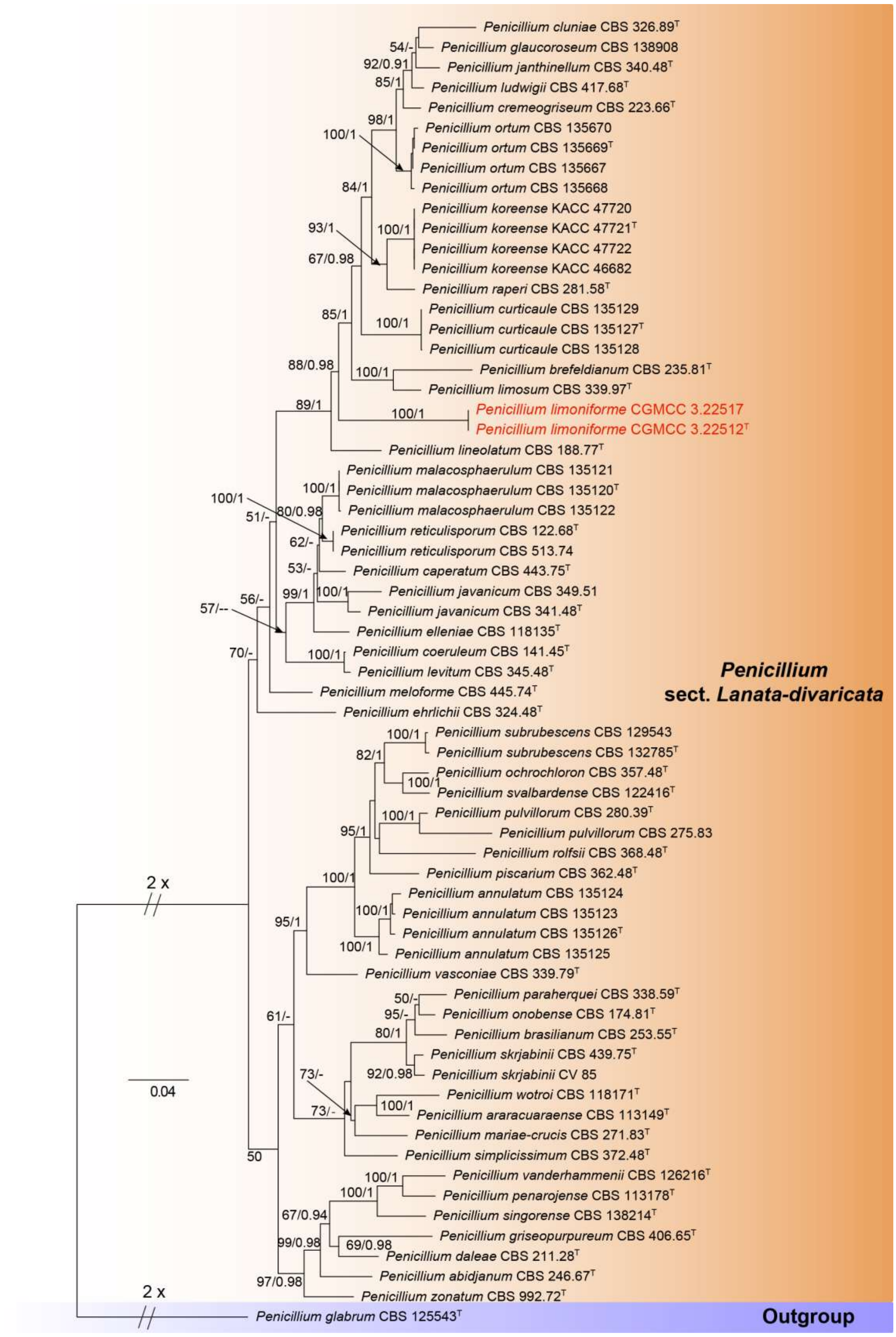

Fig. SD-8 Maximum likelihood tree of *Penicillium* sect. *Lanata-divaricata* based on ITS, *cam*, *tub2* and *rpb2* sequences. The RAxML BS above 50% and BPP above 0.90 are presented at the nodes BS/BPP. The tree was rooted to *Penicillium glabrum* CBS 125543. Ex-type cultures are indicated with a letter "T" after the accession number. The new species are printed in red font.

Penicillium Link, Mag. Gesell. naturf. Freunde, Berlin 3(1–2): 16 (1809)

Penicillium is widely known for the production of the antibiotic penicillin by several species. Currently, two subgenera and 26 sections were proposed for *Penicillium* (Houbraken et al. 2020). In this study, two new species are described as *P. limoniforme* and *P. sedimenticola* (Figs. SD-8–11).

Penicillium limoniforme M. Li & L. Cai, ***sp. nov.***

Fungal Names: FN571501; Fig. SD-9

Etymology: Referring to the shape of its limoniform conidia.

Hyphae hyaline, septate, smooth, branched, 1.0–2.0 µm wide. *Synnemata* formed after 7 days on CAMSW up to 40–90 µm long. **Asexual morph** *Conidiophores* born from aerial hyphae, monoverticillate. *Stipes* smooth walled 27–63 × 1.5–2.0 µm. *Phialides* in verticils commonly 2–5, ampulliform, 6.5–10 × 1.5–2.0 µm. *Conidia* limoniform, fusiform to oval, 2.5–3.0 × 1.5–2.0 µm (av. = 2.7 ± 0.21 × 1.6 ± 0.14 µm, n = 50), with smooth walls, in irregular columns; *Sclerotia* not produced. **Sexual morph** not observed.

Culture characteristics—Colony diam: after 7 days at 25°C: on PDA, 19–20 mm; on YES, 21–23 mm; on MEA, 21–22 mm; on CAMSW, 25–29 mm. Colony characteristics: On PDA 25°C, 7 days: Colonies moderately deep, deeply sulcate and wrinkled; margins irregular; mycelia white; texture velutinous; sporulation absent; exudate absent; soluble pigment absent; reverse pale white. On YES 25°C, 7 days: Colonies moderately deep, radially, sulcate; margins moderately wide, entire; mycelia white; texture velutinous; sporulation absent; exudate absent; soluble pigment absent; reverse pale white to light yellow. On MEA at 25°C, after 7 days: Colonies flat, radially; margins low, narrow, entire; texture floccose; sporulation absent; exudate absent; soluble pigment absent; reverse pale white. On CAMSW at 25°C, after 7 days: Colonies flat, radially; margins low, narrow, entire; texture floccose; sporulation sparse, conidia *en masse* white; soluble pigments absent; exudates absent, reverse pale white.

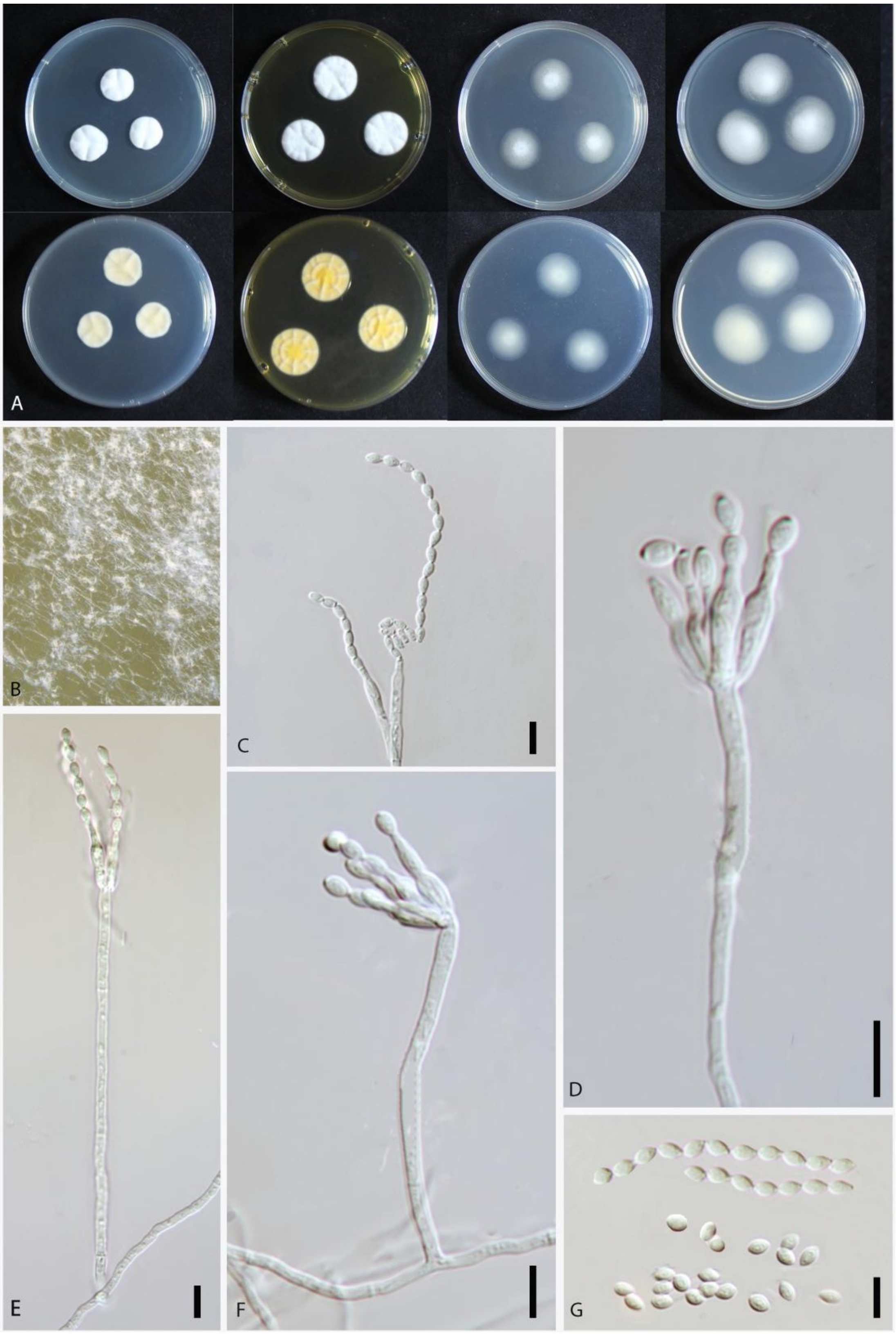

Fig. SD-9 ***Penicillium limoniforme*** **(from ex-holotype CGMCC 3.22512)**. **A** Surface and reverse of colony on PDA, YES, MEA and CAMSW. **B** Sporulation on CAMSW. **C–F** Conidiophores and conidiogenous cells. **G** Conidia. Scale bars: **C–G** = 5 µm.

Material examined: CHINA, Guangdong, National Mangrove Nature Reserve of Zhanjiang, isolated from sediment, November 2019, M. Li and J. E. Huang, HMAS 352451 (holotype designated here, dried culture), ex-type living culture CGMCC 3.22512 = LC15936; ibid., CGMCC 3.22517 = LC15937.

Notes: *Penicillium limoniformi* forms a distinct clade in *Penicillium* section *Lanata-divaricata* (Fig. SD-8), closely related to *P. janthinellum*, *P. limosum* and *P. raperi*. Morphologically, the conidia of *P. limoniforme* is thinner than *P. janthinellum* (2.5–3.0 µm *vs*. 3.0–3.5 µm) (Biourge 1923). Meanwhile, conidia of *P. limoniforme* are limoniform, fusiform to oval, while which are globose to subglobose in *P. limosum* (Ueda 1995). In addition, conidia of *P. limoniforme* are much smaller than *P. raperi* (2.5–3.0 × 1.5–2.0 µm *vs*. 3–3.5 × 2.4–2.8 µm) (Smith 1957).

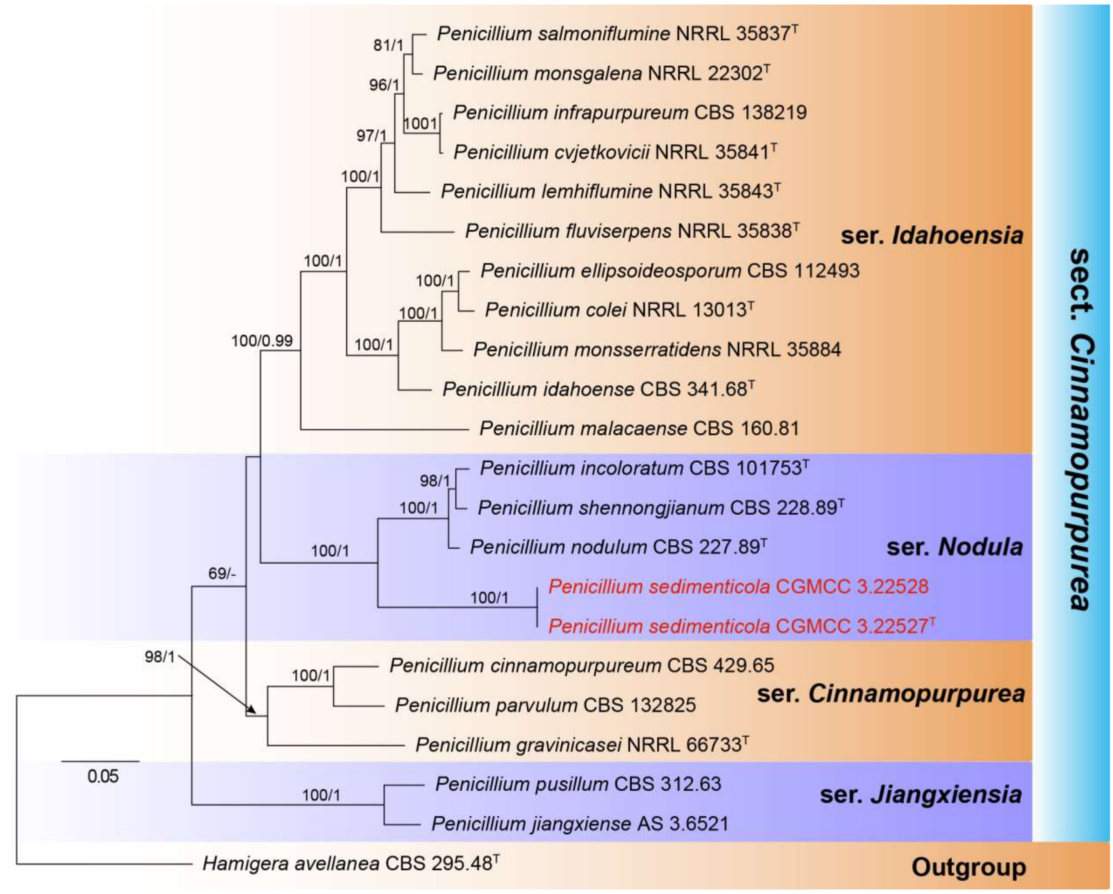

Fig. SD-10 Maximum likelihood tree of *Penicillium* sect. *Cinnamopurpurea* based on ITS, *cam*, *tub2* and *rpb2* sequences. The RAxML BS above 50% and BPP above 0.90 are presented at the nodes BS/BPP. The tree was rooted to *Hamigera avellanea* CBS 295.48. Ex-type cultures are indicated with a letter "T" after the accession number. The new species are printed in red font.

Penicillium sedimenticola M. Li & L. Cai, ***sp. nov.***

Fungal Names: FN571502; Fig. SD-11

Etymology: Referring to the substrate in which this fungus was isolated.

Hyphae hyaline, septate, smooth, branched, 1.0–2.0 µm wide. *Synnemata* formed after 14 days on MEA up to 20–80 µm long. **Asexual morph** *Conidiophores* born from aerial hyphae, monoverticillate, apically swollen 2–7 µm diam. *Stipes* smooth walled 9.0–54 × 1.5–2.0 µm. *Phialides* in verticils commonly 2–4, ampulliform, 5.0–7.0 × 1.5–2.5 µm with short collula. *Conidia* spherical to ellipsoidal, 1.5–2.5 × 1.5–2.0 µm (av. = 2.0 ± 0.19 × 1.6 ± 0.23 µm, n = 50), with smooth walls, in irregular chains. **Sexual morph** not observed.

Culture characteristics—Colony diameter, after 14-day growth: MEA 37°C 10–12 mm; incubated at 25°C:on PDA, 12–15 mm; on CYA, 10–12 mm; on MEA, 15–17 mm. Colony characteristics: On PDA 25°C, 14 days: Colonies composed of densely felted hyphae, sulcate and protuberant incenters, smooth surfaced; margins entire; mycelia white; texture velutinous; sporulation absent;exudate absent; soluble pigment absent; reverse pale white. On CYA 25°C, 14 days: Colonies moderately deep, radially, sulcate, and protuberant incenters; margins low, moderately wide, irregular; mycelia white; texture velutinous; sporulation absent; exudate absent; soluble pigment absent; reverse pale white to mars yellow. On MEA at 25°C, after 14 days: Colonies moderately deep, radially, and protuberant incenters; margins low, moderately wide, entire; texture floccose; sporulating well, in color pale white; soluble pigments absent; exudates absent, reverse pale white.On MEA at 37°C, after 14 days: Colonies flat, radially, and protuberant incenters; margins wide, entire; texture floccose; sporulation absent; soluble pigments absent; exudates absent, reverse pale white to pale yellow.

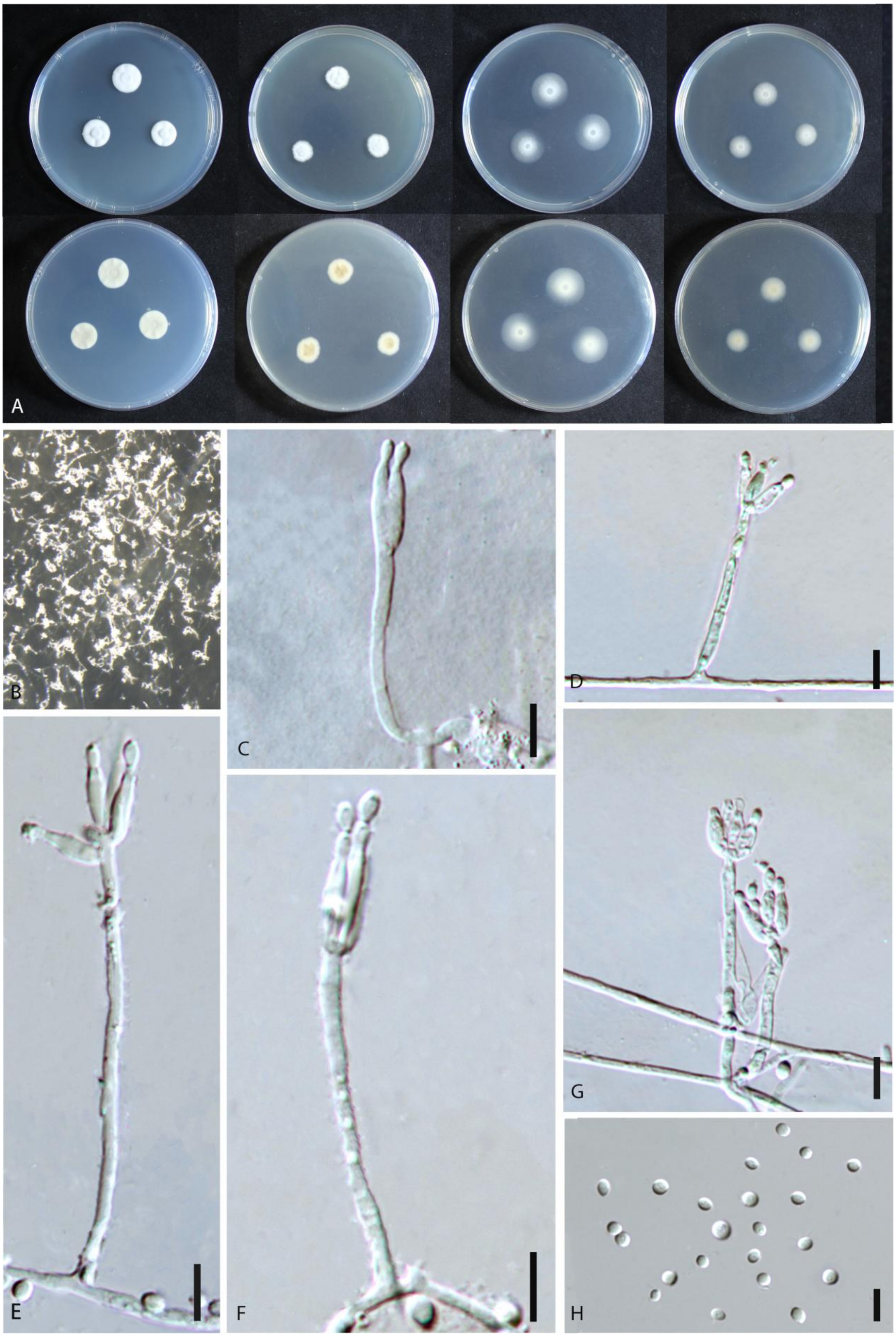

Fig. SD-11 ***Penicillium sedimenticola*** **(from ex-holotype CGMCC 3.22527)**. **A**Surface and reverse of colony on PDA, YES, MEA (25°C and MEA 35°C) . **B** Sporulation on MEA. **C–G** Conidiophores and conidiogenous cells. **H** Conidia. Scale bars: **C–H** = 5 µm.

Material examined: CHINA, Guangdong, National Mangrove Nature Reserve of Zhanjiang, isolated from sediment, November 2019, M. Li and J. E. Huang, HMAS 352453 (holotype designated here, dried culture), ex-type living culture CGMCC 3.22527 = LC15940; ibid., CGMCC 3.225278 = LC15941.

Notes: Phylogenetic analyses based on ITS, *tub2*, *cam* and *rpb2* sequences showed that our new species should be classified in *Penicillium* section *Cinnamopurpurea*. (Fig. SD-10). *P. sedimenticola* forms a distinct clade closely related to *P. incoloratum*, *P. shennongjianum* and *P. nodulum*, but can be distinguished from known species in producing fewer number of phialides in verticils (> 5 in other species) (Kong and Qi 1988; Huang and Qi 1994).

Talaromyces C.R. Benj., Mycologia 47(5): 681 (1955)

Talaromyces species are cosmopolitan, occurring in various environments, such as air, soil, living or rotten plants, and indoors. Some species produce enzymes and pigments of industrial importance, while some cause life-threatening mycosis. Currently, a total of 170 *Talaromyces* species are accepted in the genus, classified in 7 sections (Zhang et al. 2021). In this study, two new species are described as *T. ellipsoideus* and *T. phialiformis* in section *Trachyspermi* (Fig. SD-12).

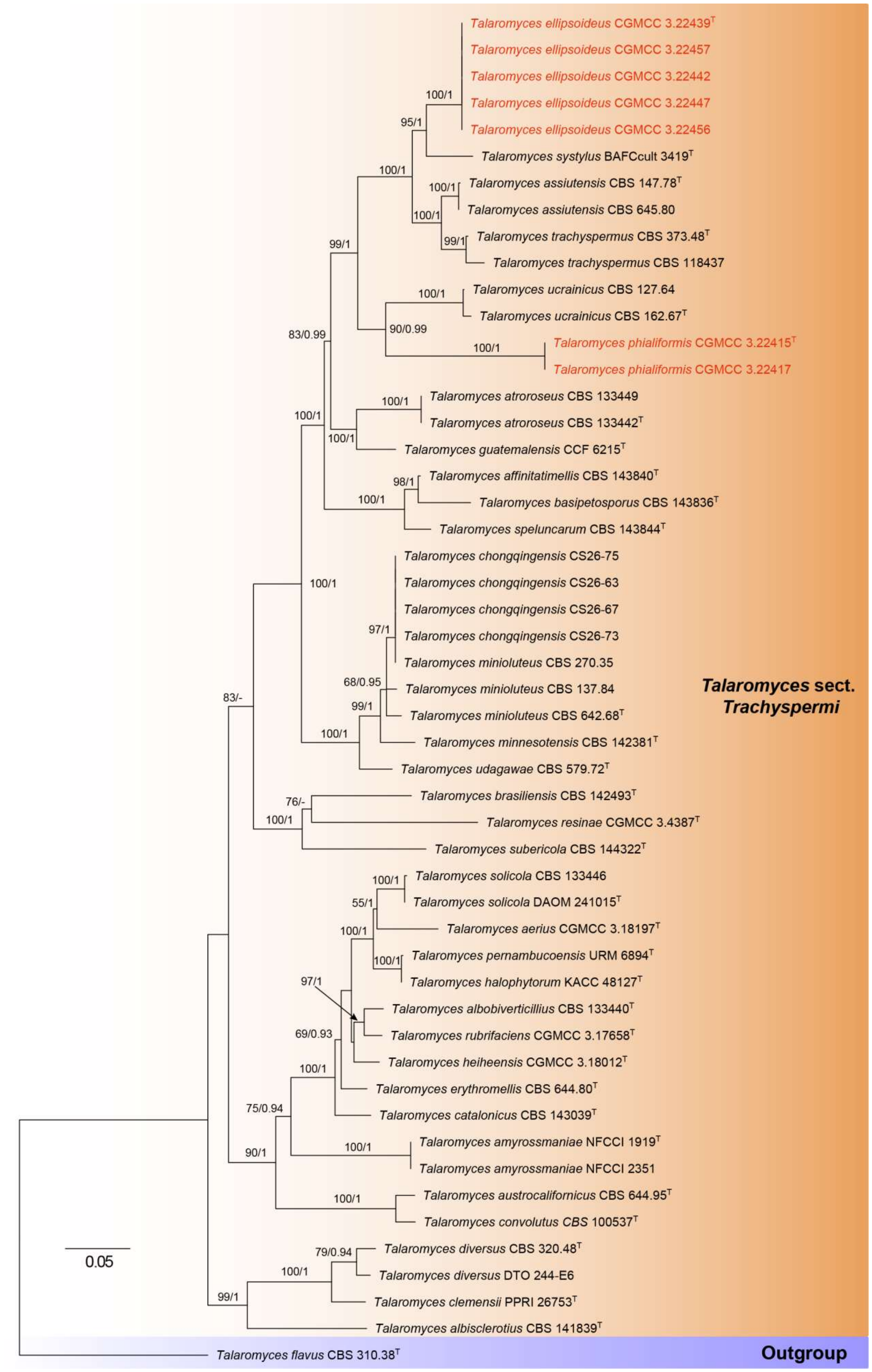

Fig. SD-12 Maximum likelihood tree of *Talaromyces* sect. *Trachyspermi* based on ITS, *cam*, *tub2* and *rpb2* sequences. The RAxML BS above 50% and BPP above 0.90 are presented at the nodes

BS/BPP. The tree was rooted to *Talaromyces flavus* CBS 310.38. Ex-type cultures are indicated with a letter "T" after the accession number. The new species are printed in red font.

Talaromyces ellipsoideus M. Li & L. Cai, ***sp. nov.***

Fungal Names: FN571503; Fig. SD-13

Etymology: Referring to the ellipsoidal conidia of this species.

Hyphae hyaline, septate, smooth, branched, 1.0–1.5 µm wide. *Synnemata* formed after 7 days on MEA up to 40–60 µm long. **Asexual morph** *Conidiophores* monoverticillate, with a low proportion biverticillate. *Stipes* smooth walled 19-31 × 1.5–2.0 µm, extra branches up to 25 µm long. *Metulae* two to six, divergent, 6.0–11 × 1.5–2.0 µm. *Phialides* acerose or narrowly ampulliform, two to six per metulae, 6.0–12 × 1.0–2.0 µm. *Conidia* formed in long chains, smooth, ellipsoidal, oval, 1.5–2.5 × 1.0–1.5 µm (av. = 2.1 ± 0.18 × 1.4 ± 0.13 µm, n = 50). **Sexual morph** not observed.

Culture characteristics—Colony diam: after 7 days at 25°C: on OA, 19–22 mm; on MEA, 22–24 mm; on DG18, 19–20 mm; on YES, 13–16 mm. Colony characteristics: On OA 25°C, 7 days: Colonies moderately deep, radially; margins low, moderately wide, entire; mycelia white; texture floccose; sporulation moderately dense; conidia *en masse* dull green; exudate absent; soluble pigment absent; reverse pale white. On MEA 25°C, 7 days: Colonies moderately deep, radially, flat; margins low, moderately wide, entire; mycelia white; texture velutinous; sporulation moderately dense; conidia *en masse* bluish to gray green; exudate absent; soluble pigment absent; reverse pale white. On DG18 at 25°C, after 7 days: Colonies moderately deep, radially, and protuberant incenters; margins low, moderately wide, entire; mycelia white; texture floccose; sporulation sparse to moderately dense; conidia *en masse* dull green; soluble pigments absent; exudates absent, reverse pale white. On YES at 25°C, after 7 days: Colonies moderately deep, radially, and protuberant incenters; margins low, moderately wide, entire; mycelia white; texture velutinous; sporulation absent; soluble pigments absent; exudates absent, reverse pale white.

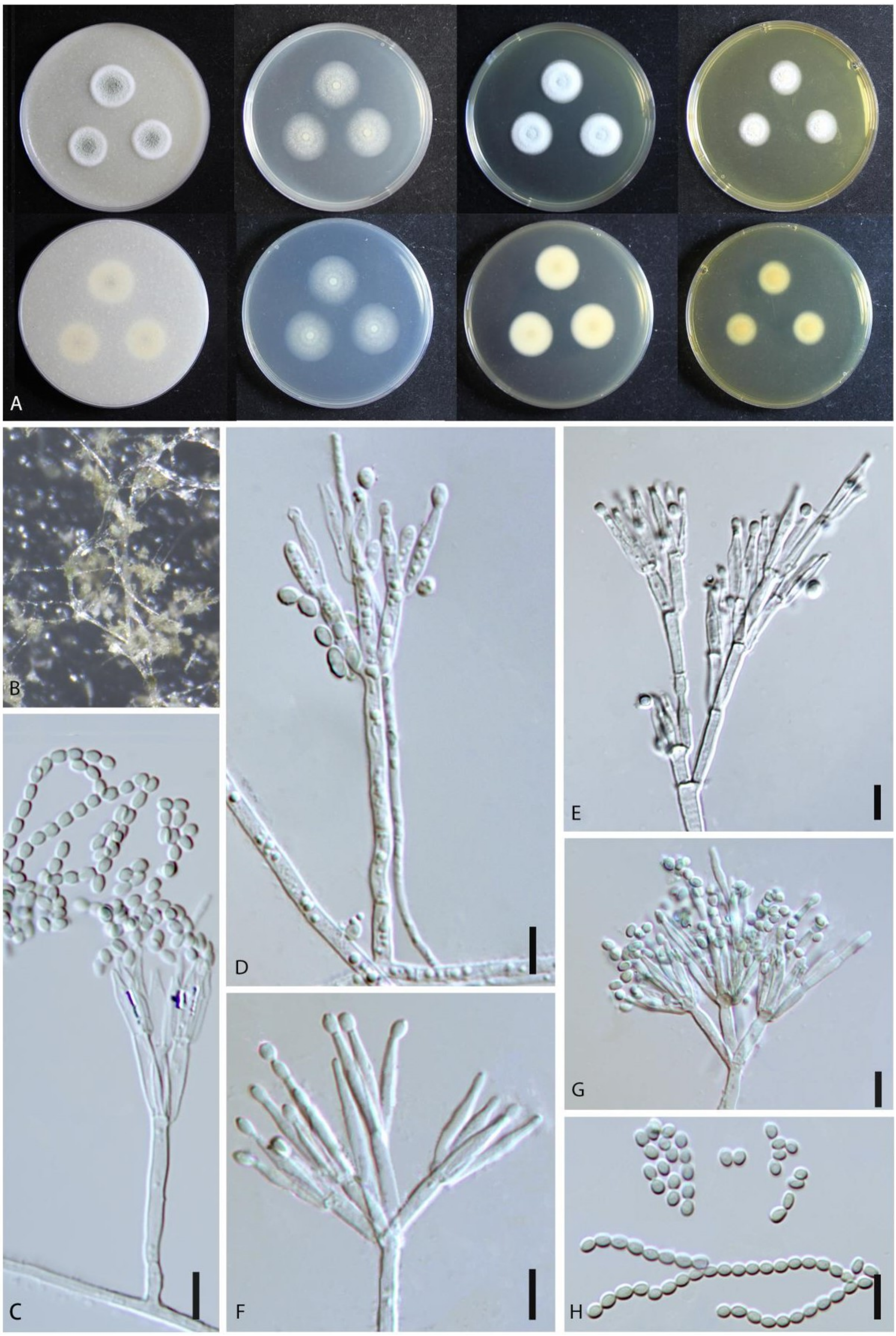

Fig. SD-13 ***Talaromyces ellipsoideus*** **(from ex-holotype CGMCC 3.22439)**. **A** Surface and reverse of colony on OA, MEA, DG18 and YES. **B** Sporulation on MEA. **C–G** Conidiophores and conidiogenous cells. **H** Conidia. Scale bars: **C–H** = 5 µm.

Material examined: CHINA, Guangdong, National Mangrove Nature Reserve of

Futian Shenzhen, isolated from sediment, September 2020, M. Li and Z. F. Zhang, HMAS 352444 (holotype designated here, dried culture), ex-type living culture CGMCC 3.22439 = LC15919; ibid., CGMCC 3.22457 = LC15923; ibid., CGMCC 3.22442 = LC15920; ibid., CGMCC 3.22447 = LC15921; ibid., CGMCC 3.22456 = LC15922.

Notes: Phylogenetic analyses based on ITS, *tub2*, *cam* and *rpb2* sequences placed our new species in *Talaromyces* section *Trachyspermi* (Fig. SD-12), closely related to *T. ellipsoideus* but differs in producing globose and rough-walled conidia (Romero et al. 2016).

Talaromyces phialiformis M. Li & L. Cai, ***sp. nov.***

Fungal Names: FN571504; Fig. SD-14

Etymology: Referring to its phialidic conidiogenous cells.

Hyphae hyaline, septate, smooth, branched, 1.5–2.0 µm wide. *Synnemata* formed after 7 days on MEA up to 20–70 µm long. **Asexual morph** *Conidiophores* monoverticillate, with a low proportion biverticillate. *Stipes* smooth walled 10–24 × 1.5–2.5 µm, extra branches up to 30 µm long. *Metulae* one to three, divergent, 7.0–18 × 1.5–2.5 µm. *Phialides* acerose or ampulliform, three to five per metulae, 7.0–14 × 1.5–2.0 µm. *Conidia* formed in chains, smooth, oval, spherical, 1.5–2.0 × 1.0–2.0 µm (av. = 1.8 ± 0.13 × 1.5 ± 0.12 µm, n = 50). **Sexual morph** not observed.

Culture characteristics—Colony diam: after 7 days at 25°C: on OA, 16–18 mm; on MEA, 15–17 mm; on DG18, 14–17 mm; on YES, 12–14 mm. Colony characteristics: On OA 25°C, 7 days: Colonies moderately deep, radially; margins moderately wide, entire; mycelia white; texture floccose; sporulation moderately dense; conidia *en masse* pale green; exudate absent; soluble pigment absent; reverse pale white. On MEA 25°C, 7 days: Colonies moderately deep, radially, and protuberant incenters; margins low, moderately wide, irregular; mycelia white; texture velutinous; sporulation sparse to moderately dense; conidia *en masse* pale green; exudate absent; soluble pigment absent; reverse pale white. On DG18 at 25°C, after 7 days: Colonies moderately deep, radially; margins low, narrow, entire; mycelia white;

texture floccose; sporulation dense; conidia *en masse* dull green; soluble pigments absent; exudates absent, reverse pale white. On YES at 25°C, after 7 days: Colonies moderately deep, radially, and protuberant incenters; margins low, wide, entire; mycelia white; texture velutinous; sporulation absent; soluble pigments absent; exudates absent, reverse pale white.

Material examined: CHINA, Guangdong, National Mangrove Nature Reserve of Futian Shenzhen, isolated from sediment, September 2020, M. Li and Z. F. Zhang, HMAS 352442 (holotype designated here, dried culture), ex-type living culture CGMCC 3.22415 = LC15912; ibid., CGMCC 3.22417 = LC15913.

Notes: Phylogenetic analyses based on ITS, *tub2*, *cam* and *rpb2* sequences placed our new species in *Talaromyces* section *Trachyspermi*. (Fig. SD-12), closely related to *T. ucrainicus.* Morphologically, metulae of *T. phialiformis* is narrower than that of *T. ucrainicus* (1.5–2.5 µm *vs*. 3–3.5 µm). Meanwhile, conidia of *T. phialiformis* are much smaller than that of *T. ucrainicus* (1.5–2.0 × 1.0–2.0 µm *vs*. 2.5–4.8 × 2.5–3.5 µm) (Panasenko 1964).

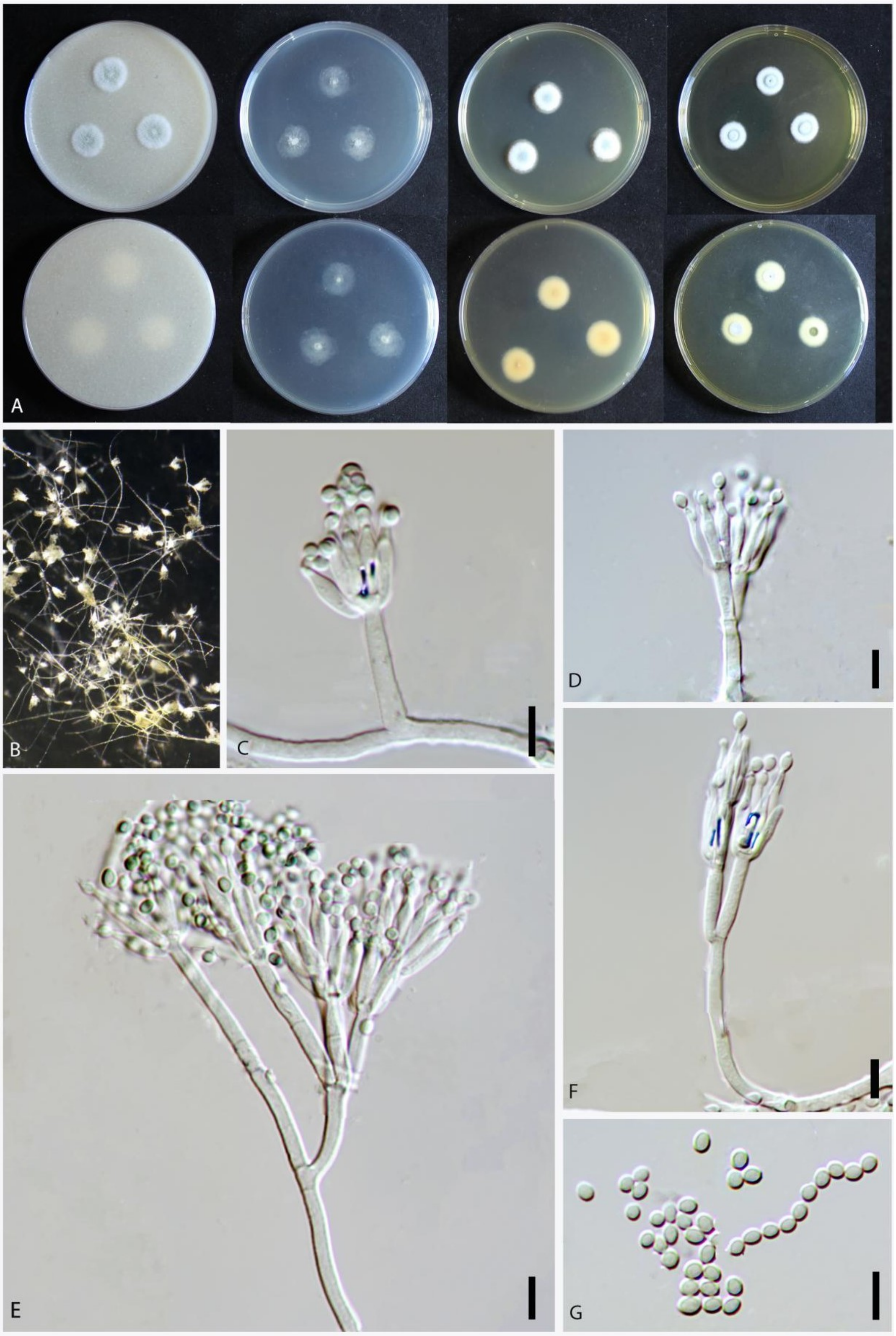

Fig. SD-14 *Talaromyces phialiformis* (from ex-holotype CGMCC 3.22415). **A** Surface and reverse of colony on OA, MEA, DG18 and YES. **B** Sporulation on MEA. **C–F** Conidiophores and conidiogenous cells. **G** Conidia. Scale bars: **C–G** = 5 μm.

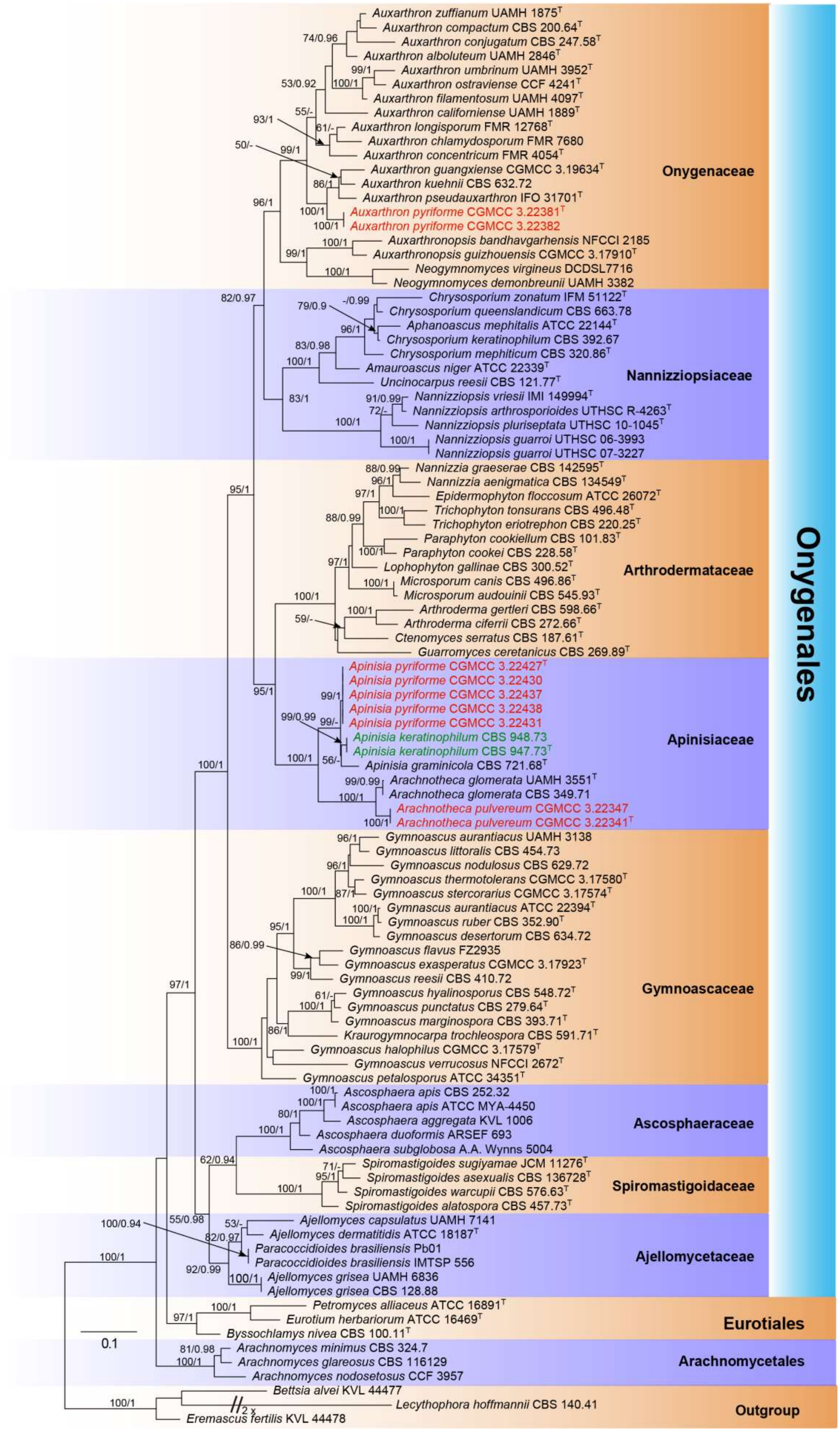

Fig. SD-15 Maximum likelihood tree of Onygenales based on ITS and LSU sequences. The RAxML BS above 50% and BPP above 0.90 are presented at the nodes BS/BPP. The tree was

rooted to *Eremascus fertilis* KVL 44478, *Bettsia alvei* KVL 44477 and *Lecythophora hoffmannii* CBS 140.41. Ex-type cultures are indicated with a letter "T" after the accession number. The new species are printed in red font and new combination in green font.

Onygenales Cif. ex Benny & Kimbr., Mycotaxon 12(1): 8 (1980)

Apinisiaceae M. Li, M. Raza & L. Cai, ***fam. nov.***

Fungal Names: FN571505

Asexual morph *Conidiophores* absent or erect, hyaline, solitary, cylindrical, straight to slightly flexuous, thickened at septa, smooth-walled. *Conidiogenous cells* monoblastic or polyblastic, intercalary or terminal, simple or branched, hyaline, smooth- and thin-walled, determinate, discrete, cylindrical. *Conidia* globose, oval to pyriform, sometimes arthroconidia, oblong to sub-cylindrical, straight or slightly curved, smooth- and thin-walled, hyaline, aseptate. **Sexual morph** *Ascogonium* composed of a straight, thickened, often clavate cell, surrounded by a densely coiled antheridium. *Ascomata* spherical, lanose, light, with a peridium composed of rather thick, anastomosing hyphal elements. *Asci* spherical or nearly so, borne on croziers, thin-walled, 8-spored. *Ascospores* spherical, hyaline to yellow, small, with a rather thick wall surrounded by an irregularly furrowed sheath.

Type: Apinisia La Touche

Notes: In this study, phylogenetic analyses based on ITS and LSU sequences showed that five species isolated from sediment, soil and plants are clearly separate from all known families in *Onygenales* (Fig. SD-15), and therefore *Apinisiaceae* is introduced to accommodate *Apinisia* and *Arachnotheca*. Phylogenetically, the new family is represented by a distinct clade closely related to *Arthrodermataceae*. However, species in the *Arthrodermataceae* are mostly isolated from animals or humans which cause fungal infections. In addition, species in the *Arthrodermataceae* often have both micro- and arthroconidia or micro- and macroconidia while *Apinisiaceae* is characterized by microconidia or arthroconidia (Howard et al. 2002).

Apinisia La Touche 1968

Asexual morph *Conidiophores* absent. *Conidiogenous cells* phialidic, monoblastic or polyblastic, intercalary or terminal, hyaline, smooth- and thin-walled, determinate, discrete, cylindrical. *Conidia* globose, oval to pyriform, smooth- and thin-walled, hyaline, aseptate. **Sexual morph** *Cleistothecia* white, globose, when aggregate, globose to ovate, excluding appendages. *Peridium* composed of hyaline irregular hyphae with thin walls, but forming unequal chlamydospores in chains which become free at maturity. *Ascospores* yellow, globose with lightly or finely echinulate walls.

Type: Apinisia graminicola La Touche

Notes: *Apinisia* is herein introduced to accommodate *A. keratinophilum, A. graminicola* and *A. pyriforme*. Three species clustered together in an independent clade sister to *Arachnotheca* (Fig. SD-15). Morphologically, two genera are different in the type of conidiogenous cells and conidia (monoblastic or polyblastic conidiogenous cells and globose, oval to pyriform conidia in *Apinisia*; simple or branched conidiogenous cells and arthroconidia conidia in *Arachnotheca*).

Apinisia keratinophilum (Samson & Polon) M. Li & L. Cai, ***comb. nov.***

Fungal Names: FN571506

Basionym: *Myriodontium keratinophilum* Samson & Polon., Persoonia 9 (4): 505 (1978).

Holotype: Roma, Orto Botanico, on soil, IMI 160282, ex-type living culture, CBS 947.73.

Notes: Myriodontium keratinophilum was described in family and order incertae sedis based on morphological characteristics (Samson and Polonelli 1978). Phylogenetic analysis in this study revealed that *M. keratinophilum* clustered in a distinct and well-supported clade in *Apinisia* (Fig. SD-15) and is thus treated as a new combination.

Apinisia pyriforme M. Li, M. Raza & L. Cai, ***sp. nov.***

Fungal Names: FN571507; Fig. SD-16

Etymology: Referring to the pyriform conidia of the fungus.

Hyphae thin walled, hyaline, septate, smooth, branched, anastomosis, 1,0–3.0 μm wide. **Asexual morph** *Conidiophores* absent. *Conidiogenous cells* phialidic, monoblastic, terminal, hyaline, smooth- and thin-walled, determinate, discrete, cylindrical, 13–34 × 1.5–3.0 μm. *Conidia* oval to pyriform, globose, smooth- and thin-walled, hyaline, aseptate, 2.0–2.5 × 2.0–2.5 μm (av. = 2.3 ± 0.09 × 2.1 ± 0.12 μm, n = 30). **Sexual morph** not observed.

Culture characteristics—Colonies on PDA attaining 67–69 mm diam. after 3 weeks, felty to pulverulent, annular, margin undulate, white, aerial mycelia sparse. Reverse white to yellowish brown. Sporulation within 3 weeks on PDA. Colonies on MEA attaining 54–56 mm diam. after 3 weeks, flat, annular, margin rhizoids, white, aerial mycelia sparse. Reverse white. Colonies on OA attaining 35–36 mm diam. after 3 weeks, flat, felty, annular, margin slightly undulate, white, aerial mycelia sparse. Reverse yellowish brown.

Material examined: CHINA, Guangdong, National Mangrove Nature Reserve of Futian Shenzhen, isolated from sediment, September 2020, M. Li and Z. F. Zhang, HMAS 352443 (holotype designated here, dried culture), ex-type living culture CGMCC 3.22427 = LC15914; ibid., CGMCC 3.22430 = LC15915; ibid., CGMCC 3.22431 = LC15916; ibid., CGMCC 3.22437 = LC15917; ibid., CGMCC 3.22438 = LC15918.

Notes: Phylogenetically, *Apinisia pyriforme* is most closely related to *A. pyriforme* and *A. graminicola* (Fig. SD-15). However, *A. pyriforme* is distinguishable from *A. pyriforme* by the monoblastic and terminal conidiogenous cells, whereas *A. pyriforme* produces polyblastic and terminal or intercalary conidiogenous cells. Meanwhile, *A. pyriforme* produces conidiogenous cells with cylindrical conduits radiating on all sides, which were not observed in *A. pyriforme*. Furthermore, *A. pyriforme* can be distinguished from *A. pyriforme* by the shape and the size of its conidia, being globose, oval to pyriform, measuring 2.0–2.5 × 2.0–2.5 μm in *A. pyriforme*, whereas *A. pyriforme* produces subglobose conidia, measuring 2-3 μm in diam (Samson and Polonelli 1978). Sexual morph of *A. pyriforme* has not been

observed, unlike *A. graminicola.*

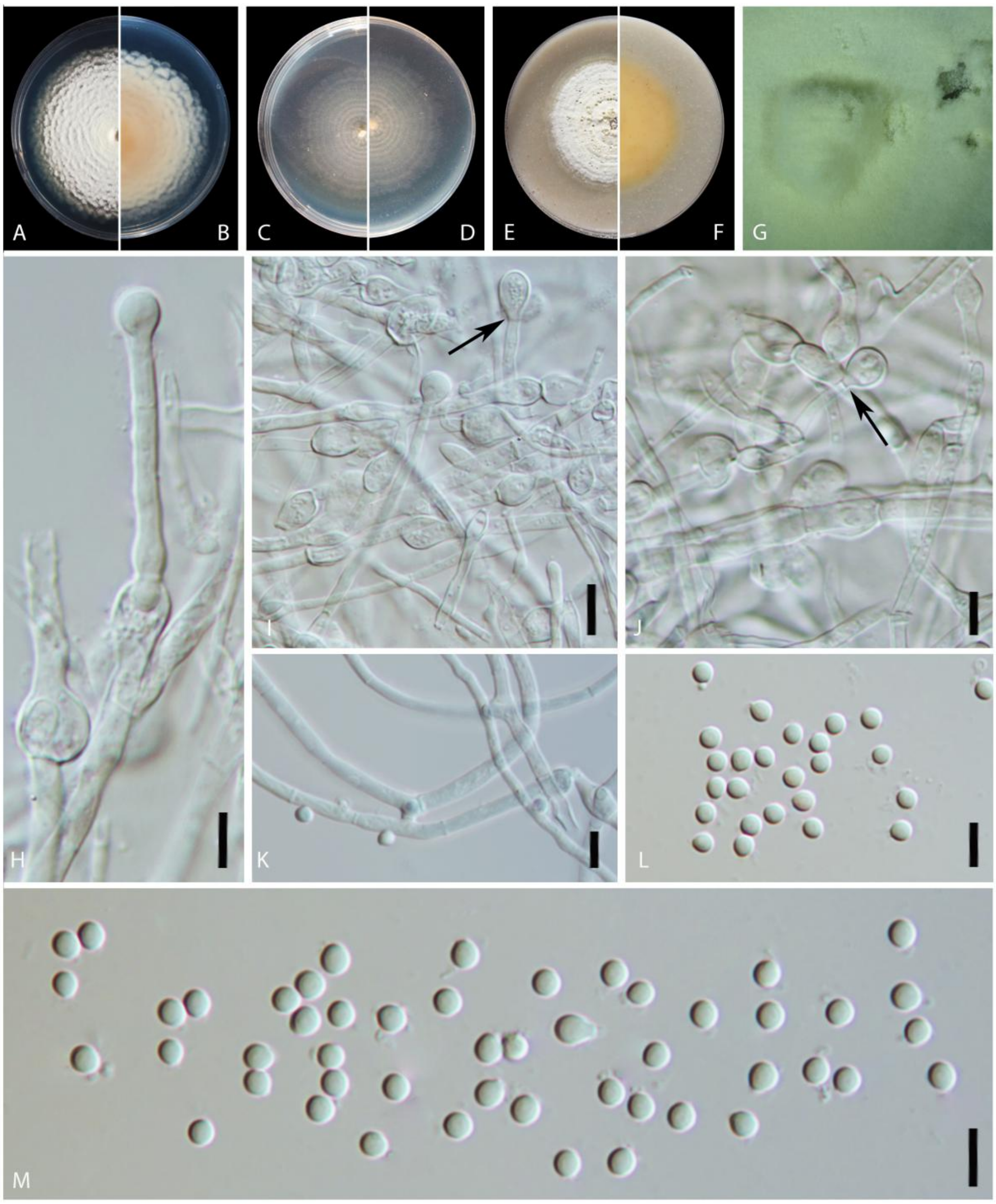

Fig. SD-16 *Apinisia pyriforme* (from ex-holotype CGMCC 3.22427). **A–F** Surface and reverse of colony on PDA, MEA and OA. **G** Sporulation on PDA. **H–J** Conidiogenous cells and racquet hyphae. **K** Anastomosis hyphae. **L–M** Conidia. Scale bars: **H–M** = 5 µm.

Arachnotheca Arx, Persoonia 6(3): 376 (1971)

Asexual morph *Conidiophores* simple, hyaline, solitary, cylindrical, straight to slightly flexuous, thickened at septa, smooth-walled. *Conidiogenous cells* simple or branched, smooth- and thin-walled, forming septa basipetally to form arthroconidia released via schizolythic secession. *Conidia* arthroconidia, unicellular, thin- and

smooth-walled, hyaline, oblong to sub-cylindrical, straight or slightly curved. **Sexual morph** *Ascogonium* composed of a straight, thickened, often clavate cell, surrounded by a densely coiled antheridium. *Ascomata* spherical, lanose, light, with a peridium composed of rather thick, anastomosing hyphal elements. *Asci* spherical or nearly so, borne on croziers, thin-walled, 8-spored. *Ascospores* spherical, hyaline, small, with a rather thick wall surrounded by an irregularly furrowed sheath.

Type: Arachnotheca glomerata (E. Müll. & Pacha-Aue) Arx

Notes: *Arachnotheca*, represented by *A. pulvereum* and *A. glomerata,* clustered as a sister clade to *Apinisia* (Fig. SD-15).

Arachnotheca pulvereum M. Li, M. Raza & L. Cai, ***sp. nov.***

Fungal Names: FN571508; Fig. SD-17

Etymology: Referring to the powdery conoly on PDA medium.

Hyphae hyaline, thin-walled, smooth, septate, branched, anastomosis, 2.0–4.0 µm wide. **Asexual morph** *Conidiomata* superficial, subglobose or other irregular shaped, pale yellow. *Conidiophores* erect, simple, hyaline, solitary, cylindrical, straight to slightly flexuous, thickened at septa, up to 18 µm long, smooth-walled. *Conidiogenous hyphae* simple or branched, 1.5–2.0 µm wide, smooth- and thin-walled, forming septa basipetally to form arthroconidia released via schizolythic secession. *Conidia* arthroconidia unicellular, thin- and smooth-walled, hyaline, oval, ellipsoidal to sub-cylindrical, straight or slightly curved, 2.0–3.5 × 1.5–2.5 µm (av. = 2.5 ± 0.43 × 1.8 ± 0.23 µm, n = 30). **Sexual morph** not observed.

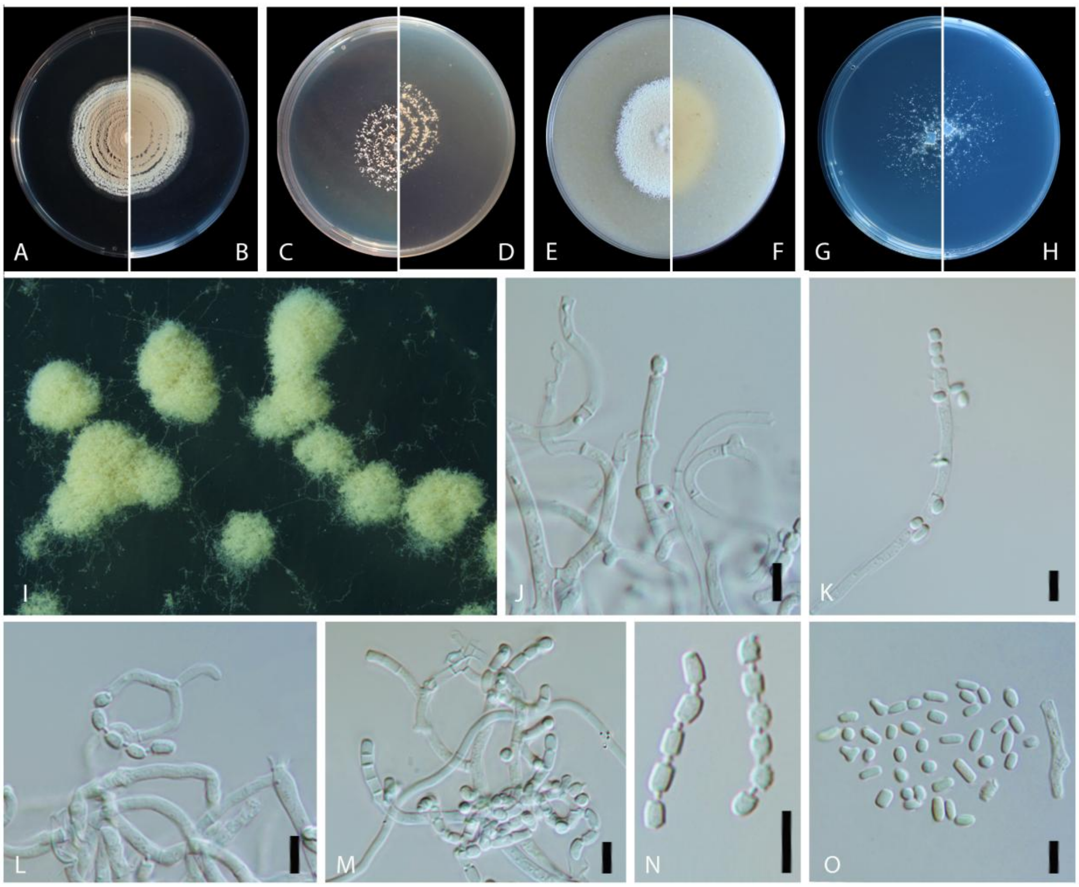

Fig. SD-17 *Arachnotheca pulvereum* (from ex-holotype CGMCC 3.22341). **A–H** Surface and reverse of colony on PDA, MEA, OAand SNA. **I** Conidiomata on MEA. **J–M** Conidiophores and conidiogenous cells. **N–O** Arthroconidia. Scale bars: **J–O** = 5 μm.

Culture characteristics—Colonies on PDA attaining 46–48 mm diam. after 3 weeks, flat, powdery, annular, margin entire, white to pale brown, aerial mycelia sparse. Reverse white to pale brown. Colonies on MEA attaining 38–40 mm diam. after 3 weeks, flat, powdery, annular, margin rhizoids, with white to pale yellow conidiomata scattered, aerial mycelia sparse. Reverse white to pale yellow. Sporulation within 3 weeks on MEA. Colonies on OA attaining 38–43 mm diam. after 3 weeks, flat, powdery, margin entire, white to pale brown, aerial mycelia sparse. Reverse white to pale brown. Colonies on SNA attaining 38–40 mm diam. after 3 weeks, powdery, margin rhizoids, with white to pale yellow conidiomata scattered, aerial mycelia sparse. Reverse white to pale yellow. Sporulation within 3 weeks on SNA.

Material examined: CHINA, Guangdong, National Mangrove Nature Reserve of

Futian Shenzhen, isolated from sediment, September 2020, M. Li and Z. F. Zhang, HMAS 352433 (holotype designated here, dried culture), ex-type living culture CGMCC 3.22341 = LC15889; ibid., CGMCC 3.22347 = LC15890.

Notes: Arachnotheca pulvereum is phylogenetically most closely related to *A. glomerata* (Fig. SD-15), but differ in the low sequence similarity (94.2% similarity, 32 base pairs (bp) difference in 544 bp of ITS; 98.6% similarity, 12 bp difference in 845 bp of LSU). Morphologically *A. pulvereum* is characterized by oval, ellipsoidal to sub-cylindrical conidia, whereas *A. glomerata* produces consistently cylindrical conidia.

Onygenaceae Berk., Intr. crypt. bot. (London): 272 (1857)

The family *Onygenaceae* is characterised by pseudoparenchymatous and membranous cleistothecia or a filamentous gymnothecia of loosely interwoven hyphae with a structure similar to *Gymnoascaceae*. Morphologically, the sexual morph of *Onygenaceae* is characterized by oblate, spherical, reniform, punctate or pitted ascospores, and the asexual morph is characterized by hyphomycetes with arthro- and aleurioconidia (Doveri et al. 2012).

Auxarthron G.F. Orr & Kuehn, Can. J. Bot. 41: 1439 (1963)

The genus *Auxarthron* was established by Orr et al. (1963) and placed in *Gymnoascaceae*. Subsequent molecular phylogenetic studies showed that it actually belongs to *Onygenaceae* (Sugiyama et al. 1999; Sigler et al. 2002). Currently, a total of 21 species are accepted in *Auxarthron*. In this study, one new species is described as *Auxarthron pyriforme* (Fig. SD-15).

Auxarthron pyriforme M. Li, M. Raza & L. Cai, ***sp. nov.***

Fungal Names: FN571509; Fig. SD-18

Etymology: Referring to the pyriform conidia of the fungus.

Hyphae smooth, hyaline, branched, septate, 2.0–2.5 µm wide. **Asexual morph** *Arthroconidia* abundant, unicellular, intercalary, terminal or lateral, straight or curved, solitary, intercalary, terminal or lateral, pyriform to globose, sometime irregularly swollen smooth and fairly thick-walled, 2.0–6.5 × 2.0–4.0 µm (av. = 3.1 ± 0.85 × 2.6

± 0.46 µm, n = 30), frequently separated by 1–5 autolytic connective cells. **Sexual morph** not observed.

Culture characteristics—Colonies on PDA attaining 24–26 mm diam. after 3 weeks, felty to pulverulent, plicated and raised at center, margin slightly undulate, white to pinkish white, aerial mycelia sparse. Reverse white to dark brown. Sporulation within 3 weeks on PDA. Colonies on MEA attaining 5–7 mm diam. after 3 weeks, flat, margin irregular, white to yellowish brown, aerial mycelia sparse. Reverse white to yellowish brown. Colonies on OA attaining 60–62 mm diam. after 3 weeks, flat, powdery, margin irregular, white to brown, aerial mycelia sparse. Reverse brown. Colonies on SNA attaining 12–15 mm diam. after 3 weeks, powdery, margin rhizoids, white, aerial mycelia extremely sparse. Reverse white.

Material examined: CHINA, Guangdong, National Mangrove Nature Reserve of Futian Shenzhen, isolated from sediment, September 2020, M. Li and Z. F. Zhang, HMAS 352436 (holotype designated here, dried culture), ex-type living culture CGMCC 3.22381 = LC15897; ibid., CGMCC 3.22382 = LC15898.

Notes: *Auxarthron pyriforme* is closely related to *A. kuehnii*, *A. guangxiense* and *A. pseudauxarthron*. *Auxarthron kuehnii*, *A. guangxiense* and *A. pseudauxarthron* only produce sexual morph. In contrast, *A. pyriforme* only produces asexual morph. *A. pyriforme* is characterized by pyriform to globose arthroconidia which were not observed from other known species in *Auxarthron* (Hubka et al. 2013; Crous et al. 2013; Sarrocco et al. 2015; Zhang et al. 2020).

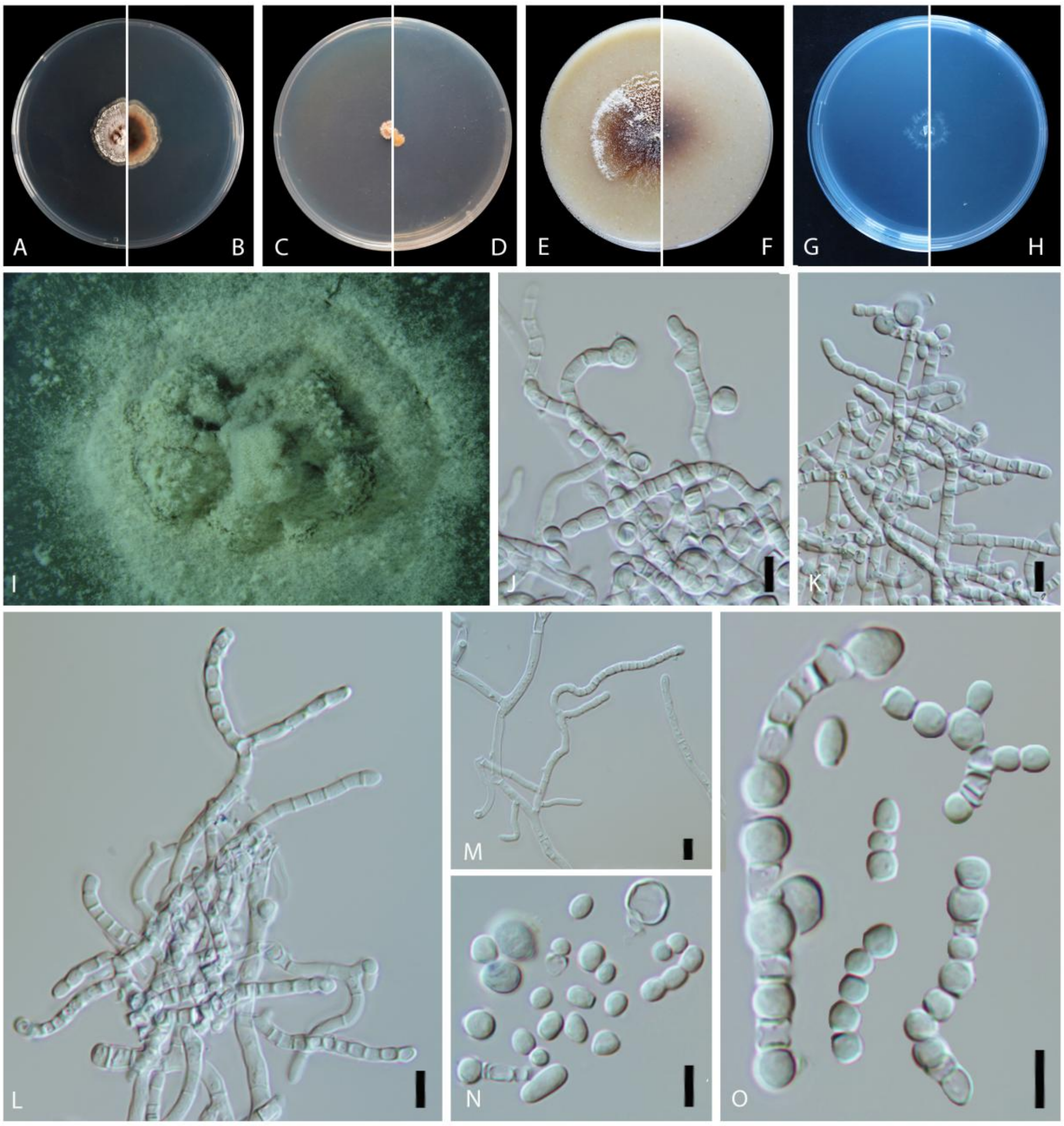

Fig. SD-18 ***Auxarthron pyriforme*** **(from ex-holotype CGMCC 3.22381)**. **A–H** Surface and reverse of colony on PDA, MEA, OA and SNA. **I** Sporulation on PDA. **J–O** Arthroconidia. Scale bars: **J–O** = 5 μm.

Class Sordariomycetes O.E. Erikss & Winka, Myconet 1(1): 10 (1997)

Subclass Hypocreomycetidae O.E. Erikss & Winka, Myconet 1(1): 6 (1997)

Hypocreales Lindau, Nat. Pflanzenfam., Teil. I (Leipzig) 1(1): 343 (1897)

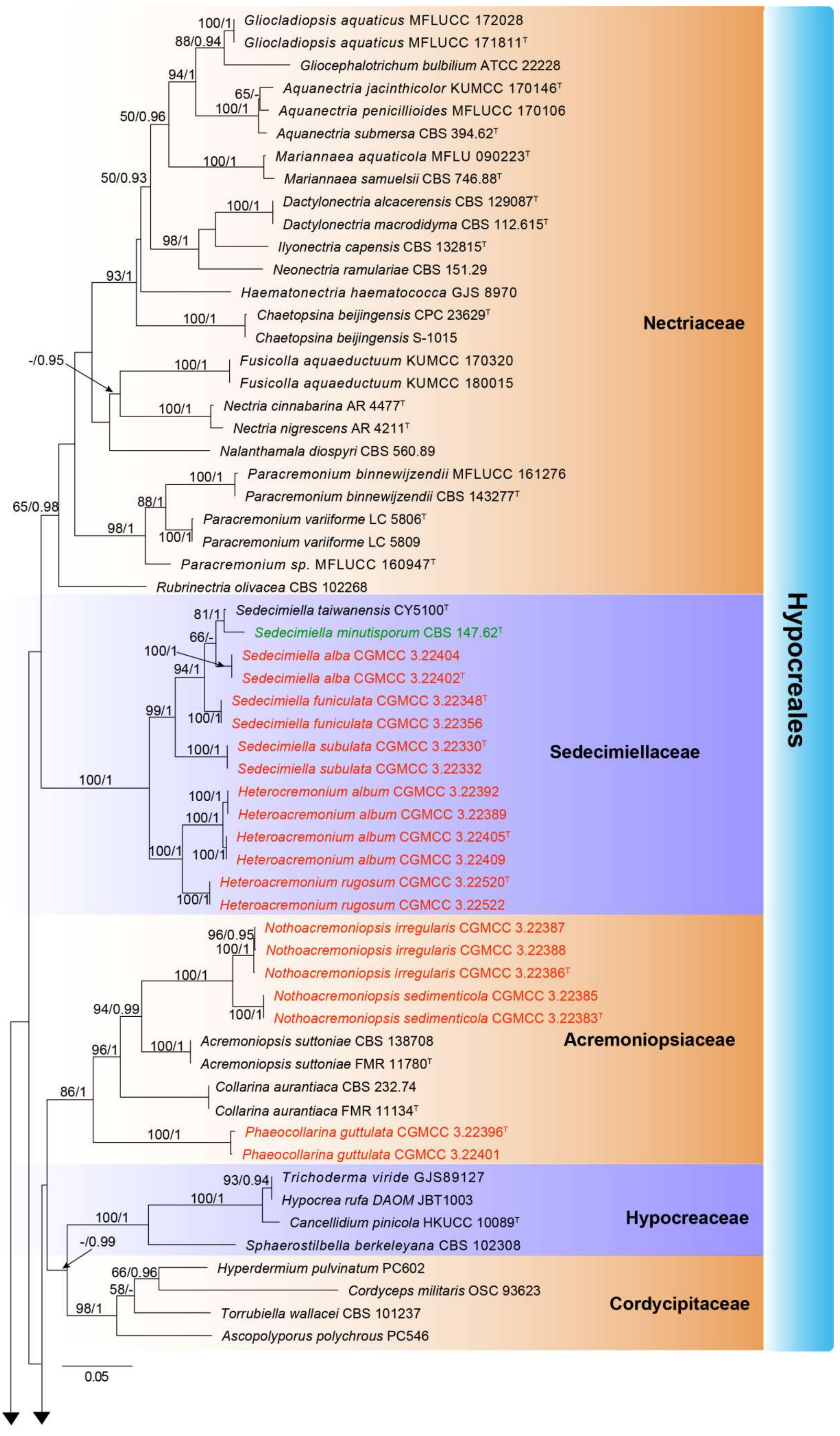
Gliocladiopsis aquaticus MFLUCC 172028
Gliocladiopsis aquaticus MFLUCC 171811T
Gliocephalotrichum bulbilium ATCC 22228
Aquanectria jacinthicolor KUMCC 170146T
Aquanectria penicillioides MFLUCC 170106
Aquanectria submersa CBS 394.62T
Mariannaea aquaticola MFLU 090223T
Mariannaea samuelsii CBS 746.88T
Dactylonectria alcacerensis CBS 129087T
Dactylonectria macrodidyma CBS 112.615T
Ilyonectria capensis CBS 132815T
Neonectria ramulariae CBS 151.29
Haematonectria haematococca GJS 8970
Chaetopsina beijingensis CPC 23629T
Chaetopsina beijingensis S-1015
Fusicolla aquaeductuum KUMCC 170320
Fusicolla aquaeductuum KUMCC 180015
Nectria cinnabarina AR 4477T
Nectria nigrescens AR 4211T
Nalanthamala diospyri CBS 560.89
Paracremonium binnewijzendii MFLUCC 161276
Paracremonium binnewijzendii CBS 143277T
Paracremonium variiforme LC 5806T
Paracremonium variiforme LC 5809
Paracremonium sp. MFLUCC 160947T
Rubrinectria olivacea CBS 102268
Nectriaceae
Sedecimiella taiwanensis CY5100T
Sedecimiella minutisporum CBS 147.62T
Sedecimiella alba CGMCC 3.22404
Sedecimiella alba CGMCC 3.22402T
Sedecimiella funiculata CGMCC 3.22348T
Sedecimiella funiculata CGMCC 3.22356
Sedecimiella subulata CGMCC 3.22330T
Sedecimiella subulata CGMCC 3.22332
Heterocremonium album CGMCC 3.22392
Heteroacremonium album CGMCC 3.22389
Heteroacremonium album CGMCC 3.22405T
Heteroacremonium album CGMCC 3.22409
Heteroacremonium rugosum CGMCC 3.22520T
Heteroacremonium rugosum CGMCC 3.22522
Sedecimiellaceae
Nothoacremoniopsis irregularis CGMCC 3.22387
Nothoacremoniopsis irregularis CGMCC 3.22388
Nothoacremoniopsis irregularis CGMCC 3.22386T
Nothoacremoniopsis sedimenticola CGMCC 3.22385
Nothoacremoniopsis sedimenticola CGMCC 3.22383T
Acremoniopsis suttoniae CBS 138708
Acremoniopsis suttoniae FMR 11780T
Collarina aurantiaca CBS 232.74
Collarina aurantiaca FMR 11134T
Phaeocollarina guttulata CGMCC 3.22396T
Phaeocollarina guttulata CGMCC 3.22401
Acremoniopsiaceae
Trichoderma viride GJS89127
Hypocrea rufa DAOM JBT1003
Cancellidium pinicola HKUCC 10089T
Sphaerostilbella berkeleyana CBS 102308
Hypocreaceae
Hyperdermium pulvinatum PC602
Cordyceps militaris OSC 93623
Torrubiella wallacei CBS 101237
Ascopolyporus polychrous PC546
Cordycipitaceae
Hypocreales
0.05

Fig. SD-19 Maximum likelihood tree of Hypocreales based on ITS, LSU, SSU, *rpb2* and *tef1* sequences. The RAxML BS above 50% and BPP above 0.90 are presented at the nodes BS/BPP. The tree was rooted to *Marinokulati chaetosa* BCRCFU30271. Ex-type cultures are indicated with a letter "T" after the accession number. The new species are printed in red font and new combination in green font.

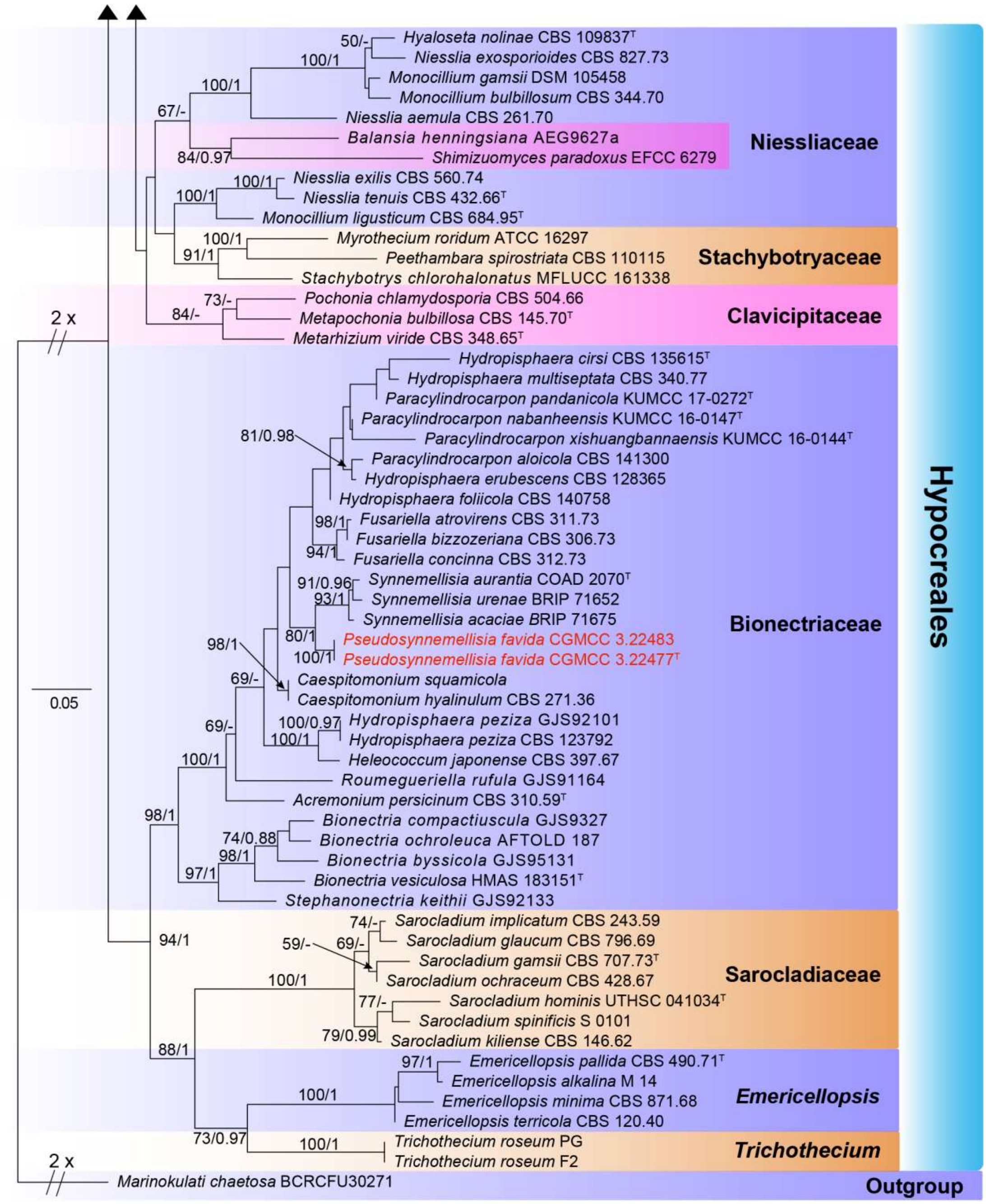

Fig. SD-19. (Continued).

Acremoniopsiaceae M. Li, M. Raza & L. Cai, **fam. nov.**

Fungal Names: FN571510

Asexual morph *Conidiophores* simple or branched, emerging laterally or terminally from vegetative hyphae or ropes of hyphae, straight or flexuous. *Conidiogenous cells* phialidic, solitary, cylindrical to subulate, smooth- and thin-walled, hyaline, monophialidic or sympodially proliferating polyphialides, tapering towards at the apex. *Conidia* unicellular, smooth- and thin-walled, hyaline, globose or subglobose, arranged in heads. **Sexual morph** not observed.

Type: Acremoniopsis A. Giraldo, Gené & Guarro

Notes: *Acremoniopsiaceae* is herein proposed to accommodate four genera: *Acremoniopsis* that was previously placed in family incertae sedis, *Collarina* that was previously placed in *Clavicipitaceae,* and two new genera *Nothoacremoniopsis* and *Phaeocollarina* described in this study. Phylogenetic analyses based on multi-locus sequences showed *Acremoniopsiaceae* clustered in *Hypocreales* as a distinct clade separated from other known families in *Hypocreales* (Fig. SD-19).

Collarina A. Giraldo, Gené & Guarro, in Crous et al., Persoonia 33: 271 (2014)

Notes: Based on morphology and 18S rDNA sequence analyses, Crous et al. (2014) introduced a monotypic genus *Collarina* in the family *Clavicipitaceae*. Subsequently, *Collarina* is considered a synonym of *Niesslia* (Niessliaceae) based on morphology and ITS sequence analyses (Gams et al. 2019). In our multi-locus phylogenetic tree, the genus formed a well-supported distinct lineage in the newly established family *Acremoniopsiaceae* (Fig. SD-19).

Nothoacremoniopsis M. Li, M. Raza & L. Cai, ***gen. nov.***

Fungal Names: FN571511

Etymology: Referring to the close phylogenetic relationship with the genus *Acremoniopsis*.

Asexual morph *Conidiophores* simple or branched, emerging laterally or terminally from vegetative hyphae or ropes of hyphae, straight or flexuous. *Conidiogenous cells* phialidic, solitary, cylindrical to subulate, smooth- and

thin-walled, hyaline, sympodially proliferating polyphialides, tapering towards at the apex. *Conidia* unicellular, smooth- and thin-walled, hyaline, globose or subglobose, arranged in heads. **Sexual morph** not observed.

Type: Nothoacremoniopsis sedimenticola M. Li, M. Raza & L. Cai

Notes: *Nothoacremoniopsis* is herein introduced to accommodate *N. sedimenticola* and *N. irregularis*. Two species clustered together in an independent clade sister to *Acremoniopsis* (Fig. SD-19). Morphologically, two genera are different in the rugose and monophialidic *vs*. smooth and polyphialidic conidiogenous cells (Crous et al. 2014).

Nothoacremoniopsis irregularis M. Li, M. Raza & L. Cai, ***sp. nov.***

Fungal Names: FN571512; Fig. SD-20

Etymology: Referring to the irregular conidia of the fungus.

Hyphae smooth, branched, septate, thin-walled, hyaline, 1.0–1.5 µm wide. **Asexual morph** *Conidiomata* superficial, bowl-shaped, pinkish, normally surrounded by hyphal hairs. *Conidiophores* simple or branched, emerging laterally or terminally from vegetative hyphae or ropes of hyphae, straight or flexuous. *Conidiogenous cells* phialidic, solitary, cylindrical to subulate, smooth- and thin-walled, hyaline, sympodially proliferating polyphialides, 10–29 µm long, 1.0–1.5 µm wide at the base, tapering towards at the apex. *Conidia* unicellular, smooth- and thin-walled, hyaline, oblong to cylindrical, ellipsoidal to subglobose, or irregular shaped, 1.5–2.5 × 1.0–2.0 µm (av. = 1.8 ± 0.26 × 1.5 ± 0.2 µm, n = 30), arranged in heads. *Chlamydospores* not observed. **Sexual morph** not observed.

Culture characteristics—Colonies on PDA attaining 29–31 mm diam. after 3 weeks, flat, felty, annular, margin entire, white to light pink, aerial mycelia sparse. Reverse white to light pink. Sporulation within 3 weeks on PDA. Colonies on MEA attaining 30–32 mm diam. after 3 weeks, flat, felty to pulverulent, margin entire, white, aerial mycelia extremely sparse. Reverse white. Colonies on OA attaining 24–27 mm diam. after 3 weeks, flat, felty to pulverulent, margin slightly undulate, white to light pink, aerial mycelia extremely sparse. Reverse white to light pink.

Material examined: CHINA, Guangdong, National Mangrove Nature Reserve of Futian Shenzhen, isolated from sediment, September 2020, M. Li and Z. F. Zhang, HMAS 352438 (holotype designated here, dried culture), ex-type living culture CGMCC 3.22386 = LC15901; ibid., CGMCC 3.22387 = LC15902; ibid., CGMCC 3.22388 = LC15903.

Notes: Phylogenetically, *Nothoacremoniopsis irregularis* is most closely related to *N. sedimenticola*, another novel species described in this study (Fig. SD-19). While, *N. irregularis* is distinguishable from *N. sedimenticola* by the shape and size of its conidia, being various shaped (oblong to cylindrical, ellipsoidal to subglobose) measuring 1.5–2.5 × 1.0–2.0 µm, whereas *N. sedimenticola* produces globose to subglobose conidia measuring 1.0–1.5 × 1.0–1.5 µm.

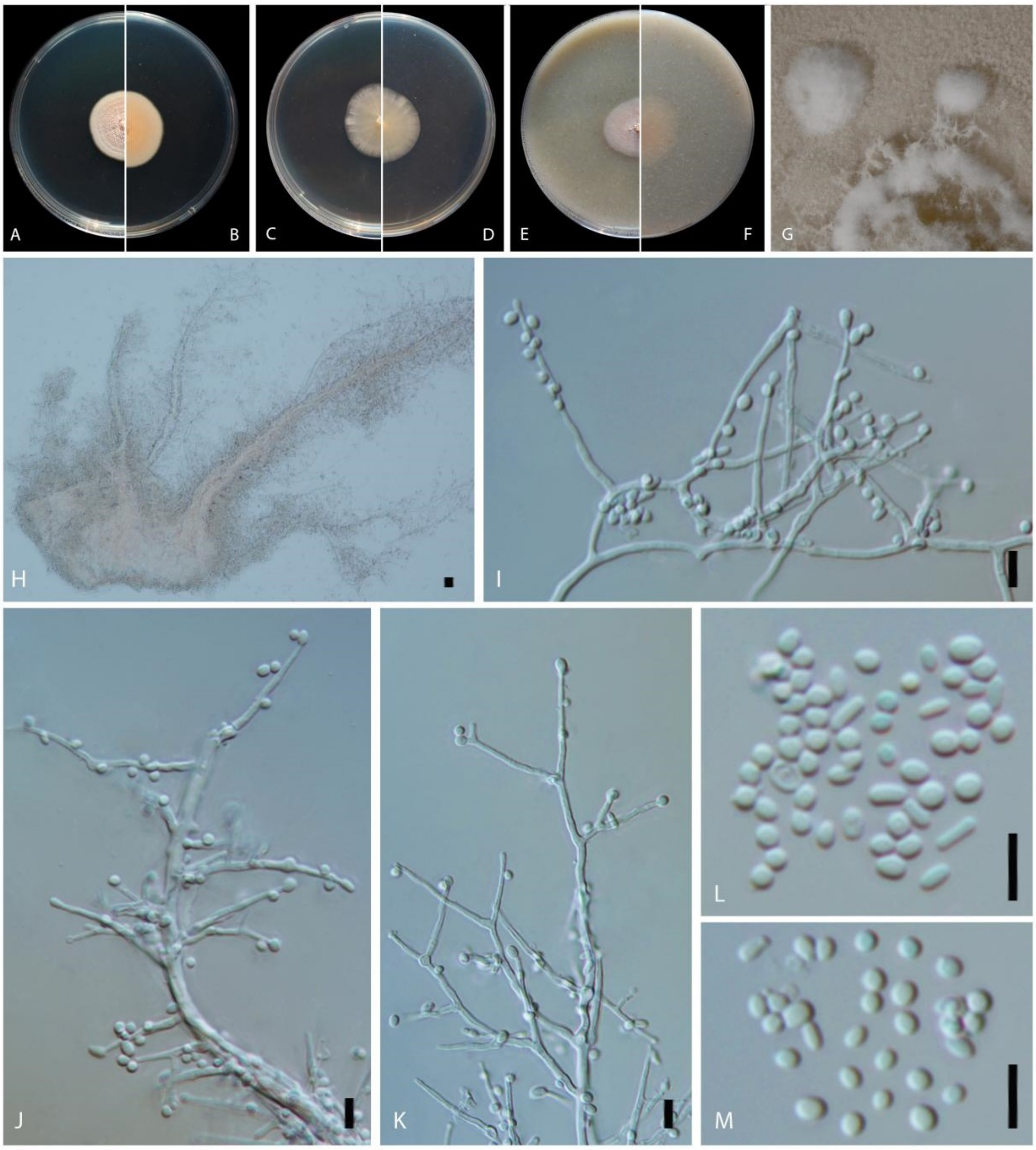

Fig. SD-20 ***Nothoacremoniopsis irregularis*** **(from ex-holotype CGMCC 3.22386)**. **A–F** Surface and reverse of colony on PDA, MEA and OA. **G** Sporulation on PDA. **H–K** Conidiophores and conidiogenous cells. **L–M** Conidia. Scale bars: **H–M** = 5 µm.

Nothoacremoniopsis sedimenticola M. Li, M. Raza & L. Cai, ***sp. nov.***

Fungal Names: FN571513; Fig. SD-21

Etymology: Referring to the substrate in which this fungus was isolated.

Hyphae hyaline, branched, smooth or rough walled, septate, thin-walled, 1.0–1.5 µm wide. **Asexual morph** *Conidiomata* superficial, bowl-shaped, pale yellow, normally surrounded by hyphal hairs. *Conidiophores* simple or branched, emerging laterally or terminally from vegetative hyphae or ropes of hyphae, straight or flexuous.

Conidiogenous cells phialidic, solitary, cylindrical to subulate, smooth- and thin-walled, hyaline, sympodially proliferating polyphialides, 14–27 μm long, 1.0–1.5 μm wide at the base, tapering towards the apex. *Conidia* unicellular, smooth- and thin-walled, hyaline, globose or subglobose, 1.0–1.5 × 1.0–1.5 μm (av. = 1.5 ± 0.19 × 1.2 ± 0.1 μm, n = 30), arranged in heads. *Chlamydospores* not observed. **Sexual morph** not observed.

Culture characteristics—Colonies on PDA attaining 17–23 mm diam. after 3 weeks, flat, felty, margin slightly undulate, white, aerial mycelia sparse. Reverse white. Sporulation within 3 weeks on PDA. Colonies on MEA attaining 23–25 mm diam. after 3 weeks, flat, annular, margin entire, white, aerial mycelia extremely sparse. Reverse white. Colonies on OA attaining 13–16 mm diam. after 3 weeks, flat, felty, margin entire, white to pale grey, aerial mycelia extremely sparse. Reverse white.

Material examined: CHINA, Guangdong, National Mangrove Nature Reserve of Futian Shenzhen, isolated from sediment, September 2020, M. Li and Z. F. Zhang, HMAS 352437 (holotype designated here, dried culture), ex-type living culture CGMCC 3.22383 = LC15899; ibid., CGMCC 3.22385 = LC15900.

Notes: *Nothoacremoniopsis sedimenticola* is phylogenetically allied to *N. irregularis* (Fig. SD-19), but they can be easily distinguished (see notes of *N. irregularis*).

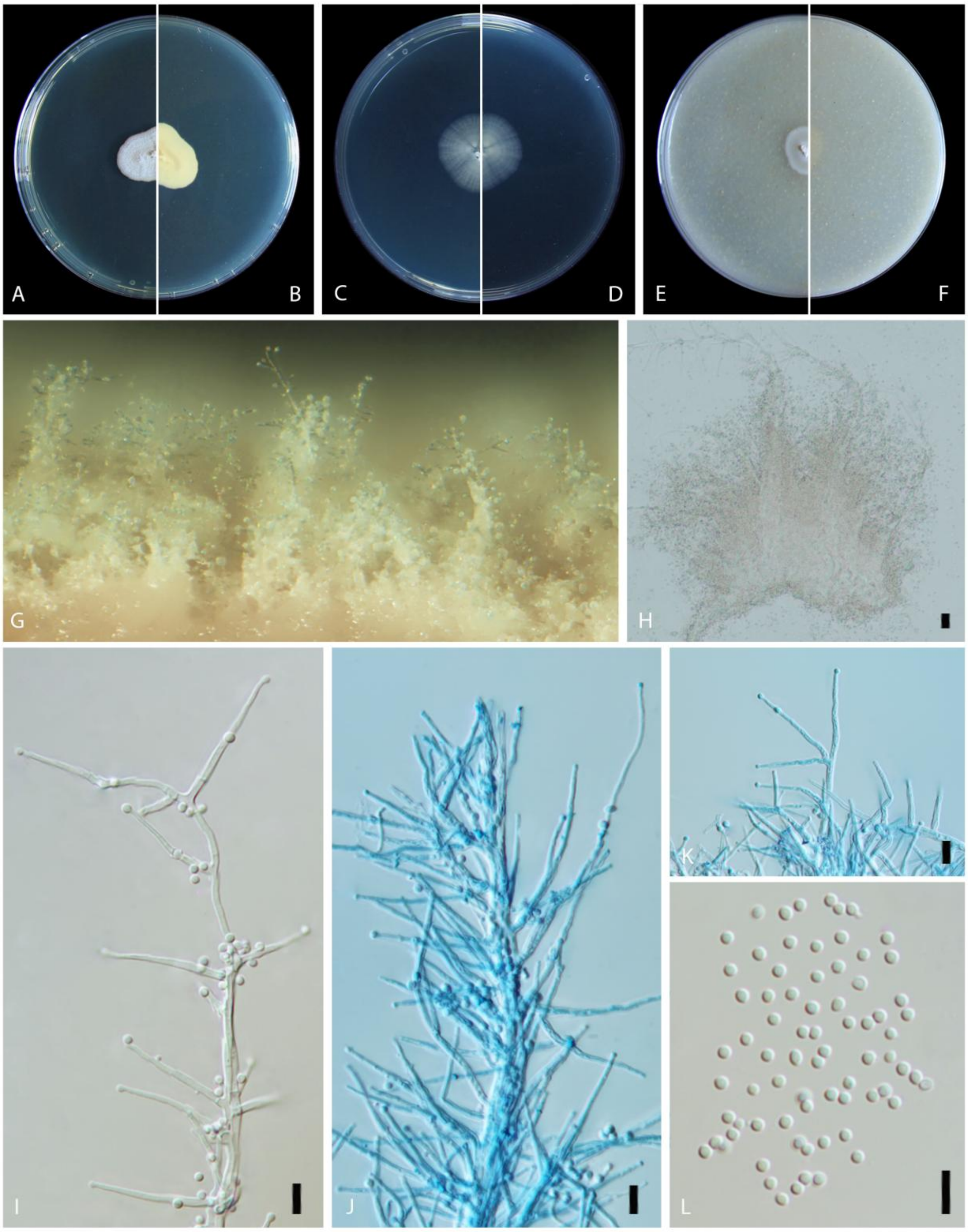

Fig. SD-21 ***Nothoacremoniopsis sedimenticola*** **(from ex-holotype CGMCC 3.22383)**. **A–F** Surface and reverse of colony on PDA, MEA and OA. **G** Sporulation on PDA. **H–K** Conidiophores and conidiogenous cells (**H–I** without staining cotton blue, **J–K** Staining with cotton blue). **L** Conidia. Scale bars: **H–L** = 5 μm.

Phaeocollarina M. Li, M. Raza & L. Cai, ***gen. nov.***

Fungal Names: FN571514

Etymology: Referring to the pale brown conidia of its type species and the close

phylogenetic relationship with the genus *Collarina.*

Asexual morph *Conidiophores* short and unbranched, arising from aerial or submerged hyphae, simple, erect, aseptate, often ending in single terminal phialide, smooth to verruculose, hyaline, becoming pale brown towards the tip. *Conidiogenous cells* phialidic, solitary, elongate-ampulliform and attenuated at the base, determinate, thin-walled, discrete, hyaline to pale brown. *Conidia* unicellular, hyaline to pale brown, aseptate, solitary, smooth- and thin-walled, guttulate, fusoid-ellipsoidal, straight, apex subobtuse, base truncate, arranged in heads. **Sexual morph** not observed.

Type: Phaeocollarina guttulata M. Li, M. Raza & L. Cai

Notes: *Phaeocollarina* is herein introduced to accommodate *P. guttulata*. Two strains representing *P. guttulata* clustered together in an independent clade closely related to *Collarina* (Fig. SD-19). Morphologically, two genera are different in the shape of conidiophores, conidiogenous cells and conidia (unbranched conidiophores, elongate-ampulliform conidiogenous cells and fusoid-ellipsoidal guttulate conidia in *Phaeocollarina*; branched or unbranched conidiophores, cylindrical with collarette conidiogenous cells and ellipsoidal to subglobose eguttulate conidia in *Collarina*) (Crous et al. 2014).

Phaeocollarina guttulata M. Li, M. Raza & L. Cai, ***sp. nov.***

Fungal Names: FN571515; Fig. SD-22

Etymology: Referring to the guttulate conidia of this species.

Hyphae hyaline to pale brown, septate, smooth, thin-walled, branched, single or in bundles, 1.0–2.5 µm wide. **Asexual morph** *Conidiophores* short and unbranched, arising from aerial or submerged hyphae, simple, erect, aseptate, often ending in single terminal phialide, smooth to verruculose, hyaline becoming pale brown towards the tip. *Conidiogenous cells* phialidic, solitary, elongate-ampulliform and attenuated at the base, determinate, thin walled, discrete, hyaline to pale brown, 7.0–13.5 × 1.0–1.5 µm. *Conidia* unicellular, hyaline to pale brown, aseptate, solitary, smooth- and thin-walled, guttulate, fusoid-ellipsoidal, straight, apex subobtuse, base truncate,

2.0–3.0 × 1.0–1.5 µm (av. = 2.6 ± 0.2 × 1.2 ± 0.11 µm, n = 30), arranged in heads. *Chlamydospores* not observed. **Sexual morph** not observed.

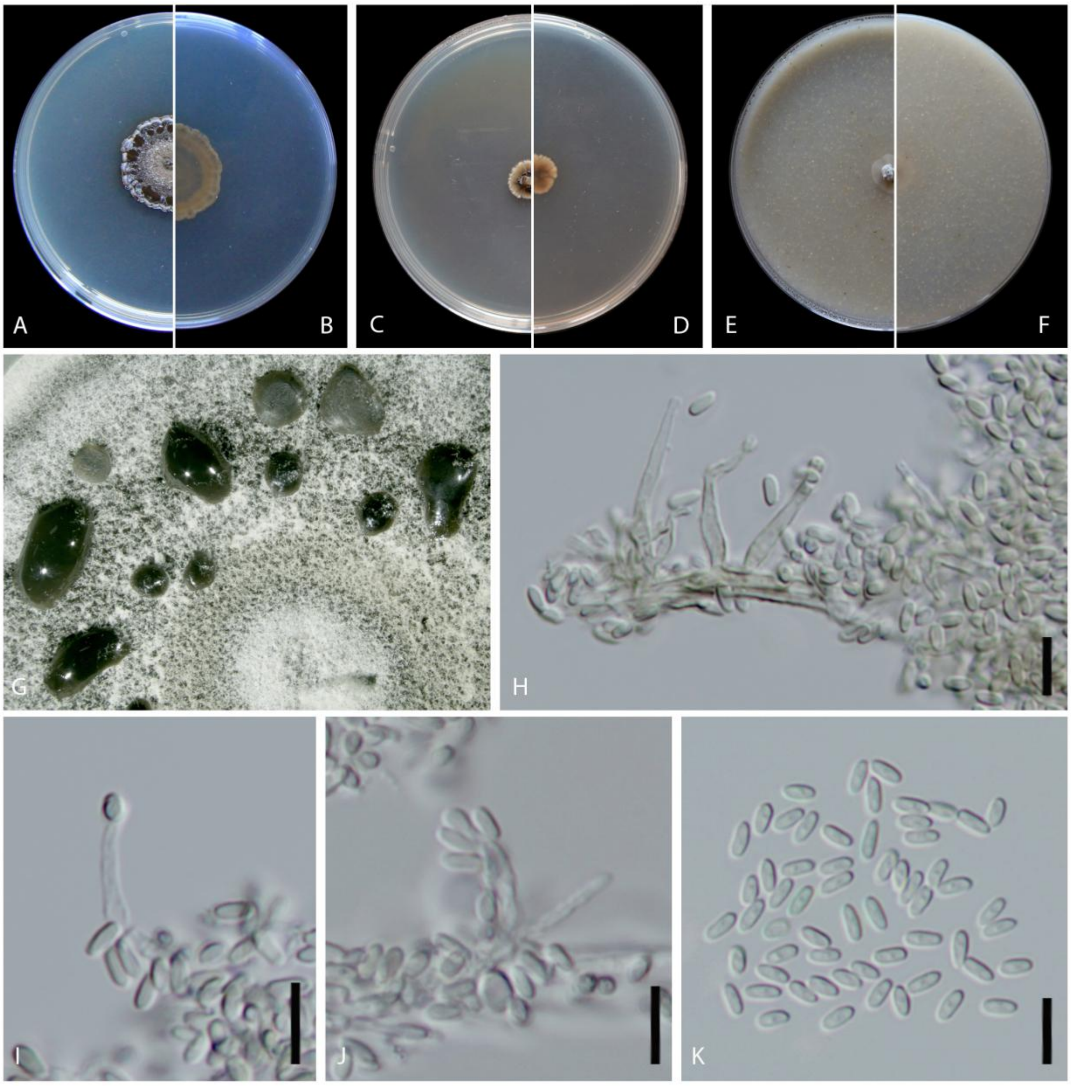

Fig. SD-22 *Phaeocollarina guttulata* (from ex-holotype CGMCC 3.22396). **A–F** Surface and reverse of colony on PDASS, MEA and OA. **G** Sporulation on PDASW. **H–J** Conidiophores and conidiogenous cells. **K** Conidia. Scale bars: **H–K** = 5 µm.

Culture characteristics—Colonies on PDASS attaining 35–37 mm diam. after 3 weeks, flat, felty to pulverulent, effuse, margin dentate, grey to dark brown, aerial mycelia sparse. Reverse yellowish brown to dark brown. Sporulation within 3 weeks on PDASS. Colonies on MEA attaining 11–12 mm diam. after 3 weeks, flat, felty to pulverulent, effuse, margin with fimbriate, white to dark brown, aerial mycelia sparse. Reverse white to dark brown. Colonies on OA attaining 10–12 mm diam. after 3

weeks, flat, margin undulate, white, aerial mycelia extremely sparse. Reverse white to pale brown.

Materials examined: CHINA, Guangdong, National Mangrove Nature Reserve of Futian Shenzhen, isolated from sediment, September 2020, M. Li and Z. F. Zhang, HMAS 352439 (holotype designated here, dried culture), ex-type living culture CGMCC 3.22396 = LC15906; ibid., CGMCC 3.22401 = LC15907.

Notes: The monotypic genus *Phaeocollarina* is represented by *P. guttulata*. Morphologically it is distinguished from the phylogenetically closely related species *C. aurantiaca* by producing unbranched conidiophores, whereas the latter produces branched conidiophores. Furthermore, *P. guttulata* differs in the shape and size of conidiogenous cells (elongate-ampulliform, 7.0–13.5 × 1.0–1.5 µm in *P. guttulata*; cylindrical with collarette, 10–40 × 1–1.5 µm in *C. aurantiaca*); their conidial shapes are also different (fusoid-ellipsoidal and guttulate in *P. guttulata*; ellipsoidal to sub-globose and eguttulate in *C. aurantiaca*) (Crous et al. 2014).

Bionectriaceae Samuels & Rossman, Stud. Mycol. 42: 15 (1999)

Bionectriaceae was established by Rossman et al. (1999) to accommodate *Bionectria* and allied genera. Species in this family are characterized by uniloculate hyaline to brightly coloured, soft perithecia with or without a well-developed stroma and unitunicate asci (Maharachchikumbura et al. 2016). Currently, 39 genera are accepted in *Bionectriaceae*.

Pseudosynnemellisia M. Li, M. Raza & L. Cai, **gen. nov.**

Fungal Names: FN571516

Etymology: Named after its morphological similarity to the genus *Synnemellisia*.

Asexual morph *Conidiophores* cylindrical, hyaline to slightly yellowish, septate, composed of main axis, sometime more major branches, with conidiogenous cells arising terminal or laterally. *Conidiogenous cells* hyaline, straight or slightly curved, terminal or lateral, ampulliform, tapering towards apex, sometime with conidial secession scar. *Conidia* unicellular, hyaline, aseptate, ellipsoidal, ovoid to clavate, with obtuse tip and truncated base. **Sexual morph** not observed.

Type: Pseudosynnemellisia favida M. Li, M. Raza & L. Cai

Notes: An unnamed species represented by strains CGMCC 3.22477 and CGMCC 3.22483 clustered in the family *Bionectriaceae* but could not be accommodated in any known genera (Fig. SD-19). It forms a sister clade to the genus *Synnemellisia.* Morphologically, it could be differentiated from *Synnemellisia* species in producing branched conidiophores, ampulliform conidiogenous cells and ellipsoidal, ovoid to clavate conidia. Therefore a new genus is proposed to accommodate this distinct taxon.

Pseudosynnemellisia favida M. Li, M. Raza & L. Cai, ***sp. nov.***

Fungal Names: FN571517; Fig. SD-23

Etymology: Referring to the color of its conidiomata, pale yellow.

Hyphae branched, septate, hyaline, smooth, thin walled, anastomosis, 1.0–2.0 µm wide. **Asexual morph** *Conidiomata* whitish to pale yellow, irregular in shape, aggregated. *Conidiophores* cylindrical, hyaline to slightly yellowish, septate, composed of main axis, sometime more major branches, with condiogenous cells arising terminal or laterally. *Conidiogenous cells* hyaline, straight or curved, terminal or lateral, ampulliform, tapering towards apex, sometime with conidial secession scar, 5.5–13 µm long, 1.5–2.5 µm wide at base. *Conidia* unicellular, hyaline, aseptate, ellipsoidal, ovoid to clavate, 2.0–4.5 × 1.5–2.5 µm (av. = 3.5 ± 0.68 × 1.9 ± 0.19 µm, n = 30), with obtuse tip and truncated base. **Sexual morph** not observed.

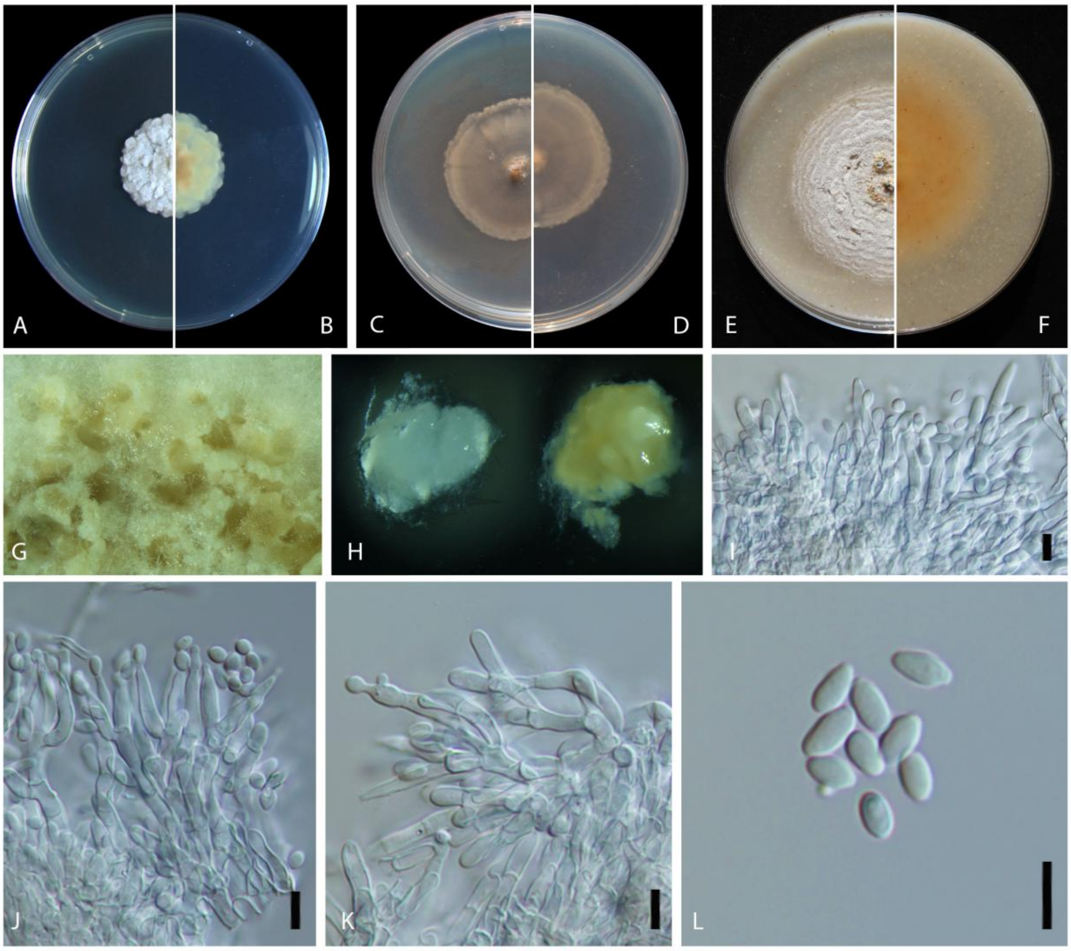

Fig. SD-23 *Pseudosynnemellisia favida* (from ex-holotype CGMCC 3.22477). **A–F** Surface and reverse of colony on PDA, MEA, and OA. **G** Sporulation on OA. **H** Conidiomata (immature and mature). **I–K** Conidiophores and conidiogenous cells. **L** Conidia. Scale bars: **I–L** = 5 µm.

Culture characteristics—Colonies on PDA attaining 29–30 mm diam. after 3 weeks, flat, felty, annular, margin undulate, white, aerial mycelia sparse. Reverse white to yellowish white. Colonies on MEA attaining 38–41 mm diam. after 3 weeks, flat, margin dentate, white to pale orange, aerial mycelia extremely sparse. Reverse white to pale orange. Colonies on OA attaining 50–53 mm diam. after 3 weeks, flat, felty to pulverulent, annular, margin undulate, white to apricot, aerial mycelia sparse. Reverse white to apricot. Sporulation within 3 weeks on OA.

Material examined: CHINA, Guangdong, National Mangrove Nature Reserve of Futian Shenzhen, isolated from sediment, September 2020, M. Li and Z. F. Zhang, HMAS 352448 (holotype designated here, dried culture), ex-type living culture

CGMCC 3.22477 = LC15930; ibid., CGMCC 3.22483 = LC15931.

Notes: Phylogenetically, *Pseudosynnemellisia favida* represents a well-supported distinct lineage in *Bionectriaceae* (Fig. SD-19). The new species *P. favida* is most closely related to the species of *Synnemellisia* (Crous et al. 2016; Rao et al. 1988). However, *P. favida* is distinguishable from *Synnemellisia* by producing branched conidiophores, whereas which are unbranched in the latter. Meanwhile, *P. favida* produces whitish to pale yellow conidiomata, which were not observed in *Synnemellisia*. In addition, the shape and size of conidiogenous cells are differentiated (ampulliform, 5.5–13 × 1.5–2.5 µm in *P. favida*; cylindrical and fusiform, 14–57 × 2–5 µm in *Synnemellisia*). Moreover, *P. favida* can be significantly distinguished from *Synnemellisia* by the shape and the size of its conidia, being ellipsoidal, ovoid to clavate measuring 2.0–4.5 × 1.5–2.5 µm in *P. favida*, whereas species of *Synnemellisia* produces navicular to fusiform conidia measuring 20–52 × 4–9 µm.

Sedecimiellaceae M. Li, M. Raza & L. Cai, ***fam. nov.***

Fungal Names: FN571518

Asexual morph *Conidiophores* unbranched, straight or flexuous. *Conidiogenous cells* phialidic, cylindrical, acicular to subulate, sometimes with a slightly flexuose apex, unbranched, thin- and smooth-walled, sometimes rugose towards the base, hyaline. *Conidia* smooth- and thin-walled, hyaline, ellipsoidal, ovoid to spherical, arranged in heads. **Sexual morph** *Ascomata* orange to dark brown, pyriform with globose to subglobose venter, immersed, erumpent or exposed, coriaceous, ostiolate, smooth. *Neck* thick, inner wall extends into upper part of ascoma centrum. *Periphyses* present, short, non-septate. *Peridium* orange to dark brown, two-layered. *Asci* unitunicate, cylindrical, short pedunculate, thin-walled, no apical pore, 16-spored, persistent, developing from inner wall of ascoma base. *Paraphyses* present, irregular, often tapering towards apex, branched, septate. *Ascospores* globose, hyaline, smooth, thin-walled, without appendage or sheath.

Type: Sedecimiella K.L. Pang, Alias & E.B.G. Jones

Notes: As above, *Sedecimiella* and *Heteroacremonium* constitute the new family

Sedecimiellaceae (Fig. SD-19). In our phylogenetic tree (Fig. SD-19), *Sedecimiella* is represented by five species including three new species *S. alba, S. funiculosus* and *S. subulata* and one new combination *S. minutispora* which are described and proposed below.

Sedecimiella K.L. Pang, Alias & E.B.G. Jones, in Pang, Alias, Chiang, Vrijmoed & Jones, Bot. Mar. 53(6): 495 (2010)

Asexual morph *Conidiophores* emerging laterally or terminally from vegetative hyphae or ropes of hyphae, straight or flexuous. *Conidiogenous cells* phialidic, acicular to subulate, sometimes with a slightly flexuose apex, unbranched, thin- and smooth-walled, hyaline. *Conidia* unicellular, smooth- and thin-walled, hyaline, ellipsoidal, ovoid to spherical, arranged in heads. **Sexual morph** *Ascomata* orange to dark brown, pyriform with globose to subglobose venter, immersed, erumpent or exposed, coriaceous, ostiolate, smooth, not collapsing upon drying, KOH-, lactic acid-. *Neck* thick, inner wall extends into upper part of ascoma centrum. *Periphyses* present, short, non-septate. *Peridium* orange to dark brown, two-layered, outer stratum of thick-walled cells forming textura angularis, inner stratum of elongated, hyaline thin-walled cells. *Asci* unitunicate, cylindrical, short pedunculate, thin-walled, no apical pore, 16-spored, persistent, developing from inner wall of ascoma base. *Paraphyses* present, irregular, often tapering towards apex, branched, septate. *Ascospores* globose, one-celled, hyaline, smooth, thin-walled, without appendage or sheath (Pang et al. 2010).

Type: Sedecimiella taiwanensis K.L. Pang, Alias & E.B.G. Jones

Notes: In our phylogenetic tree, five species representing the genus *Sedecimiella* clustered within the new family *Sedecimiellaceae* (Fig. SD-19), and they formed a distinct clade sister to the new genus *Heteroacremonium.* Hence, we introduce three new species *S. alba*, *S. funiculosus* and *S. subulata*, and proposed one new combination *S. minutispora*.

Sedecimiella alba M. Li, M. Raza & L. Cai, ***sp. nov.***

Fungal Names: FN571519; Fig. SD-24

Etymology: Referring to the color of its white colonies on plates.

Hyphae septate, hyaline, branched, smooth, thin-walled, 1.0–2.5 μm wide. **Asexual morph** *Conidiophores* emerging laterally or terminally from vegetative hyphae or ropes of hyphae, straight or flexuous. *Conidiogenous cells* phialidic, cylindrical to subulate, attenuated at the base, occasionally branched, thin- and smooth-walled, variable in length 6.0–17 μm long, 0.5–1.0 μm at the base. *Conidia* hyaline, solitary, aseptate, rough at initially, smooth with age, subglobose to obovoid, 1.0–2.0 × 1.0–1.5 μm (av. = 1.7 ± 0.1 × 1.3 ± 0.1 μm, n = 30), arranged in heads. *Chlamydospores* terminal, globose, thick-walled, 2.0–3.5 × 2.0–3.0 μm. **Sexual morph** not observed.

Culture characteristics—Colonies on PDA attaining 21–24 mm diam. after 3 weeks, coriarious, plicate and crack, margin dentate, white, aerial mycelia extremely sparse. Reverse white. Sporulation within 3 weeks on PDA. Colonies on MEA attaining 37–40 mm diam. after 3 weeks, flat, felty to pulverulent, margin dentate, white, aerial mycelia extremely sparse. Reverse white. Colonies on OA attaining 55–57 mm diam. after 3 weeks, flat, felty to pulverulent, margin entire, white, aerial mycelia extremely sparse. Reverse white.

Material examined: CHINA, Guangdong, National Mangrove Nature Reserve of Futian Shenzhen, isolated from sediment, September 2020, M. Li and Z. F. Zhang, HMAS 352440 (holotype designated here, dried culture), ex-type living culture CGMCC 3.22402 = LC15908; ibid., CGMCC 3.22404 = LC15909.

Notes: Phylogenetically, *Sedecimiella alba* is most closely related to *S. minutispora* and *S. taiwanensis* (Fig. SD-19), but is distinguishable from *S. minutispora* in the narrower conidiogenous cells (0.5–1.0 μm *vs*. 1–1.5 μm). In addition, conidia from *S. alba* are wider than that of *S. minutispora* at short axis (1.0–1.5 μm *vs*. 0.6–0.8 μm) (Gams 1971). Furthermore, *Sedecimiella alba* only produces asexual morph while in contrast *S. taiwanensis* only produces sexual morph.

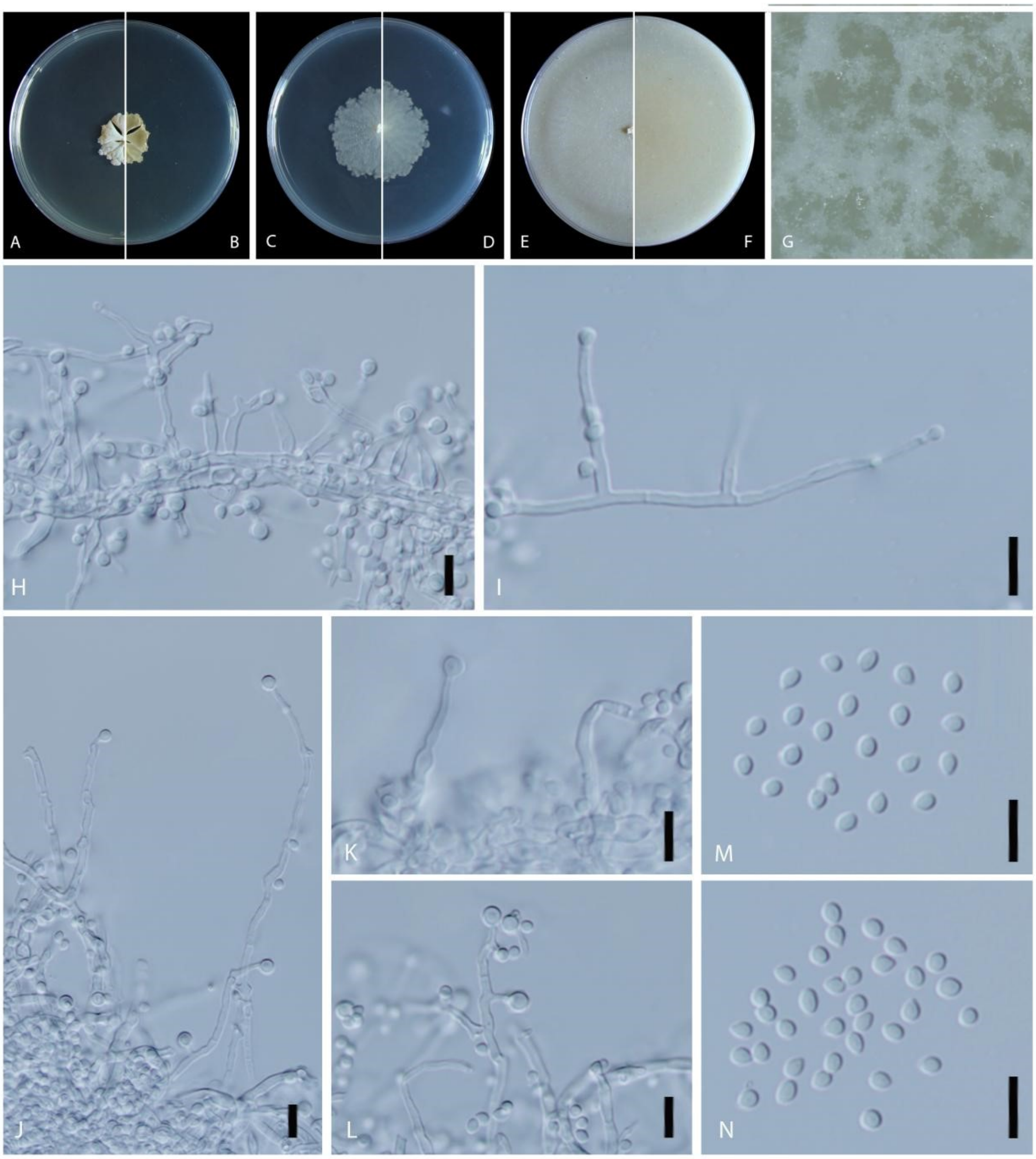

Fig. SD-24 ***Sedecimiella alba*** **(from ex-holotype CGMCC 3.22402)**. **A–F** Surface and reverse of colony on PDA, MEA and OA. **G** Sporulation on PDA. **H–K** Conidiophores and conidiogenous cells. **L** Chlamydospores. **M–N** Conidia. Scale bars: **H–L** = 5 µm.

Sedecimiella funiculosus M. Li & L. Cai, ***sp. nov.***

Fungal Names: FN571520; Fig. SD-25

Etymology: Referring to the funiculose mycelium of this species.

Hyphae hyaline, septate, smooth, branched, 1.0-1.5 µm wide. **Asexual morph** *Conidiophores* emerging laterally or terminally from vegetative hyphae or ropes of hyphae, straight or flexuous. *Conidiogenous cells* phialidic, cylindrical to subulate,

unbranched, thin- and smooth-walled, hyaline, 6.0–28 µm long, 1.0–1.5 µm wide at the base. *Conidia* unicellular, smooth- and thin-walled, hyaline, ellipsoidal, ovoid to spherical, 1.0–2.0 × 1.0–1.5 µm (av. = 1.8 ± 0.21 × 1.3 ± 0.09 µm, n = 30), arranged in heads. *Chlamydospores* not observed. **Sexual morph** not observed.

Culture characteristics—Colonies after 2 weeks at 25 °C, on PDA reaching 53–54 mm diam, flat, felty to pulverulent, margin entire, white, aerial mycelium sparse. Reverse white. Sporulation within 2 weeks on PDA. On MEA reaching 46–49 mm diam, flat, felty to pulverulent, margin entire, white, aerial mycelium sparse. Reverse white. On OA reaching 44–46 mm diam, flat, felty to pulverulent, margin entire, white, aerial mycelium sparse. Reverse white.

Material examined: CHINA, Guangdong, National Mangrove Nature Reserve of Futian Shenzhen, isolated from sediment, September 2020, M. Li and Z. F. Zhang, HMAS 352434 (holotype designated here, dried culture), ex-type living culture CGMCC 3.22348 = LC15891; ibid., CGMCC 3.22356 = LC15892.

Notes: *Sedecimiella funiculosus* clustered in a well-supported clade closely related to *S. alba* and *S. subulata* (Fig. SD-19). *Sedecimiella funiculosus* can be differentiated from *S. alba* by its longer and unbranched conidiogenous cell (6.0–28 µm in *S. funiculosus*; 6–17 µm in *S. alba*). In addition, *S. funiculosus* can be easily distinguished from *S. funiculosus* (see notes of *S. subulata*).

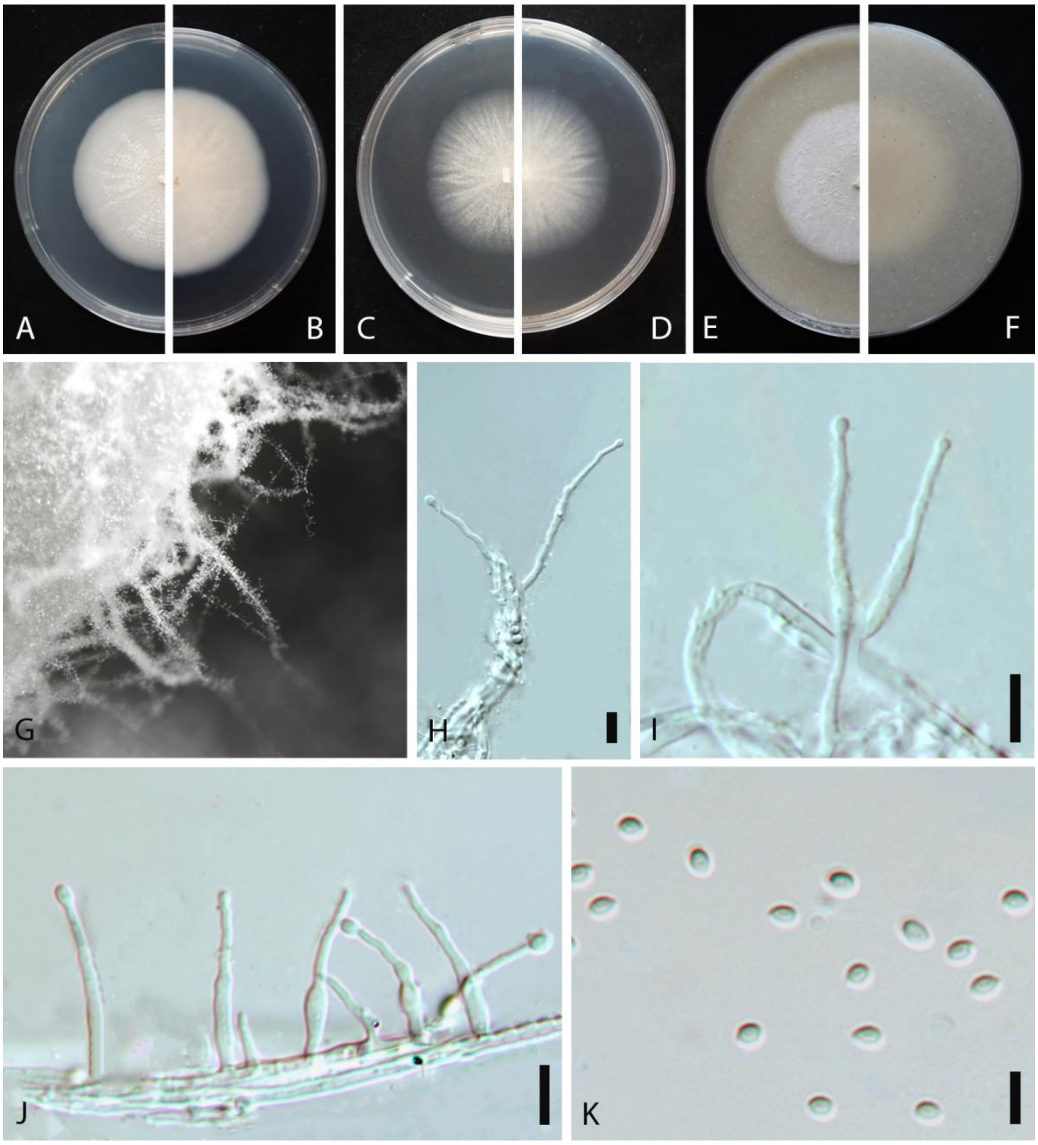

Fig. SD-25 ***Sedecimiella funiculosus*** **(from ex-holotype CGMCC 3.22348)**. **A–F** Surface and reverse of colony on PDA, MEA and OA. **G** Sporulation on PDA. **H–J** Conidiophores and conidiogenous cells. **K** Conidia. Scale bars: **H–L** = 5 µm.

Sedecimiella minutispora (Sukapure & Thirum.) M. Li & L. Cai, ***comb. nov.***

Fungal Names: FN571521

Basionym: *Cephalosporium minutisporum* Sukapure & Thirum., Mycologia 55: 566 (1963).

Synonym: *Acremonium minutisporum* H.C. Evans, Nova Hedwigia 73 (1–2): 46 (2001).

Holotype: India, Maharashtra, on soil, HACC 108, ex-type living culture, CBS 147.62.

Notes: This species was originally described by Sukapure & Thirumalachar (1963) as *Cephalosporium minutisporum*, and subsequently transferred to *Acremonium* based on morphology (Gams 1971). In the present study, the species well clustered in the *Sedecimiella* clade in the multi-locus phylogeny (Fig. SD-19). Hence, we proposed a new combination for this taxon as *Sedecimiella minutispora*.

Sedecimiella subulata M. Li & L. Cai, ***sp. nov.***

Fungal Names: FN571522; Fig. SD-26

Etymology: Referring to the subulate conidiogenous cells of this species.

Hyphae hyaline, septate, smooth, branched, 1.5–2.0 µm wide. **Asexual morph** *Conidiophores* emerging laterally or terminally from vegetative hyphae or ropes of hyphae, straight or flexuous. *Conidiogenous cells* phialidic, acicular to subulate, sometimes with a slightly flexuose apex, thin- and smooth-walled, hyaline, 9.0–16 µm long, 1.0–2.0 µm wide at the base. *Conidia* unicellular, smooth- and thin-walled, hyaline, ellipsoidal, ovoid to spherical, 1.5–2.0 × 1.0–1.5 µm (av. = 1.7 ± 0.19 × 1.4 ± 0.1 µm, n = 50), arranged in heads. *Chlamydospores* not observed. **Sexual morph** not observed.

Culture characteristics—Colonies after 2 weeks at 25 °C, on PDA reaching 37–39 mm diam, flat, floccose, margin slightly undulate, white, aerial mycelium sparse. Reverse white. On MEA reaching 52–53 mm diam, flat, felty, margin irregular, white, aerial mycelium sparse. Reverse white. Sporulation within 2 weeks on MEA. On OA reaching 49–50 mm diam, flat, felty, margin entire, white, aerial mycelium extremely sparse. Reverse white.

Material examined: CHINA, Guangdong, National Mangrove Nature Reserve of Futian Shenzhen, isolated from sediment, September 2020, M. Li and Z. F. Zhang, HMAS 352431 (holotype designated here, dried culture), ex-type living culture CGMCC 3.22330 = LC15885; ibid., CGMCC 3.22332 = LC15886.

Notes: *Sedecimiella subulata* clustered in a well-supported clade (Fig. SD-19) closely related to *S. funiculosus*, another novel species described in this study. *S. subulata* differs in the shorter conidiogenous cell (9.0–16 µm in *S. subulata*; 6.0–28

µm in *S. funiculosus*). In addition, *S. funiculosus* produces cylindrical conidiogenous cells, which were not observed in *S. subulata*.

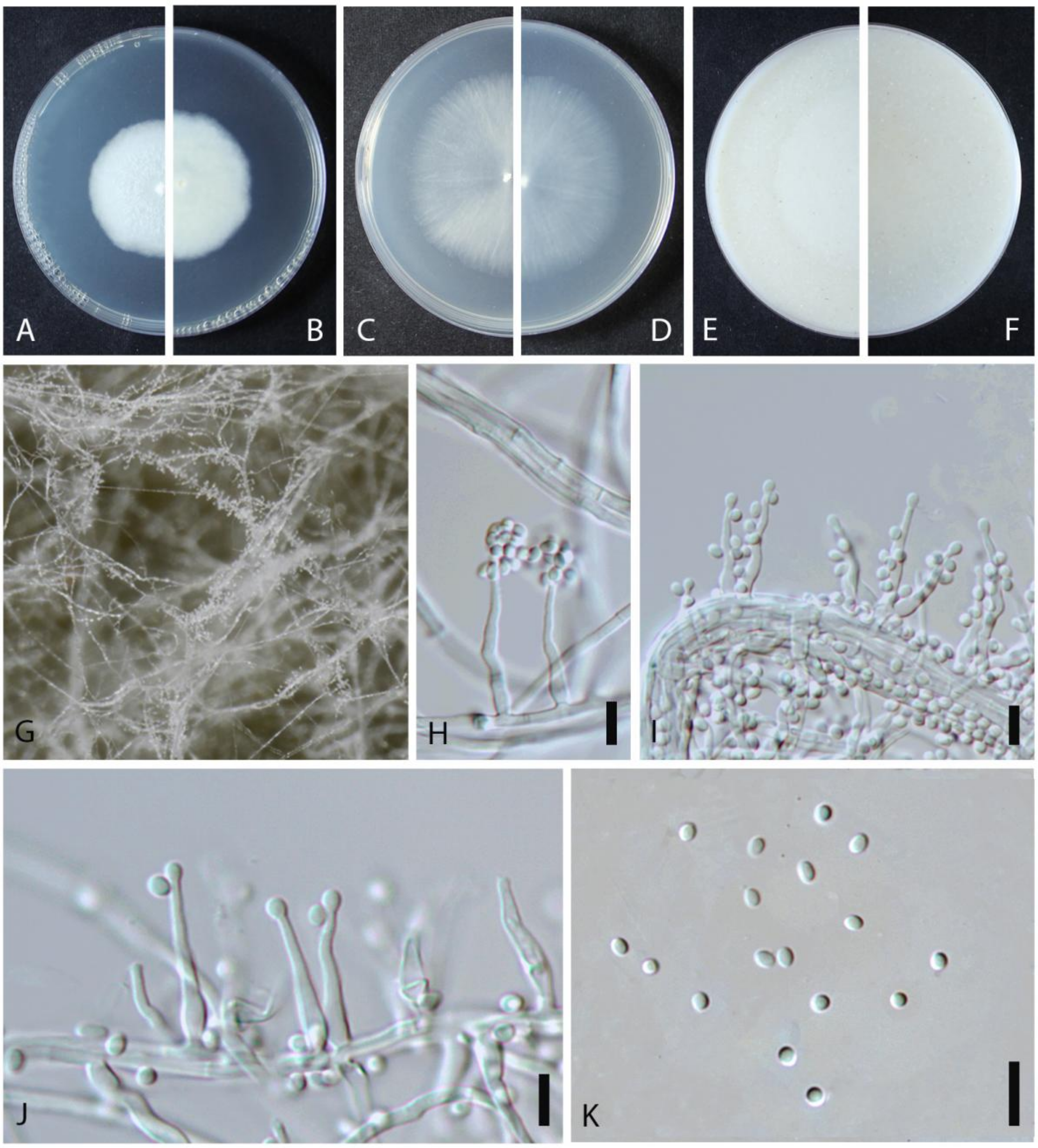

Fig. SD-26 *Sedecimiella subulata* (from ex-holotype CGMCC 3.22330). **A–F** Surface and reverse of colony on PDA, MEA and OA. **G** Sporulation on MEA. **H–J** Conidiophores and conidiogenous cells. **K** Conidia. Scale bars: **H–K** = 5 µm.

Heteroacremonium M. Li, M. Raza & L. Cai, ***gen. nov.***

Fungal Names: FN571523

Etymology: Named after its morphological similarity to the genus *Acremonium*.

Asexual morph *Conidiophores* simple or poorly branched, emerging laterally or terminally from vegetative hyphae or ropes of hyphae, straight or flexuous.

Conidiogenous cells phialidic, solitary, sympodially proliferating polyphialides, cylindrical to subulate, unbranched, discrete, hyaline. *Conidia* unicellular, smooth- and thin-walled, hyaline, globose or subglobose, guttulate, arranged in heads. **Sexual morph** not observed.

Type: Heteroacremonium rugosum M. Li, M. Raza & L. Cai

Notes: We herein establish a new genus *Heteroacremonium* to accommodate two new species named *H. album* and *H. rugosum*. In the multi-locus phylogenetic analysis, *Heteroacremonium* and *Sedecimiella* together constitute the newly established family *Sedecimiellaceae* (Fig. SD-19). Morphologically, *Heteroacremonium* differs from *Sedecimiella* in having simple or poorly branched conidiophores and sympodially proliferating polyphialides which were not observed in *Sedecimiella*.

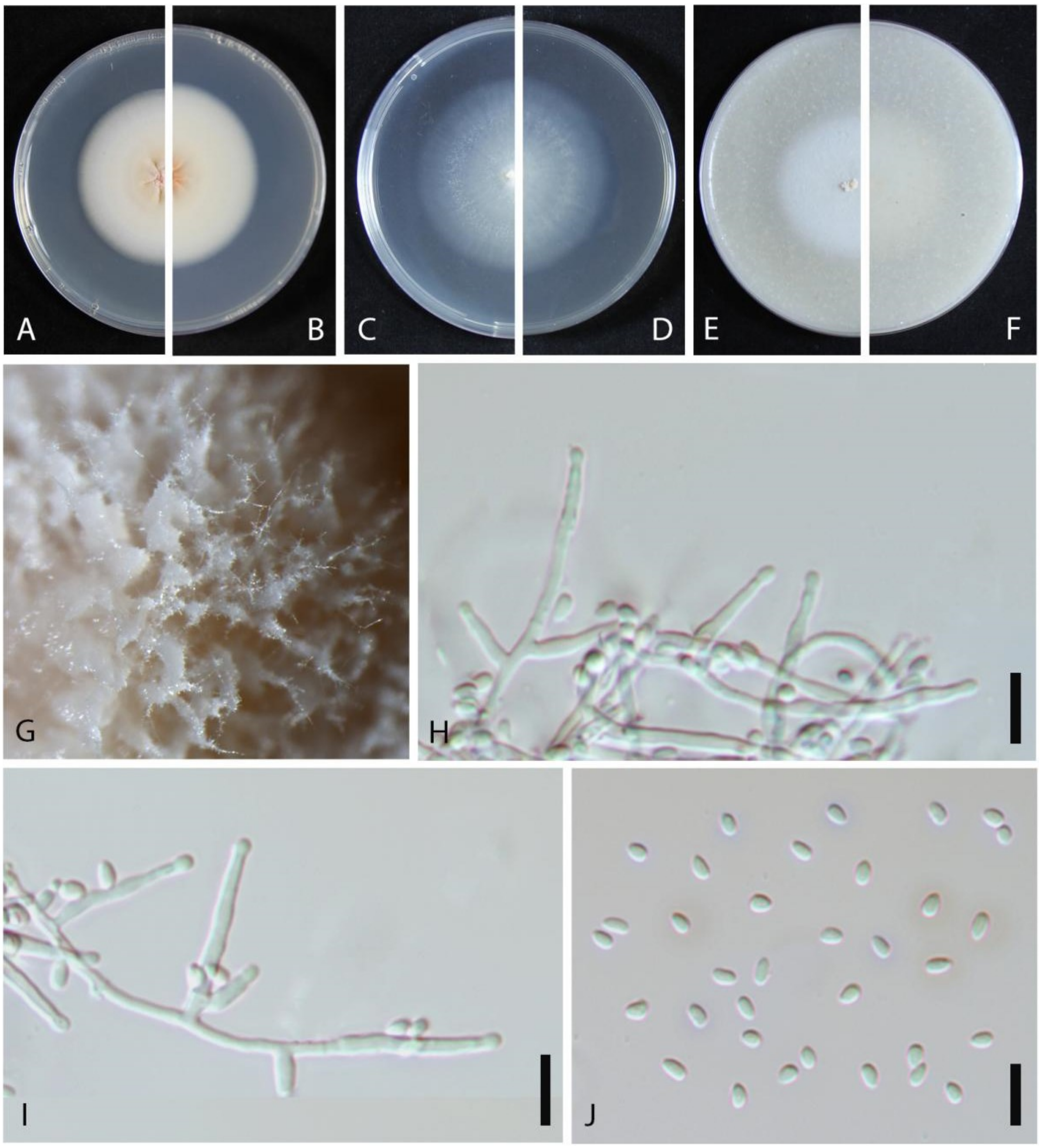

Fig. SD-27 ***Heteroacremonium album*** **(from ex-holotype CGMCC 3.22405)**. **A–F** Surface and reverse of colony on PDA, MEA and OA. **G** Sporulation on PDA. **H–I** Conidiophores and conidiogenous cells. **J** Conidia. Scale bars: **H–J** = 5 μm.

Heteroacremonium album M. Li & L. Cai, ***sp. nov.***

Fungal Names: FN571524; Fig. SD-27

Etymology: Referring to the color of its white colonies on OA medium.

Hyphae hyaline, septate, smooth, branched, 0.5–1.0 μm wide. **Asexual morph** *Conidiophores* simple or poorly branched, emerging laterally or terminally from vegetative hyphae, straight or flexuous. *Conidiogenous cells* phialidic, sympodially proliferating polyphialides, cylindrical, thin- and smooth-walled, hyaline, 4.5–12.0 μm long, 0.5–1.0 μm wide at the base. *Conidia* unicellular, smooth- and thin-walled,

hyaline, ellipsoidal to ovoid, 1.5–2.0 × 1.0–1.5 µm (av. = 1.8 ± 0.16 × 1.1 ± 0.11 µm, n = 30), arranged in heads. *Chlamydospores* not observed. **Sexual morph** not observed.

Culture characteristics—Colonies after 3 weeks at 25 °C, on PDA reaching 49–52 mm diam, flat, coriarious, plicate in the center, margin entire, white, paechpuff to pink near the center, aerial mycelium sparse. Reverse white to pink. Sporulation within 3 weeks on PDA. On MEA reaching 52–55 mm diam, flat, felty to pulverulent, margin entire, white, aerial mycelium sparse. Reverse white. On OA reaching 40–42 mm diam, flat, felty to pulverulent, margin entire, white, aerial mycelium extremely sparse. Reverse white.

Material examined: CHINA, Guangdong, National Mangrove Nature Reserve of Futian Shenzhen, isolated from sediment, September 2020, M. Li and Z. F. Zhang, M. Li, HMAS 352441 (holotype designated here, dried culture), ex-type living culture CGMCC 3.22405 = LC15910; ibid., CGMCC 3.22409 = LC15911.

Notes: *Heteroacremonium album* is phylogenetically allied to *H. rugosum* (Fig. SD-19), but they can be easily distinguished (see notes of *Heteroacremonium rugosum*).

Heteroacremonium rugosum M. Li, M. Raza & L. Cai, ***sp. nov.***

Fungal Names: FN571525; Fig. SD-28

Etymology: Referring to its rugose conidiogenous cells.

Hyphae branched, septate, hyaline, rough, thin-walled, 1.0-2.0 µm wide. **Asexual morph** *Conidiophores* simple or branched, emerging laterally or terminally from vegetative hyphae or ropes of hyphae, straight or flexuous. *Conidiogenous cells* phialidic, solitary, sympodially proliferating polyphialides, cylindrical to subulate, determinate, thin-walled and rugose towards the base, discrete, hyaline, 8.5–21.5 µm long, 0.5–1.5 µm wide at the base. *Conidia* unicellular, smooth- and thin-walled, hyaline, globose or subglobose, guttulate, 1.0–2.0 × 1.0–1.5 µm (av. = 1.5 ± 0.19 × 1.2 ± 0.1 µm, n = 30), arranged in heads. *Chlamydospores* terminal, globose, thick-walled, 2.1–3.3 × 2–3 µm. **Sexual morph** not observed.

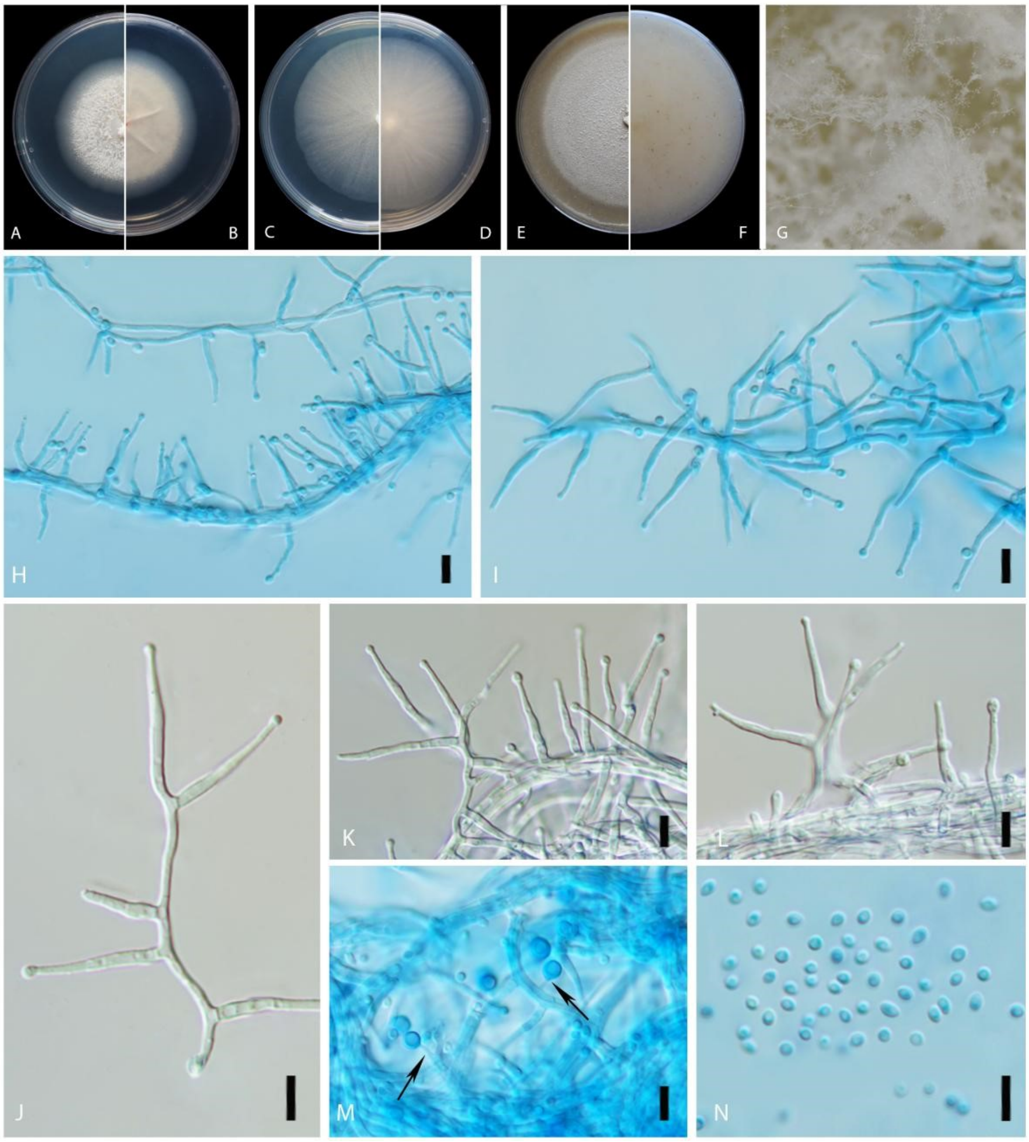

Fig. SD-28 *Heteroacremonium rugosum* (from ex-holotype CGMCC 3.22520). **A–F** Surface and reverse of colony on PDA, MEA and OA. **G** Sporulation on PDA. **H–L** Conidiophores and conidiogenous cells (**H–I** staining with cotton blue). **M** Chlamydospores. **N** Conidia stain with cotton blue. Scale bars: **H–N** = 5 μm.

Culture characteristics—Colonies on PDA attaining 57–60 mm diam. after 3 weeks, flat, felty, margin entire, white, aerial mycelia abundant. Reverse white. Sporulation within 3 weeks on PDA. Colonies on MEA attaining 67–69 mm diam. after 3 weeks, flat, felty, margin slightly undulate, white, aerial mycelia extremely sparse. Reverse white. Colonies on OA attaining 68–70 mm diam. after 3 weeks, flat, felty to pulverulent, margin entire, white, aerial mycelia sparse. Reverse white.

Material examined: CHINA, Guangdong, National Mangrove Nature Reserve of

Zhanjiang, isolated from sediment, November 2019, M. Li and J. E. Huang, HMAS 352452 (holotype designated here, dried culture), ex-type living culture CGMCC 3.22520 = LC15938; ibid., CGMCC 3.22522 = LC15939.

Notes: *Heteroacremonium rugosum* is closely related to *H. album*, another novel species described in this study (Fig. SD-19). Morphologically, *H. rugosum* differs from *H. album* in having rugose conidiogenous cells. Furthermore, *H. rugosum* produces globose to subglobose and guttulate conidia and chlamydospores, which were not observed in *H. album*.

Nectriaceae Tul. & C. Tul., Select. fung. carpol. (Paris) 3: 3 (1865)

The family *Nectriaceae* was first erected by Tulasne and Tulasne (1865) with *Nectria* as the type genus. But subsequently *Nectriaceae* was synonymized under *Hypocreaceae* (Miller 1949; von Arx 1954; Rogerson 1970; Barr 1990). *Nectriaceae* was recently reinstated and 66 genera were included in the family (Lumbsch and Huhndorf 2010; Maharachchikumbura et al. 2015; Lombard et al. 2015, Wijayawardene et al. 2018). Morphologically, *Nectriaceae* is characterized by uniloculate, pigmented ascomata and phialidic amerosporous to phragmosporous conidia (Lombard et al. 2015; Tribpromma et al. 2018).

Fusarium Link, Mag. Gesell. naturf. Freunde, Berlin 3(1-2): 10 (1809)

The genus *Fusarium* was introduced by Link (1809). *Fusarium* is a genus commonly found in nature and includes numerous important plant and human pathogens, as well as many industrial and commercially important species (da Silva Santos et al. 2020). Morphologically, *Fusarium* is characterized by falcate macroconidia and ovoid to reniform microconidia (Link 1809, Leslie and Summerell 2006). We herein introduce one novel species in *F. chlamydosporum* species complex (FCSC) based on morphology and sequence data analyses (Fig. SD-27).

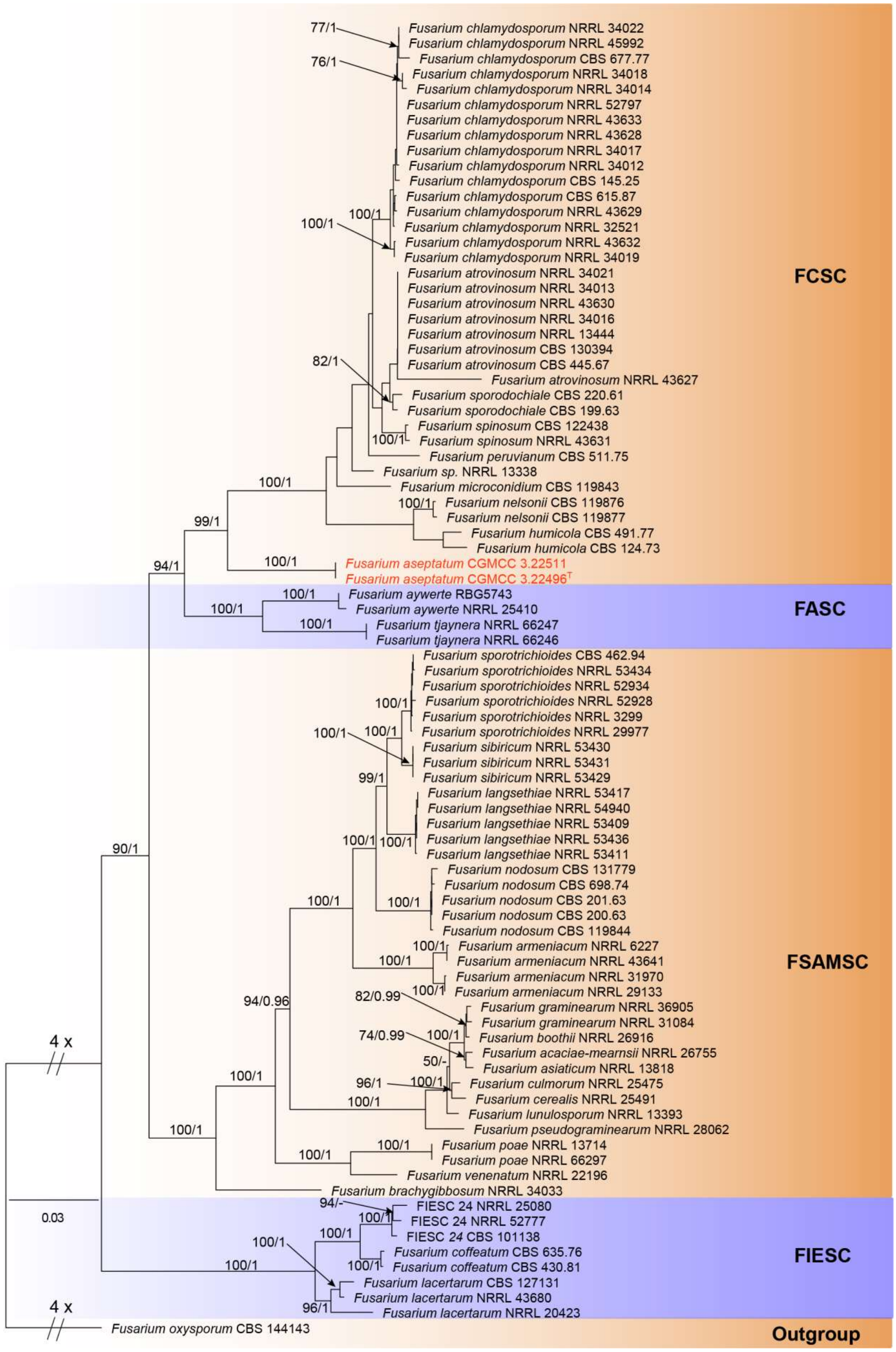

Fig. SD-29 Maximum likelihood tree of FCSC based on *cam*, *rpb1*, *rpb2* and *tef1* sequences. The RAxML BS above 50% and BPP above 0.90 are presented at the nodes BS/BPP. The tree was rooted to *Fusarium oxysporum* CBS 144143. Ex-type cultures are indicated with a letter "T" after

the accession number. The new species are printed in red font.

Fusarium aseptatum M. Li & L. Cai, ***sp. nov.***

Fungal Names: FN571526; Fig. SD-30

Etymology: Referring to the aseptate microconidia of the fungus.

Hyphae hyaline, septate, smooth, branched, 2.0–3.5 µm wide. **Asexual morph** *Sporodochia* not observed. *Conidiophores* carried on the aerial mycelium 7–25 µm tall, irregularly or sympodially branched or unbranched, bearing a lateral single phialide or terminal whorl of 2–4 phialides. *Aerial phialides* mono- and polyphialidic, subulate to subcylindrical, smooth- and thin-walled, 3.0–15.5 × 1.5–2.0 µm. *Aerial microconidia* forming small false heads on the tips of the phialides, hyaline,ellipsoidal, obovoid, fusiform, smooth- and thin-walled, aseptate, 3.5–8.0 × 2.0–3.0 µm (av. = 5.2 ± 0.91 × 2.2 ± 0.26 µm, n = 50). *Chlamydospores* not observed. **Sexual morph** not observed.

Culture characteristics—Colonies after 7 days at 25 °C, on PDA reaching 47–52 mm diam, floccose, raised at center, margin irregular, white to pink, aerial mycelium abundant. Reverse white to reddish orange. On OA reaching 55–60 mm diam, floccose, sunken at center, margin entire, white, aerial mycelium abundant. Reverse white to pale brown. On SNA reaching 36–40 mm diam, flat, floccose, margin slightly undulate, white to plum, aerial mycelium moderate abundant. Reverse white to plum.

Material examined: CHINA, Guangdong, National Mangrove Nature Reserve of Zhanjiang, isolated from sediment, November 2019, M. Li and J. E. Huang, HMAS 352450 (holotype designated here, dried culture), ex-type living culture CGMCC 3.22496 = LC15934; ibid., CGMCC 3.22511 = LC15935.

Notes: Based on the multi-locus phylogenetic analyses, *Fusarium aseptatum* forms an independent clade clearly separated from other species in *F. chlamydosporum* species complex (Fig. SD-29). Morphologically, *F. aseptatum* can be distinguished from known species by its aseptate microconidia (Lombard et al. 2019). Additionally, the phialides of *F. aseptatum* are narrower than other species

(1.5–2.0 μm *vs*. 2–6 μm).

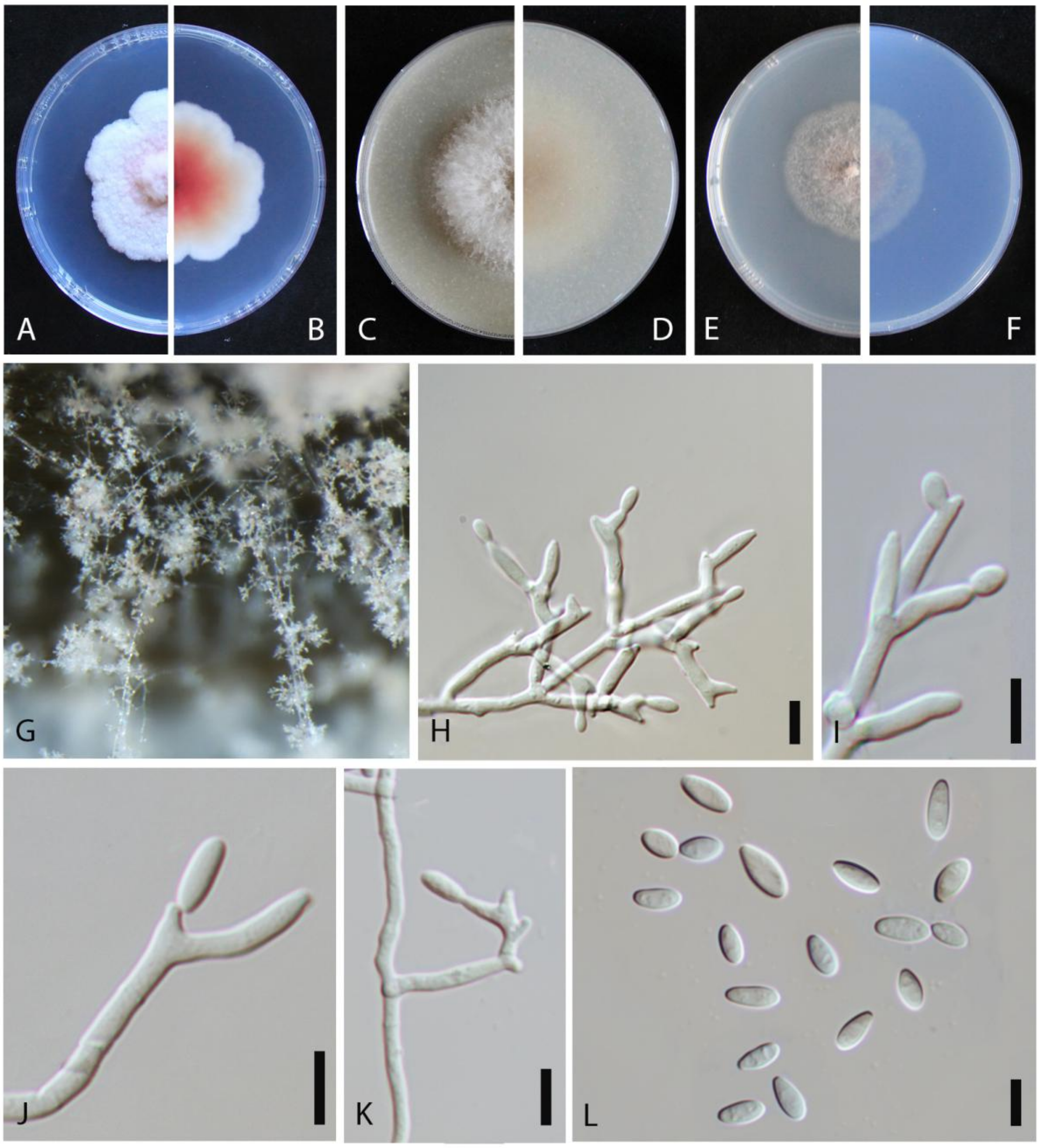

Fig. SD-30 ***Fusarium aseptatum*** **(from ex-holotype CGMCC 3.22496)**. **A–F** Surface and reverse of colony on PDA, OA and SNA. **G** Sporulation on SNA. **H–K** Conidiophores and conidiogenous cells. **L** Conidia. Scale bars: **H–L** = 5 μm.

Microascales Luttr. ex Benny & R.K. Benj., Mycotaxon 12(1): 40 (1980)

Halosphaeriaceae E. Müll. & Arx ex Kohlm., Can. J. Bot. 50: 1951 (1972)

Halosphaeriaceae, typified by *Halosphaeria,* is widely distributed in marine environments on different substrates, such as driftwood, mangrove wood, seagrasses and marine algae (Jones 2011; Jones and Pang 2012; Jones et al. 2013). Currently, 164 species classified in 7 genera are accepted in *Halosphaeriaceae* (Hyde et al. 2020).

Okeanomyces K.L. Pang & E.B.G. Jones, Bot. J. Linn. Soc. 146(2): 228 (2004)

The genus *Okeanomyces* was introduced by Pang et al. (2004) to accommodate single species *Okeanomyces cucullata*. The sexual morphology of *Okeanomyces* is characterized by subglobose or ellipsoidal ascomata, clavate asci, and cylindrical ascospores (Pang et al. 2004). Recently, Hyde et al. (2020) described the asexual morphology of a novel species *O. marinus* collected from an intertidal rocky shore in Thailand. Here, we introduce a new species *O. guttulatus* isolated from mangrove sediment (Fig. SD-31).

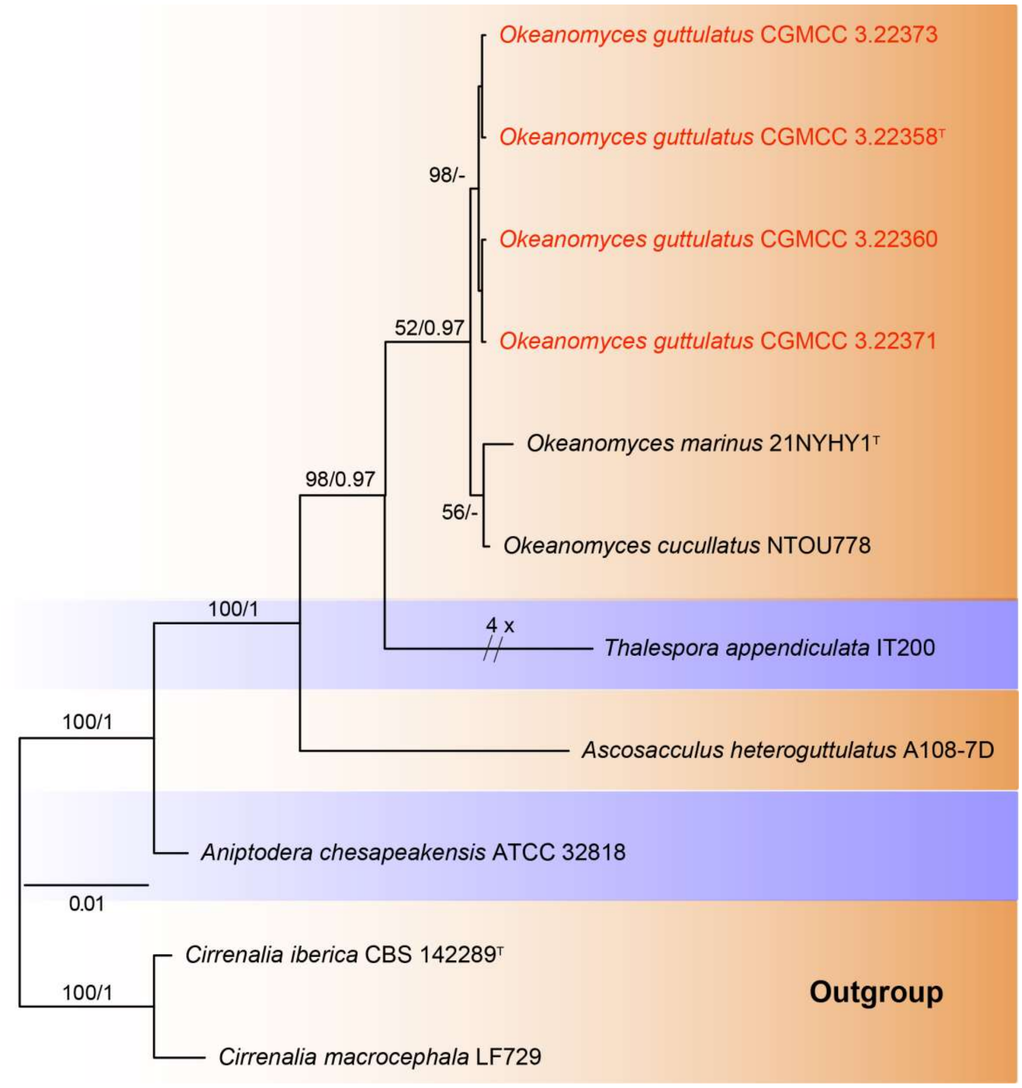

Fig. SD-31 Maximum likelihood tree of *Okeanomyces* based on ITS, LSU and SSU sequences. The RAxML BS above 50% and BPP above 0.90 are presented at the nodes BS/BPP. The tree was rooted to *Cirrenalia iberica* CBS 142289 and *Cirrenalia macrocephala* LF729. Ex-type cultures are indicated with a letter "T" after the accession number. The new species are printed in red font.

Okeanomyces guttulatus M. Li, M. Raza & L. Cai, ***sp. nov.***

Fungal Names: FN571527; Fig. SD-32

Etymology: Referring to the guttulate conidia of this species.

Hyphae thin walled, hyaline to pale brown, smooth, septate, branched, 2.0–2.5 µm wide. **Asexual morph** *Conidiophores* formed on aerial hyphae, slightly curved or straight, irregularly branched, sometimes two to five times branched, with conidia formed on terminal or lateral, 3.0–26 ×1.5–2.0 µm or longer. *Conidiogenous cells* hyaline to pale brown, cylindrical, 0–3-septate, constricted at septa, 2.8–8.8 ×1.3–2.6 µm. *Conidia* solitary, subglobose to globose, smooth- and thick-walled, pale to medium brown, 6.0–7.5 × 6.0–7.5 µm (av. = 6.6 ± 0.38 × 6.6 ± 0.38 µm, n = 30). **Sexual morph** not observed.

Culture characteristics—Colonies on PDA attaining 26–28 mm diam. after 3 weeks, flat, cottony, slightly raised at center, margin entire, grey to greyish green, aerial mycelia sparse. Reverse white to moss green. Colonies on MEA attaining 22–24 mm diam. after 3 weeks, flat, cottony, margin entire, grey to jade green, aerial mycelia sparse. Reverse jade green to dark brown. Sporulation within 3 weeks on MEA. Colonies on OA attaining 30–32 mm diam. after 3 weeks, flat, felty, margin slightly undulate, grey to black, aerial mycelia sparse. Reverse black.

Material examined: CHINA, Guangdong, National Mangrove Nature Reserve of Futian Shenzhen, isolated from sediment, September 2020, M. Li and Z. F. Zhang, HMAS 352435 (holotype designated here, dried culture), ex-type living culture CGMCC 3.22358 = LC15893; ibid., CGMCC 3.22360 = LC15894; ibid., CGMCC 3.22371 = LC15895; ibid., CGMCC 3.22373 = LC15896.

Notes: *Okeanomyces guttulatus* is phylogenetically closely related to *O. cucullatus* and *O. marinus* (Fig. SD-32). Morphologically, the conidia of *O. guttulatus* are much smaller than that of *O. marinus* (6.0–7.5 × 6.0–7.5 µm *vs*. 7–15 × 7–11 µm) (Hyde et al. 2020). Furthermore, *O. guttulatus* differs from *O. marinus* in the shape of conidia (subglobose to globose in *O. guttulatus*; oval to pyriform in *O. marinus*). Sexual morph has been observed in *O. cucullatus* but not yet in *O. guttulatus*.

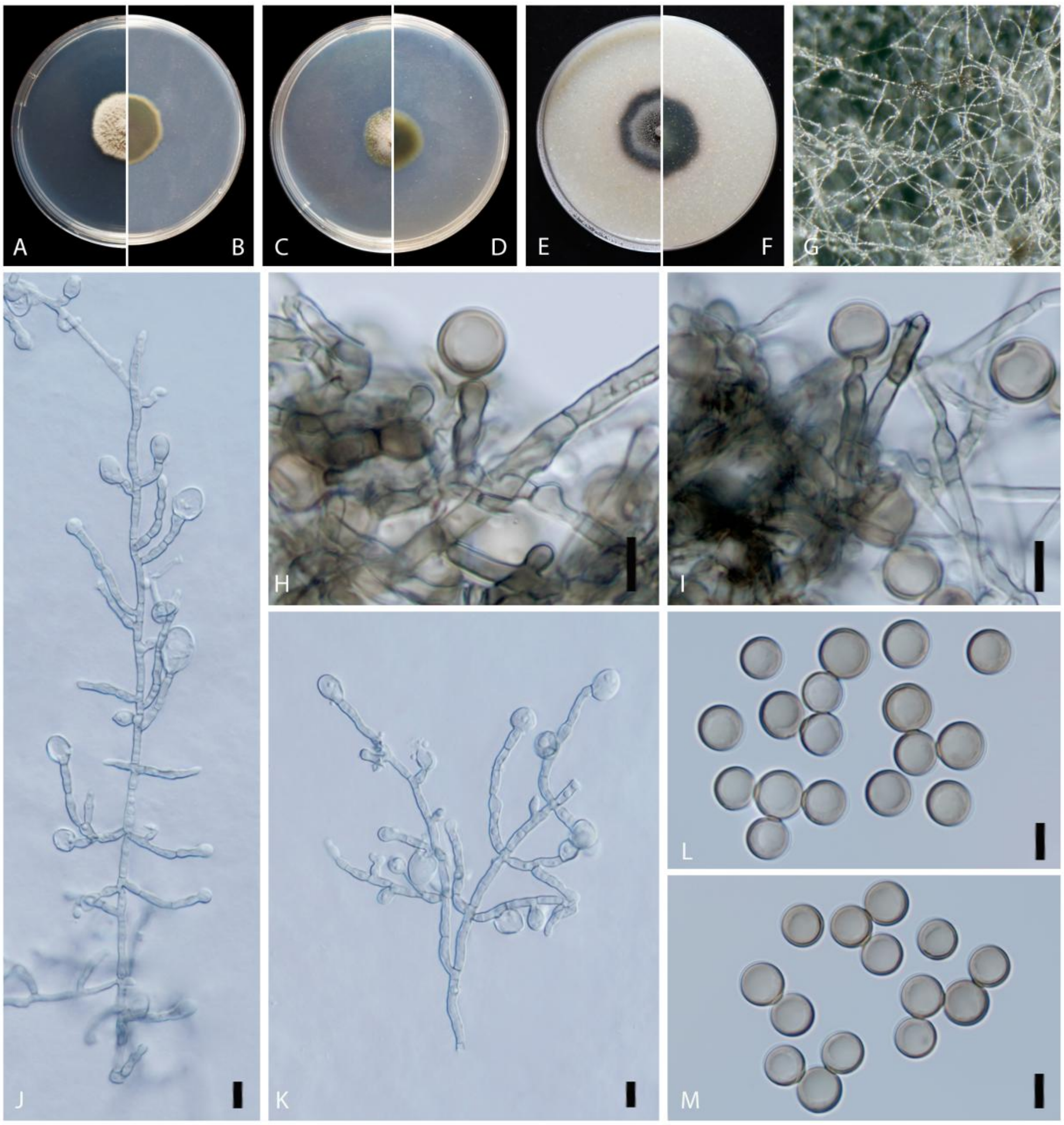

Fig. SD-32 ***Okeanomyces guttulatus*** **(from ex-holotype CGMCC 3.22358)**. **A–F** Surface and reverse of colony on PDA, MEA and OA. **G** Sporulation on MEA. **H–K** Conidiophores and conidiogenous cells. **L–M** Conidia. Scale bars: **H–M** = 5 μm.

Subclass Lulworthiomycetidae Dayar., E.B.G. Jones & K.D. Hyde, Fungal Diversity 72: 208 (2015)

Lulworthiales Kohlm., Spatafora & Volkm.-Kohlm., Mycologia 92(3): 456 (2000)

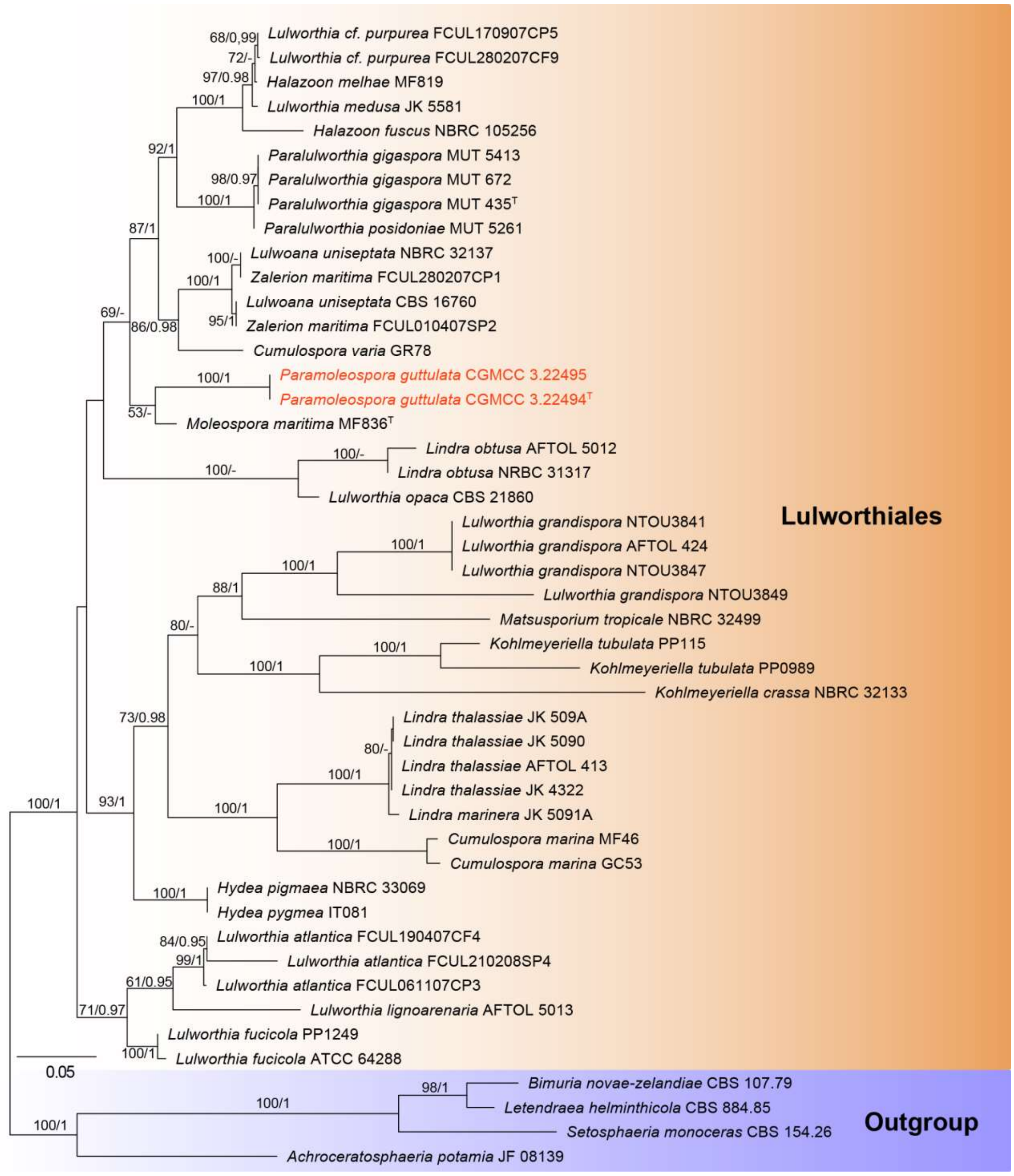

Fig. SD-33 Maximum likelihood tree of Lulworthiales based on ITS, LSU and SSU sequences. The RAxML BS above 50% and BPP above 0.90 are presented at the nodes BS/BPP. The tree was rooted to *Bimuria novae-zelandiae* CBS 107.79, *Letendraea helminthicola* CBS 884.85, *Setosphaeria monoceras* CBS 154.26 and *Achroceratosphaeria potamia* JF 08139. Ex-type cultures are indicated with a letter "T" after the accession number. The new species are printed in red font.

Paramoleospora M. Li & L. Cai, ***gen. nov.***

Fungal Names: FN571528

Etymology: Referring to the close phylogenetic relationship with the genus

Moleospora.

Asexual morph *Conidiophores* present or obsolete, when present mononematous, micronematous, septate, hyaline to light brown, cylindrical to clavate. *Conidiogenous cells* holoblastic, born on conidiophores or directly on mycelia, hyaline to brown. *Conidia* terminal or intercalary, subglobose, ellipsoidal to ovoid, thick- and smooth-walled, brown to dark brown, aseptate, guttulate. **Sexual morph** not observed.

Type: Paramoleospora guttulata M. Li & L. Cai

Notes: *Paramoleospora* is herein introduced to accommodate *P. guttulata*. It formed a distinct clade sister to *Moleospora* (Fig. SD-33), but obviously differed from the latter by the conidial shape (subglobose, ellipsoidal to ovoid *vs*. helicoid).

Paramoleospora guttulata M. Li & L. Cai, ***sp. nov.***

Fungal Names: FN571529; Fig. SD-34

Etymology: Referring to the guttulate conidia of this species.

Hyphae hyaline, septate, smooth, branched, 2.0–3.0 μm wide. **Asexual morph** *Conidiophores* present or obsolete, when present mononematous, micronematous, 2–6-septate, hyaline to light brown, cylindrical, 32–92 × 1.5–2.0 μm. *Conidiogenous cells* holoblastic, born on conidiophores or mycelia, hyaline to brown, 2.0–5.0 μm width at base. *Conidia* terminal or intercalary, sometimes forming directly from mycelia without the formation of conidiogenous cells, subglobose, ellipsoidal to ovoid, thick- and smooth-walled, brown to dark brown, aseptate, 12–21 × 7.5–14 μm (av. = 16.6 ± 1.94 × 10.2 ± 1.23 μm, n = 50), guttulate. **Sexual morph** not observed.

Culture characteristics—Colonies after 3 weeks at 25 °C, on PDA reaching 48–53 mm diam, flat, felty to pulverulent, margin irregular, whitish grey, seagreen to black, aerial mycelium moderate abundant. Reverse black near the centre, seegreen with whitish grey edge. On MEA reaching 75–77 mm diam, flat, felty, margin entire, white to black, aerial mycelium abundant. Reverse white to black. On OA reaching 38–44 mm diam, flat, felty to pulverulent, margin irregular, whitish grey to black, aerial mycelium sparse. Reverse black. Sporulation within 3 weeks on OA.

Material examined: CHINA, Guangdong, National Mangrove Nature Reserve of Zhanjiang, isolated from sediment, November 2019, M. Li and J. E. Huang, HMAS 352449 (holotype designated here, dried culture), ex-type living culture CGMCC 3.22494 = LC15932; ibid., CGMCC 3.22495 = LC15933.

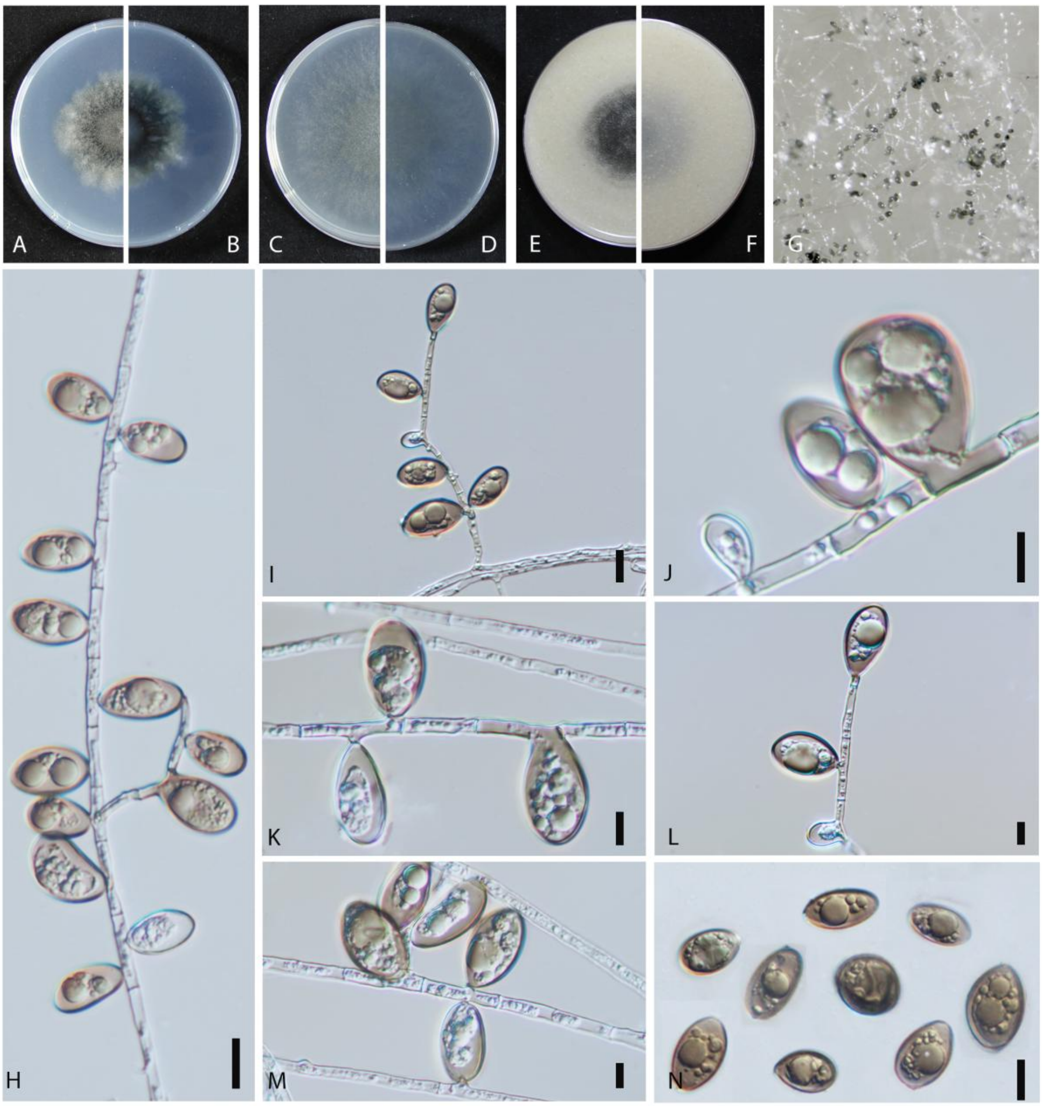

Fig. SD-35 *Paramoleospora guttulata* (from ex-holotype CGMCC 3.22494). **A–F** Surface and reverse of colony on PDA, MEA and OA. **G** Sporulation on OA. **H–M** Mycelia, conidiophores and conidiogenous cells. **N** Conidia. Scale bars: **H–I** = 20 µm. **J–N** = 5 µm.

Notes: Morphologically *P. guttulata* differs from the phylogenetically closely related species *M. maritima* in producing micronematous conidiophores, whereas species in *M. maritima* produces macronematous conidiophores. Furthermore, conidia in *P. guttulata* are terminal or intercalary, solitary and guttulate, while in *M. maritima*

conidia are lateral, eguttulate, and helicoid when young and quickly become mass. Additionally, the conidia of *P. guttulata* is longer than *M. maritima* (12–21 × 7.5–14 µm *vs*. 10–14 × 12–14 µm) (Abdel-Wahab et al. 2010).

Subclass Sordariomycetidae O.E. Erikss. & Winka, Myconet 1(1): 10 (1997)

Coniochaetales Huhndorf, A.N. Mill. & F.A. Fernández, Mycologia 96(2): 378 (2004)

Coniochaetaceae Malloch & Cain, Can. J. Bot. 49: 878 (1971)

The family *Coniochaetaceae* was established by Malloch and Cain (1971) to accommodate *Coniochaeta* and *Coniochaetidium*. The morphology of this family is characterized by the presence of germ-slits in the ascospores, which could be distinct from other family (Barr 1990, Weber 2002). Currently there are 14 genera in *Coniochaetaceae*.

Coniochaeta (Sacc.) Cooke, Grevillea 16 (no. 77): 16 (1887)

Coniochaeta was established by Hawksworth et al. (1995) to accommodate 35 species occurring mostly on dung, wood and soil. Morphologically, the genus is characterized by dark brown to black, solitary or aggregated, typically setose, pyriform to globose ascomata, with or without ostioles (Romero et al. 1999). The anamorphs of *Coniochaeta* is morphologically heterogeneous, having both enteroblastic and holoblastic ontogeny (Hawksworth 1978, Van der Linde 1991). In this study, we introduce one novel taxa to this genus from mangrove sediment (Fig. SD-35).

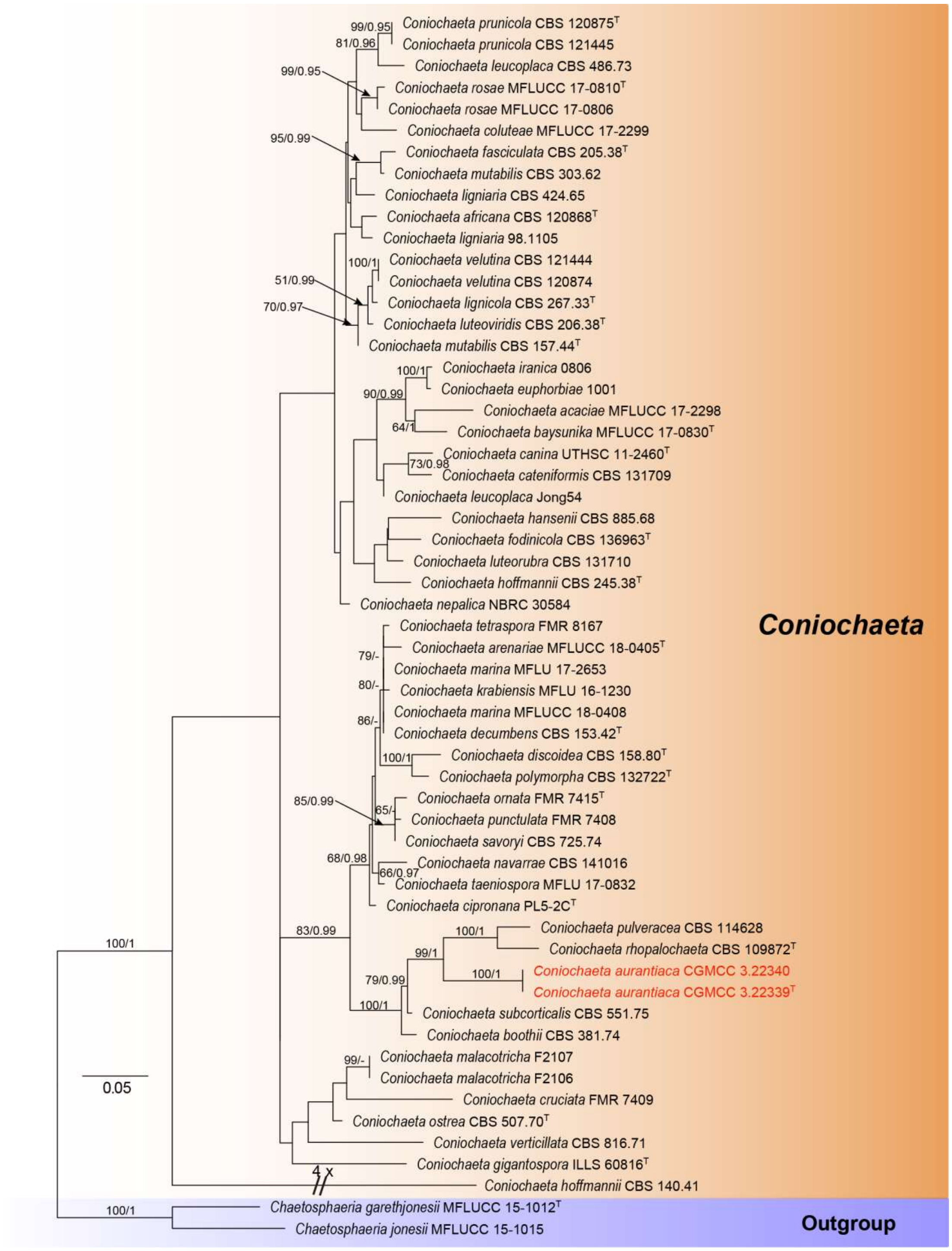

Fig. SD-35 Maximum likelihood tree of Coniochaeta based on ITS and LSU sequences. The RAxML BS above 50% and BPP above 0.90 are presented at the nodes BS/BPP. The tree was rooted to *Chaetosphaeria garethjonesii* MFLUCC 15-1012 and *Chaetosphaeria jonesii* MFLUCC 15-1015. Ex-type cultures are indicated with a letter "T" after the accession number. The new species are printed in red font

Coniochaeta aurantiaca M. Li & L. Cai, ***sp. nov.***

Fungal Names: FN571530; Fig. SD-36

Etymology: Referring to the color of its conidiomata, orange.

Hyphae hyaline, septate, smooth, branched, 2.0–3.5 μm wide. **Asexual morph** *Conidiomata* semi-immersed, subglobose, orange, normally surrounded by hyphal hairs. *Conidiophores* reduced to conidiogenous cells. *Conidiogenous cells* enteroblastic, sometimes inconspicuous, hyaline, variable in length, 4.5–17.5 × 1.5–2.5 μm, flared collarettes present or absent, when present 0.5–2.5 × 0.5–1.0 μm. *Conidia* unicellular, smooth, hyaline, mostly intercalary and terminal, few lateral, variable in size and shape, mostly subglobose, ellipsoidal to ovoid, 2.0–5.0 × 1.5–4.0 μm (av. = 3.5 ± 0.69 × 2.8 ± 0.46 μm, n = 40). **Sexual morph** not observed.

Culture characteristics—Colonies after 2 weeks at 25 °C, on PDA reaching 61–63 mm diam, flat, margin fImbriate, white, aerial mycelium sparse. Reverse white. Sporulation within 3 weeks on PDA. On MEA reaching 76–78 mm diam, flat, felty, margin entire, white, aerial mycelium extremely sparse. Reverse white.

Material examined: CHINA, Guangdong, National Mangrove Nature Reserve of Futian Shenzhen, isolated from sediment, September 2020, M. Li and Z. F. Zhang, HMAS 352432 (holotype designated here, dried culture), ex-type living culture CGMCC 3.22339 = LC15887; ibid., CGMCC 3.22340 = LC15888.

Notes: Phylogenetically, *Coniochaeta aurantiaca* is most closely related to *C. rhopalochaeta* (Fig. SD-35). Morphologically, *C. aurantiaca* can be distinguished from *C. rhopalochaeta* by conspicuous flared collarettes of conidiogenous cells, whereas *C. rhopalochaeta* produces inconspicuously flared collarettes of conidiogenous (Romero et al. 1999). Furthermore, the shape and size of conidia are different between the two species (subglobose, ellipsoidal to ovoid, 2.0–5.0 × 1.5–4.0 μm in *C. aurantiaca*; oblong-ellipsoid to cylindric, 3–5 × 5–8 μm in *C. rhopalochaeta*).

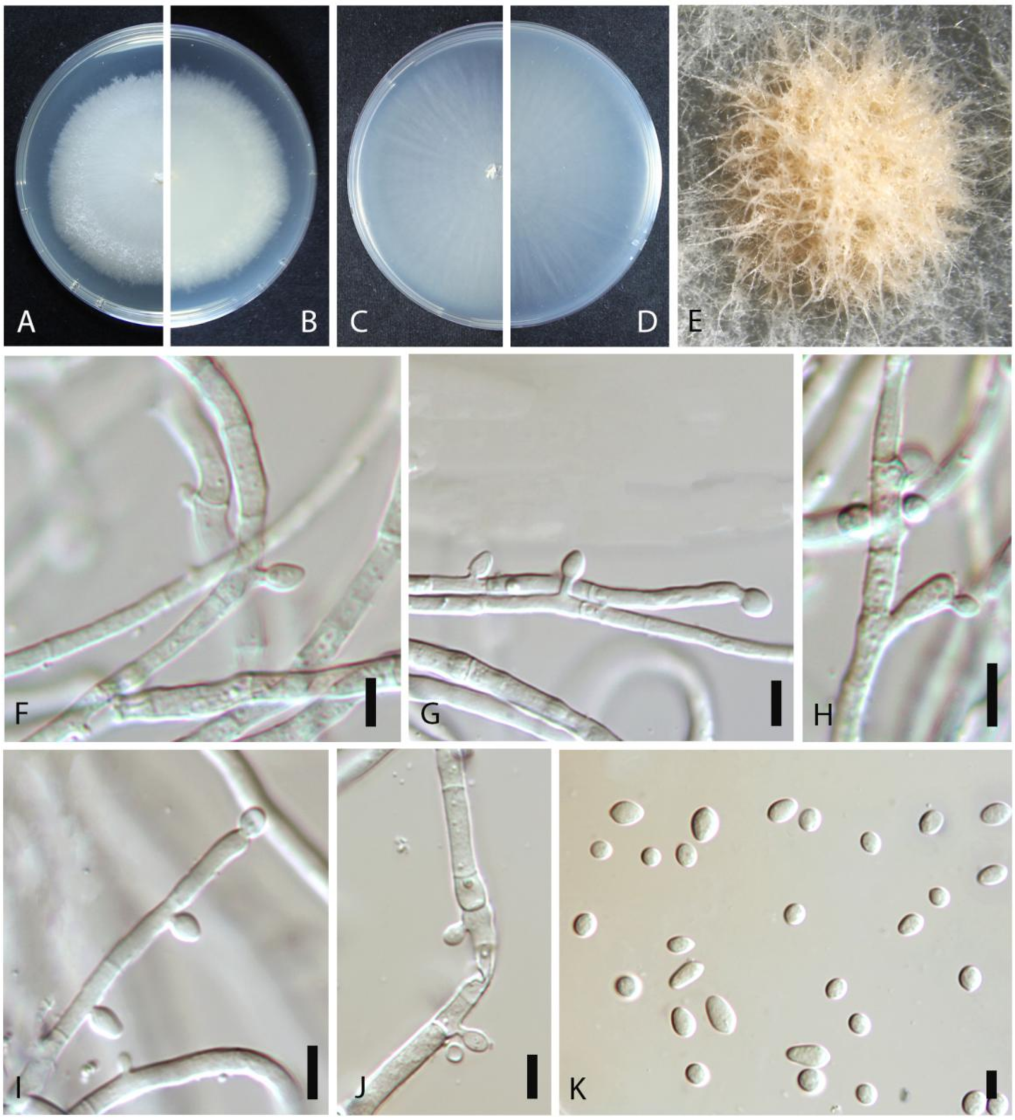

Fig. SD-36 *Coniochaeta aurantispora* (from ex-holotype CGMCC 3.22339). **A–D** Surface and reverse of colony on PDA and MEA. **E** Conidiomata on PDA. **F–J** Conidiophores and conidiogenous cells. **K** Conidia. Scale bars: **F–K** = 5 μm.

Sordariales Chadef. ex D. Hawksw. & O.E. Erikss., Syst. Ascom. 5(1): 182 (1986)

Schizotheciaceae Y. Marin & Stchigel, Microorganisms 8(9, no. 1430): 24 (2020)

Schizotheciaceae was established by Marin-Felix et al. (2020) to accommodate lasiosphaeriaceous taxa characterized by producing mostly ornate, ostiolate ascomata with different types of structures, and one- or two-celled ascospores (Marin-Felix et al. 2020). Currently, ten genera are included in *Schizotheciaceae* (Harms et al. 2021). In this study, we introduce one new genus *Neomorinagamyces* to accommodate *N.*

pyriformis sp. nov. (Fig. SD-37).

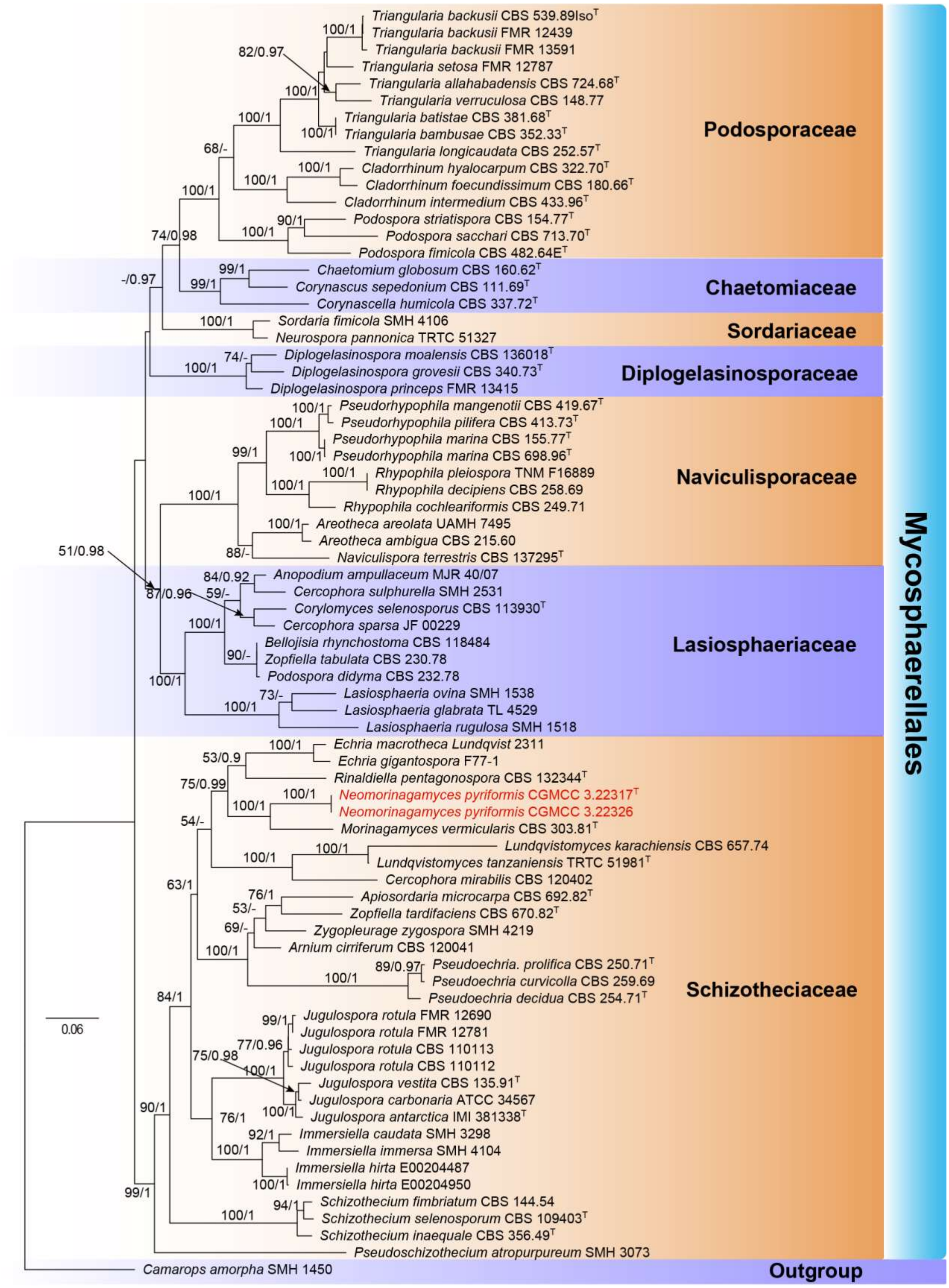

Fig. SD-37 Maximum likelihood tree of Mycosphaerellales based on ITS, LSU, *tub2* and *rpb2* sequences. The RAxML BS above 50% and BPP above 0.90 are presented at the nodes BS/BPP. The tree was rooted to *Camarops amorpha* SMH 1450. Ex-type cultures are indicated with a letter "T" after the accession number. The new species are printed in red font.

Neomorinagamyces M. Li & L. Cai, ***gen. nov.***

Fungal Names: FN571530

Etymology: Referring to the close phylogenetic relationship with the genus *Morinagamyces*.

Asexual morph *Conidiophores* mononematous, macronematous, subcylindrical, smooth, straight or flexuous, septate, branched or unbranched, light brown, sometimes reduced to conidiogenous cells. *Conidiogenous cells* entroblastic, phialidic, light brown, smooth, ampulliform to subcylindrical, slightly constricted at the base and gradually tapering toward the apex, with a terminal collarette. *Collarette* funnel-shaped, light brown. *Conidia* forming small slimy heads on the tips of the phialides, pyriform, guttulate, thin- and smooth-walled, hyaline, aseptate. **Sexual morph** not observed.

Type: Neomorinagamyces pyriformis M. Li & L. Cai

Notes: *Neomorinagamyces* is herein introduced to accommodate *N. pyriformis*. Two strains representing *N. pyriformis* clustered together in an independent clade sister to *Morinagamyces* (Fig. SD-37). Morphologically, two genera are different in the type of conidiophores and conidiogenous cells (macronematous conidiophores and entroblastic conidiogenous cells in *Neomorinagamyces;* micronematous conidiophores and holoblastic conidiogenous cells in *Morinagamyces*).

Neomorinagamyces pyriformis M. Li & L. Cai, sp. ***nov.***

Fungal Names: FN571532; Fig. SD-38

Etymology: Referring to the pyriform conidia of this species.

Hyphae light brown, septate, smooth, branched, 1.5–2.0 µm wide. **Asexual morph** *Conidiophores* mononematous, macronematous, subcylindrical, smooth, straight or flexuous, sepate, branched or unbranched, light brown, 13–68 × 1.0–1.5 µm, sometimes reduced to conidiogenous cells. *Conidiogenous cells* entroblastic, phialidic, light brown, smooth, ampulliform or dolioform, slightly constricted at the base and gradually tapering toward the apex, with a terminal collarette, 10–21 × 3.0–4.0 µm. *Collarette* funnel-shaped, 1.5–2.0 µm wide at the opening, 0.5–1.0 µm

wide at the base, light brown. *Conidia* forming small heads on the tips of the phialides, pyriform, guttulate, thin- and smooth-walled, hyaline, aseptate, 2.0–2.5 × 1.5–2.0 µm (av. = 1.1 ± 0.15 × 1.9 ± 0.11 µm, n = 50). *Chlamydospores* usually present, intercalary, smooth, thick-walled, dark brown, septate, 4.5–6.5 × 4.5–5.5 µm. **Sexual morph** not observed.

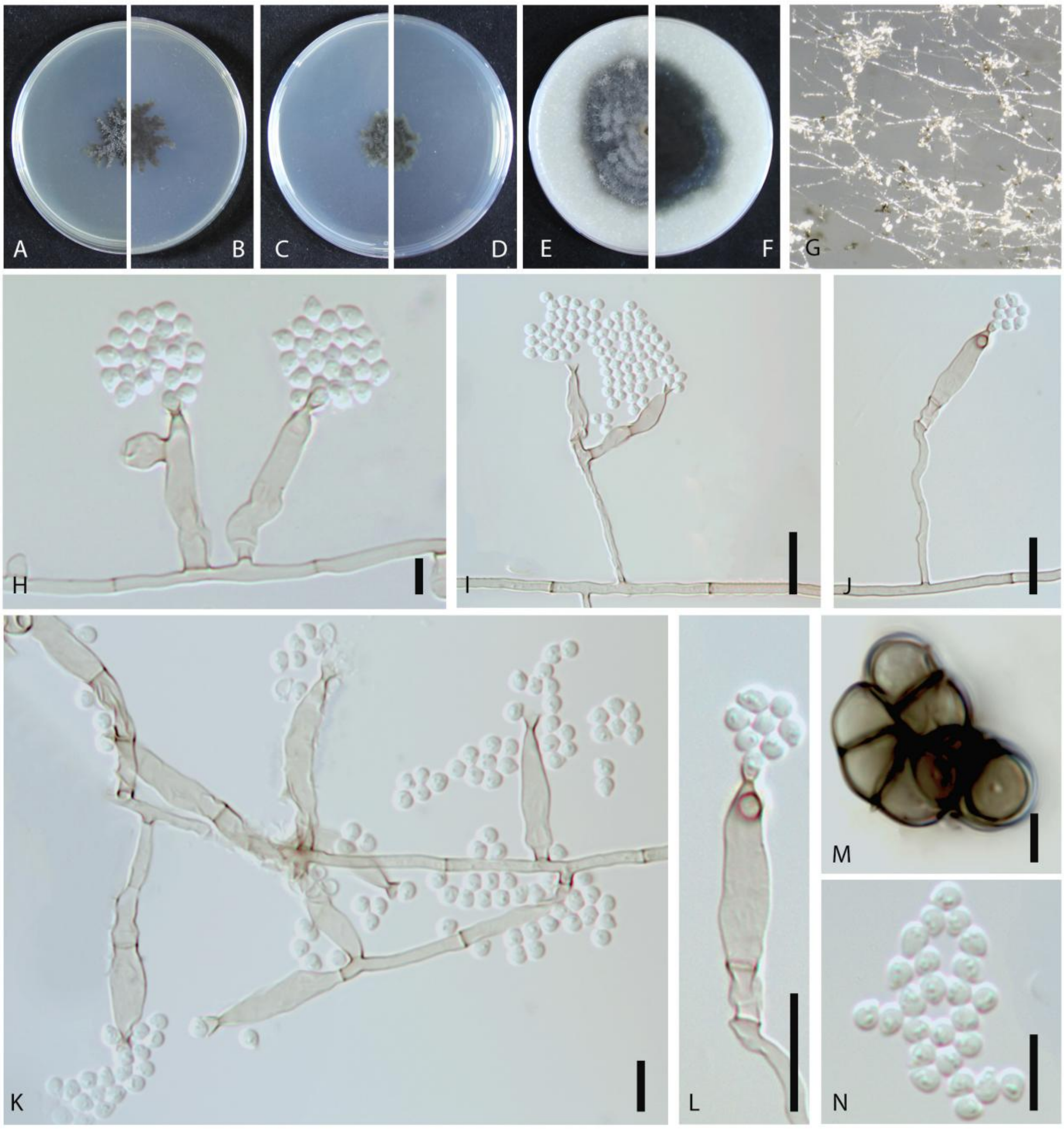

Fig. SD-38 *Neomorinagamyces doliiformis* (from ex-holotype CGMCC 3.22317). **A–F** Surface and reverse of colony on PDA, MEA and OA. **G** Sporulation on WA. **H–L** Conidiophores, conidiogenous cells and conidia. **M** Chlamydospores. **N** Conidia. Scale bars: **H–N** = 5 µm.

Culture characteristics—Colonies after 4 weeks at 25 °C, on PDA reaching 30–35 mm diam, flat, felty, margin irregular, white, olive to black, aerial mycelium

sparse. Reverse olive to black. On MEA reaching 24–27 mm diam, flat, felty, margin irregular, olive to black, aerial mycelium sparse. Reverse olive to black. On OA reaching 55–59 mm diam, flat, floccose, annular, margin irregular, whitish grey to black, aerial mycelium sparse. Reverse black.

Material examined: CHINA, Guangdong, National Mangrove Nature Reserve of Futian Shenzhen, isolated from sediment, September 2020, M. Li and Z. F. Zhang, HMAS 352430 (holotype designated here, dried culture), ex-type living culture CGMCC 3.22317 = LC15883; ibid., CGMCC 3.22326 = LC15884.

Notes: *Neomorinagamyces pyriformis* morphologically differs from its phylogenetically closely related species *M. vermicularis* in producing macronematous conidiophores, whereas the latter species produces micronematous conidiophores. Furthermore, conidiogenous cells in *N. pyriformis* are entroblastic, while in *M. vermicularis* conidiogenous cells are entroblastic or holoblastic. Additionally, *N. pyriformis* produces guttulate conidia, which were not observed in *M. vermicularis* (Harms et al. 2021).

References

Abdel-Wahab MA, Pang KL, Nagahama T, et al. (2010) Phylogenetic evaluation of anamorphic species of *Cirrenalia* and *Cumulospora* with the description of eight new genera and four new species. Mycol Prog 9: 537–558.

Ariyawansa HA, Hyde KD, Jayasiri SC, et al. (2015) Fungal diversity notes 111–252—taxonomic and phylogenetic contributions to fungal taxa. Fungal Divers 75: 27–274.

Barr ME (1990) Melanommatales (Loculoascomycetes). North American Flora, Series II, Part 13: 1–129.

Biourge P (1923) Les moissisures du groupe *Penicillium* Link. La Cellule 33: 7–331.

Boonmee S, Wanasinghe DN, Calabon MS, et al. (2021) Fungal diversity notes 1387–1511: taxonomic and phylogenetic contributions on genera and species of fungal taxa. Fungal Divers 111: 1–335.

Crous PW, Carris LM, Giraldo A, et al. (2015) The genera of fungi—fixing the application of the type species of generic names—G2: *Allantophomopsis*, *Latorua*, *Macrodiplodiopsis*, *Macrohilum*, *Milospium*, *Protostegia*, *Pyricularia*, *Robillarda*, *Rotula*, *Septoriella, Torula* and *Wojnowicia*. IMA Fungus 6:163–198.

Crous PW, Wingfield MJ. Guarro J, et al. (2013) Fungal Planet description sheets: 154–213. Persoonia 31: 188–296.

Crous PW, Wingfield MJ, Schumacher RK, et al. (2014) Fungal Planet description sheets: 281–319. Persoonia 33: 212–289.

da Silva Santos AC, Diniz AG, Tiago PV, et al. (2020) Entomopathogenic *Fusarium* species: a review of their potential for the biological control of insects, implications and prospects. Fungal Biol Rev 34: 41–57.

Delgado G, Miller AN, Piepenbring M (2018) South Florida microfungi: *Castanedospora*, a new genus to accommodate *Sporidesmium pachyanthicola* (Capnodiales, Ascomycota). Cryptogamie, Mycologie 39: 109–127.

Doveri F, Pecchia S, Vergara M, et al. (2012) A comparative study of *Neogymnomyces virgineus*, a new keratinolytic species from dung, and its relationships with the Onygenales. Fungal Divers 52:13–34.

Gams W (1971) *Cephalosporium-artige Schimmelpilz*e (Hyphomycetes). Gustav Fischer Verlag, Stuttgart, Germany.

Gams W, Stielow B, Gräfenhan T, et al. (2019) The ascomycete genus *Niesslia* and associated monocillium-like anamorphs. Mycol Prog 18: 5–76.

Gueidan C, Aptroot A, da Silva Cáceres ME, et al. (2014) A reappraisal of orders and families within the subclass Chaetothyriomycetidae (Eurotiomycetes, Ascomycota). Mycol Prog 13: 990.

Harms K, Surup F, Stadler M, et al. (2021) Morinagadepsin, a Depsipeptide from the Fungus *Morinagamyces vermicularis* gen. et comb. nov. Microorganisms 9: 1191.

Hawksworth DL (1978) A new species of *Coniochaeta* with an interesting conidial state. Norw J Bot 25: 15–18.

Houbraken J, de Vries RP, Samson RA (2014) Modern taxonomy of biotechnologically important *Aspergillus* and *Penicillium* species. Adv Appl Microbiol 86: 199–249.

Houbraken J, Kocsubé S, Visagie C, et al. (2020) Classification of *Aspergillus*, *Penicillium*,

Talaromyces and related genera (Eurotiales): An overview of families, genera, subgenera, sections, series and species. Stud Mycol 96: 141–153.

Houbraken J, Samson RA (2011) Phylogeny of *Penicillium* and the segregation Trichocomaceae into three families. Stud Mycol 70: 1–51.

Howard DH, Weitzman I, Padhye AA. (2003) Onygenales: Arthrodermataceae. In: Howard DH, editor. Pathogenic Fungi in Humans and Animals. NewYork, NY: Marcel Dekker Inc; p. 141–195.

Huang L, Qi, ZT (1994) A new *Penicillium* from *Phaseolus angularis*. Acta Mycologica Sinica 13: 264–266.

Hubka V, Dobiasova S, Lyskova P, et al. (2013) *Auxarthron ostraviense* sp. nov., and *A. umbrinum* associated with non-dermatophytic onychomycosis. Medical mycology 51: 614–624.

Hyde KD, Jeewon R, Chen YJ, et al. (2020) The numbers of fungi: is the descriptive curve flattening?. Fungal Divers 103: 219–271.

Jaklitsch WM, Voglmayer H (2016) Hidden diversity in *Thyridaria* and a new circumscription of the Thyridariaceae. Stud Mycol 85:35–64.

Jayasiri SC, Hyde KD, Jones EBG, et al. (2019) Diversity, morphology and molecular phylogeny of Dothideomycetes on decaying wild seed pods and fruits. Mycosphere 10: 1–186.

Jiang HB, Hyde KD, Jayawardene RS, et al. (2019) Taxonomic and phylogenetic characterizations reveal two new species and two new records of *Roussoella* (Roussoellaceae, Pleosporales) from Yunnan, China. Mycol Prog 18: 577–591.

Karunarathna A, Phookamsak R, Jayawardena RS, et al. (2019) The holomorph of *Neoroussoella* alishanense sp. nov. (Roussoellaceae, Pleosporales) on *Pennisetum purpureum* (Poaceae). Phytotaxa 406: 218–236.

Kong HZ, Qi ZT (1988) Three new species of *Penicillium*. Mycosystema 1: 107–114.

La Touche CJ (1968). *Apinisia graminicola* gen. et sp. nov. Transactions of the British. Trans Br mycol Soc 51: 283–285.

Leslie JF, Summerell BA (2006) The *Fusarium* Laboratory Manual. Blackwell Publishing: 1–388.

Link HF (1809) Observationes in ordines plantarum naturals, Dissetatio I. Mag Ges Naturf Freunde Berlin 3: 3–42.

Link JHF (1826) Entwurf eines phytologischen Pfanzensystems nebst einer Anordnung der Kryptophyten. Abh dt Akad Wiss Berlin 1824: 145–194.

Liu JK, Phookamsak R, Dai DQ, et al. (2014) Roussoellaceae, a new pleosporalean family to accommodate the genera *Neoroussoella* gen. nov., *Roussoella* and *Roussoellopsis*. Phytotaxa 181: 1–33.

Lombard L, Van der Merwe NA, Groenewald JZ, et al. (2015) Generic concepts in Nectriaceae. Stud Mycol 80: 189–245.

Lombard L, Van Doorn R, Crous PW (2019) Neotypification of *Fusarium chlamydosporum* reappraisal of a clinically important species complex. Fungal Syst Evol: 183–200.

Lumbsch HT, Huhndorf SM (2010) Myconet volume 14 part one. Outline of Ascomycota–2009. Fieldiana Life Earth Sci 1: 1–922.

Maharachchikumbura SSN, Hyde KD, Jones EBG, et al. (2015) Towards a natural classification and backbone tree for Sordariomycetes. Fungal Divers 72: 199–301.

Maharachchikumbura SSN, Hyde KD, Jones EBG, et al. (2016) Families of Sordariomycetes. Fungal Divers 79: 1–317.

Malloch D, Cain RF, (1971) New cleisthothecial Sordariaceae and a new family Coniochaetaceae. Can J Bot 49: 869–880.

Mapook A, Hyde KD, McKenzie EHC (2020) Taxonomic and phylogenetic contributions to fungi associated with the invasive weed *Chromolaena odorata* (Siam weed). Fungal Divers 101: 1–175.

Marin-Felix Y, Miller AN, Cano-Lira JF, et al. (2020) Re-evaluation of the order Sordariales: Delimitation of Lasiosphaeriaceae s. str., and introduction of the new families Diplogelasinosporaceae, Naviculisporaceae, and Schizotheciaceae. Microorganisms 8: 1430.

Miller JH. (1949) A revision of the classification of the Ascomycetes with special emphasis on the Pyrenomycetes. Mycologia 41: 99–127.

Orr GF, Kuehn HH, Plunkett OA (1963) A new genus of the Gymnoascaceae with swollen peridial septa. Can J of Bot 41: 1439–1456.

Panasenko VT (1964) Some new species of fungi on starch from the ukraine. Mycologia 56: 58–63.

Pang KL, Alias SA, Chiang MWL, et al. (2010) *Sedecimiella taiwanensis* gen. et sp. nov., a marine mangrove fungus in the Hypocreales (Hypocreomycetidae, Ascomycota). Bot Mar 53: 493–498.

Pang KL, Jones EBG, Vrijmoed LLP, et al. (2004) *Okeanomyces*, a new genus to accommodate *Halosphaeria cucullata* (Halosphaeriales, Ascomycota). Bot J Linn Soc 146: 223–229.

Phookamsak R, Hyde KD, Jeewon R, et al. (2019) Fungal diversity notes 929–1036: taxonomic and phylogenetic contributions on genera and species of fungal taxa. Fungal Divers 95: 1–273.

Phukhamsakda C, McKenzie EH, Phillips AJ, et al. (2020) Microfungi associated with *Clematis* (Ranunculaceae) with an integrated approach to delimiting species boundaries. Fungal Divers 102: 1–203.

Punithalingam E (1979) Sphaeropsidales in culture from humans. Nova Hedwigia 31: 119–158.

Quaedvlieg W, Binder M, Groenewald JZ, et al. (2014) Introducing the consolidated species concept to resolve species in the Teratosphaeriaceae. Persoonia 33: 1–40.

Rogerson CT (1970) The hypocrealean fungi (Ascomycetes, Hypocreales). Mycologia 62: 865–910.

Romero AI, Carmarán CC, Lorenzo LE (1999) A new species of *Coniochaeta* with a key to the species known in Argentina. Mycol Res 103: 689–695.

Romero SM, Romero AI, Barrera V, et al. (2016) *Talaromyces systylus*, a new synnematous species from argentinean semi-arid soil. Nova Hedwigia 102: 241–256.

Rossman AY, Samuels GJ, Rogerson CT, et al. (1999) Genera of Bionectriaceae, Hypocreaceae, and Nectriaceae (Hypocreales, Ascomycetes). Stud Mycol 42: 1–248.

Samson RA, Polonelli L (1978) *Myriodontium keratinophilum*, gen. et sp. nov. Persoonia 9: 505–509.

Sarrocco S, Diquattro S, Baroncelli R, et al. (2015) A polyphasic contribution to the knowledge of *Auxarthron* (Onygenaceae). Mycol prog 14: 1–20.

Sigler L, Hambleton S, Flis AL, et al. (2002) *Auxarthron* teleomorphs for *Malbranchea flamentosa* and *Malbranchea albolutea* and relationships within *Auxarthron*. Stud Mycol 47: 111–122.

Smith, G (1957) Some new and interesting species of micro-fungi. Trans Br Mycol Soc 40:

481–IN6.

Sugiyama M, Ohara A, Mikawa T (1999) Molecular phylogeny of onygenalean fungi based on small subunit ribosomal DNA (SSU rDNA) sequences. Mycoscience 40: 251–258.

Tanaka K, Hirayama K, Yonezawa H, et al. (2015) Revision of the *Massarineae* (Pleosporales, Dothideomycetes). Stud Mycol 82: 75–136.

Tibpromma S, Hyde KD, McKenzie EHC, et al. (2018) Fungal diversity notes 840–928: micro-fungi associated with Pandanaceae. Fungal Divers 93: 1–160.

Tulasne LR, Tulasne C (1865) Selecta fungorum carpologia III. Paris Museum, pp 1–221.

Ueda, S (1995) A new species of *Euoenicillium* from marine sediment. Mycoscience 4: 451–454.

Van der Linde EJ (1991) *Coniochaeta cypraespora* sp. nov. with a *Paecilomyces* conidial state. Mycol Res 95: 510–512.

von Arx JA (1954) Gattungen der amerosporen Pyrenomyceten. Beitr Kryptogamenflora Schweiz 11: 1–434.

Wanasinghe DN, Phukhamsakda C, Hyde KD, et al. (2018) Fungal diversity notes 709–839: taxonomic and phylogenetic contributions to fungal taxa withan emphasis on fungi on Rosaceae. Fungal Divers 89: 1–236.

Weber E (2002) The *Lecythophora–Coniochaeta* complex I. Morphological studies on *Lecythophora* species isolated from *Picea abies*. Nova Hedwigia 74: 159–185.

Wijayawardene NN, Hyde KD, Lumbsch HT, et al. (2018) Outline of Ascomycota: 2017. Fungal Divers 88: 167–263.

Zhang ZF, Zhou SY, Eurwilaichitr L, et al. (2021) Culturable mycobiota from Karst caves in China II, with descriptions of 33 new species. Fungal Divers106: 29–136.

Zhang ZK, Wang XC, Zhuang WY, et al. (2021) New species of *Talaromyces* (Fungi) isolated from soil in Southwestern China. Biology 10: 745.
