# Supplementary material for: Application of culturomics in fungal isolation from mangrove sediments
Source: Microbiome. 2023 Dec 11;11:272. doi: 10.1186/s40168-023-01708-6 (PMC10712113; doi:10.1186/s40168-023-01708-6)
Supplement: Supplementary file 4 — Additional file 3. The extended experimental procedures of fungal enrichment culture method (FECM) and in situ cultivation using fungal isolation chips (FiChips) and supplementary files. Supplementary Fig. 1. The heatmap for the diversity of species from three sediment samples during each stage of enrichment incubation (color ranging represents strains number). D0, D7, D14, and D21 indicate the isolation results of FECM on days 0, 7, 14 and 21, respectively. Source data are provided as a Source data file. Supplementary Fig. 2. The heatmap for the diversity of species from sediment samples SZ-1 and SZ-2 (color ranging represents relative abundance (%)). HTS, DPM, and FiChip indicate the results of high through-put sequencing, dilution-plate method, and in situ cultivation, respectively. Source data are provided as a Source data file. Supplementary Fig. 3. Phylogenetic tree of species isolated from 66 sediment samples. The phylogenetic tree was constructed from the full-length ITS gene sequences. Major phylum names are indicated and marked in different colors. Red branches on the trees indicate that the species was isolated only from new fungal culturomics techniques (FECM and FiChip). Candidate novel species are indicated by stars (blue stars indicate ITS sequence similarity between 94% and 98% comparing to the NCBI database, green stars indicate similarity between 91% and 94%, and red stars indicate similarity below 91%). In addition, detailed information of each species is shown in Additional file2: Table S5. Supplementary Fig. 4. Venn diagram between new fungal culturomics techniques (NFCT) and large-scale fungal isolation (LSFI). (a). Comparison of species diversity between NFCT and LSFI. (b). Comparison of the number of potential novel species between NFCT and LSFI. Supplementary Fig. 5. Comparison of node-level topological features in Fig. 5a (degree and closeness centrality) demonstrating the high degree and closeness centrality for the unidentified and other [file 40168_2023_1708_MOESM3_ESM.pdf]

Additional file 3

Supplementary information

Application of culturomics in fungal isolation from mangrove sediments

Meng Li[1,2†], Mubashar Raza[1,3†], Shuang Song[1,2], Lingwei Hou[4], Zhi-Feng Zhang[5], Min Gao[1,2], Jun-En Huang[1,2], Fang Liu[1,2*], Lei Cai[1,2*]

[*]Authors for correspondence: Lei Cai (email: cail@im.ac.cn); Fang Liu (email: liufang@im.ac.cn)

Extended Experimental Procedures

Fungal enrichment culture method (FECM)

Enrichment cultivation was performed at 25 °C for 21 days in 500 mL sealed glass shake flasks (filled with 400 mL Potato Dextrose Broth medium and 20 g of sediment sample). The bottles were shaken in Blue Pard THZ-98C shaker under 150 rpm/min, 25 °C and kept sealed during the cultivation. Three replicates were set for each sample. On the 0th, 7th, 14th and 21st days, 11 mL of culture broth was taken from the shake flask, 10 mL of which was kept in the sterile tubes and stored at -80°C for DNA extraction, and the other 1 mL was transferred to a sterile test tubes and diluted with 9 mL sterile seawater and spread onto plates with five different culture media (PDASS, MEASS, MMSS, CMASS, and CDASS) The plates were then incubated at 25 °C for 7–30 days, and from which the single colonies were picked up and inoculated onto new PDASS plates. All fungal strains were stored at 4 °C in sterile water with 3.5% sea salt during the course of this study.

In situ cultivation using fungal isolation chips (FiChips)

1. FiChips assembly after inoculation with fungal dilution from sediment samples under a laminar flow hood

(1) Apply two thin layers of silicone glue to one side of a customized 96 through-holes polypropylene isolation plate. This step should be performed in a laminar flow hood or fume cupboard.

(2) Place a piece of precut bylon membrane flat on the surface of the 96-well plate. Hold the FiChip at an angle, gently place one side of the FiChip at the edge of the membrane, leaving about 5 mm exposed on the edge. Gently place the FiChip on

the membrane and apply light pressure.

(3) Let the glue dry for at least 12 h.

(4) Cut off the excess nylon membrane exposed outside the FiChip, then wrap the FiChip individually with white paper and autoclave at 121 °C for 30 min.

(5) Dry the FiChips then put them in a laminar flow cabinet for further use.

(6) Pour the seawater agar into a sterile pipette basin. Use a multichannel pipette to save more time that prevent agar from solidifying. Dispense 100 μL of the seawater agar into each well of the FiChip.

(7) Let stand for 30 minutes to solidify the medium.

(8) Prepare sediment suspension. Dilute the sediment 50 times with sterilized seawater (collected near the mangrove sediment sampling site).

(9) Dispense 10 μL of the sediment suspension into each well of the FiChip by using a multichannel pipette.

(10) Apply a thin layer of silicone glue to the top of the FiChip. Adhere a precut piece of sterile membrane to the top of the FiChip as in Steps 1 and 2.

(11) Let the glue dry for 3 h or until the smell of glue is no longer detectable.

2. *In situ* incubation in the mangrove sediments by FiChips placed into medical stainless steel mesh frame

A stainless steel mesh frame with 15 FiChips was placed at each sampling site for one-month incubation.

3. FiChips dug out of mangrove sediments after one-month incubation

Collect the FiChip from sediment and rinse well with sterile water 4-5 times to ensure that all of the sediment is removed from the device

4. Isolation and purification under laboratory conditions

(1) Use a sterile tweezer to gently peel off the top membrane in the laminar flow cabinet.

(2) Examine and pick up each microcolony onto new PDASS plates using a sterile toothpick or other sterile sharp object under a Nikon SMZ1500 dissecting microscope.

(3) Incubate the PDASS plates at the same temperature as that of the sediment in its natural environment.

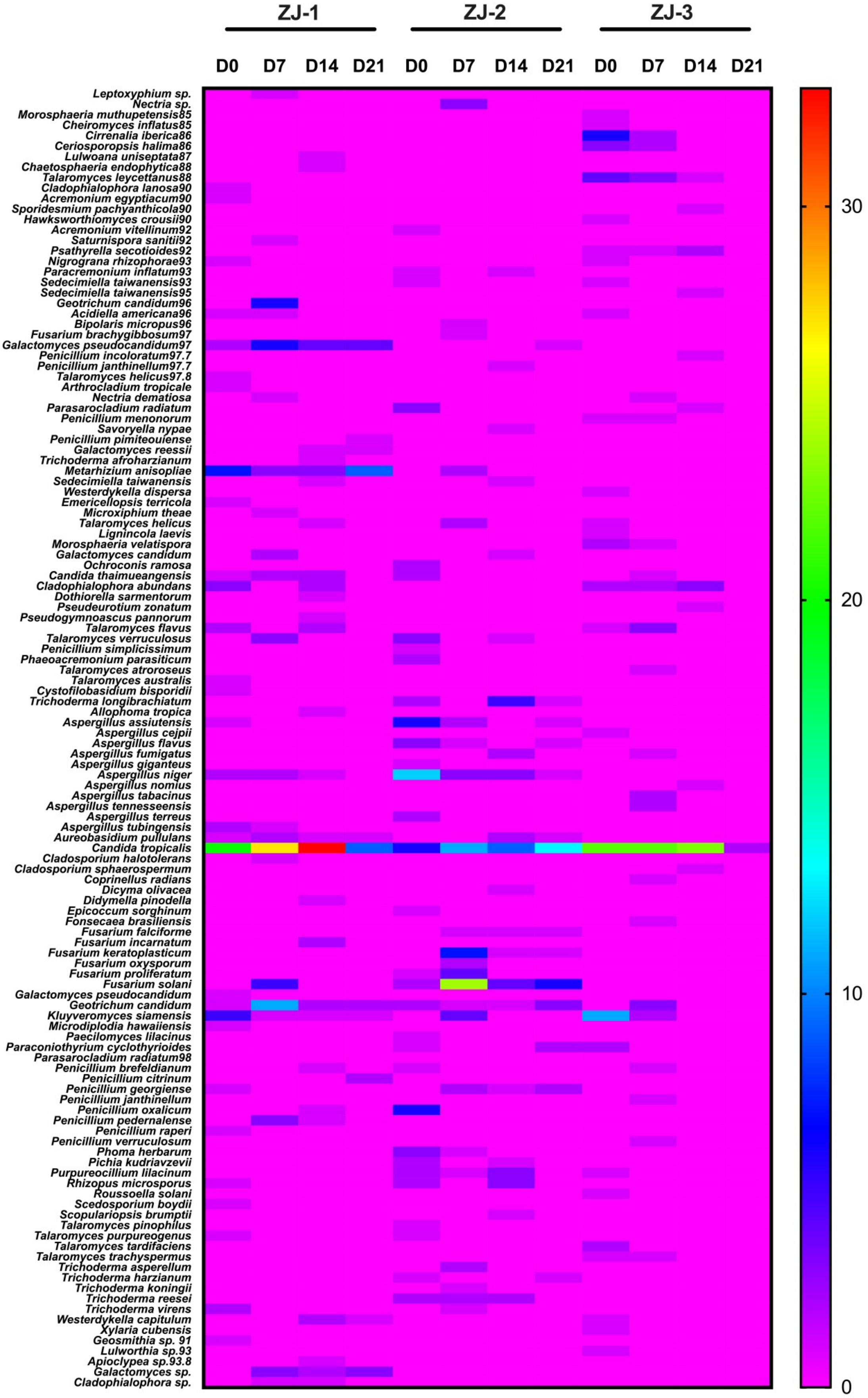

ZJ-1
ZJ-2
ZJ-3
D0
D7
D14
D21
D0
D7
D14
D21
D0
D7
D14
D21
Leptoxyphium sp.
Nectria sp.
Morosphaeria muthupetensis85
Cheiromyces inflatus85
Cirrenalia iberica86
Ceriosporopsis halima86
Lulwoana uniseptata87
Chaetosphaeria endophytica88
Talaromyces leycettanus88
Cladophialophora lanosa90
Acremonium egyptiacum90
Sporidesmium pachyanthicola90
Hawksworthiomyces crousii90
Acremonium vitellinum92
Saturnispora sanitii92
Psathyrella secotioides92
Nigrograna rhizophorae93
Paracremonium inflatum93
Sedecimiella taiwanensis93
Sedecimiella taiwanensis95
Geotrichum candidum96
Acidiella americana96
Bipolaris micropus96
Fusarium brachygibbosum97
Galactomyces pseudocandidum97
Penicillium incoloratum97.7
Penicillium janthinellum97.7
Talaromyces helicus97.8
Arthrocladium tropicale
Nectria dematiosa
Parasarocladium radiatum
Penicillium menonorum
Savoryella nypae
Penicillium pimiteouiense
Galactomyces reessii
Trichoderma afroharzianum
Metarhizium anisopliae
Sedecimiella taiwanensis
Westerdykella dispersa
Emericellopsis terricola
Microxiphium theae
Talaromyces helicus
Lignincola laevis
Morosphaeria velatispora
Galactomyces candidum
Ochroconis ramosa
Candida thaimueangensis
Cladophialophora abundans
Dothiorella sarmentorum
Pseudeurotium zonatum
Pseudogymnoascus pannorum
Talaromyces flavus
Talaromyces verruculosus
Penicillium simplicissimum
Phaeoacremonium parasiticum
Talaromyces atroroseus
Talaromyces australis
Cystofilobasidium bisporidii
Trichoderma longibrachiatum
Allophoma tropica
Aspergillus assiutensis
Aspergillus cejpii
Aspergillus flavus
Aspergillus fumigatus
Aspergillus giganteus
Aspergillus niger
Aspergillus nomius
Aspergillus tabacinus
Aspergillus tennesseensis
Aspergillus terreus
Aspergillus tubingensis
Aureobasidium pullulans
Candida tropicalis
Cladosporium halotolerans
Cladosporium sphaerospermum
Coprinellus radians
Dicyma olivacea
Didymella pinodella
Epicoccum sorghinum
Fonsecaea brasiliensis
Fusarium falciforme
Fusarium incarnatum
Fusarium keratoplasticum
Fusarium oxysporum
Fusarium proliferatum
Fusarium solani
Galactomyces pseudocandidum
Geotrichum candidum
Kluyveromyces siamensis
Microdiplodia hawaiiensis
Paecilomyces lilacinus
Paraconiothyrium cyclothyrioides
Parasarocladium radiatum98
Penicillium brefeldianum
Penicillium citrinum
Penicillium georgiense
Penicillium janthinellum
Penicillium oxalicum
Penicillium pedernalense
Penicillium raperi
Penicillium verruculosum
Phoma herbarum
Pichia kudriavzevii
Purpureocillium lilacinum
Rhizopus microsporus
Roussoella solani
Scedosporium boydii
Scopulariopsis brumptii
Talaromyces pinophilus
Talaromyces purpureogenus
Talaromyces tardifaciens
Talaromyces trachyspermus
Trichoderma asperellum
Trichoderma harzianum
Trichoderma koningii
Trichoderma reesei
Trichoderma virens
Westerdykella capitulum
Xylaria cubensis
Geosmithia sp. 91
Lulworthia sp.93
Apioclypea sp.93.8
Galactomyces sp.
Cladophialophora sp.
30
20
10
0

Supplementary Fig. 1. The heatmap for the diversity of species from three sediment samples during each stage of enrichment incubation (color ranging represents strains number). D0, D7, D14, and D21 indicate the isolation results of FECM on days 0, 7, 14 and 21, respectively. Source data are provided as a Source data file.

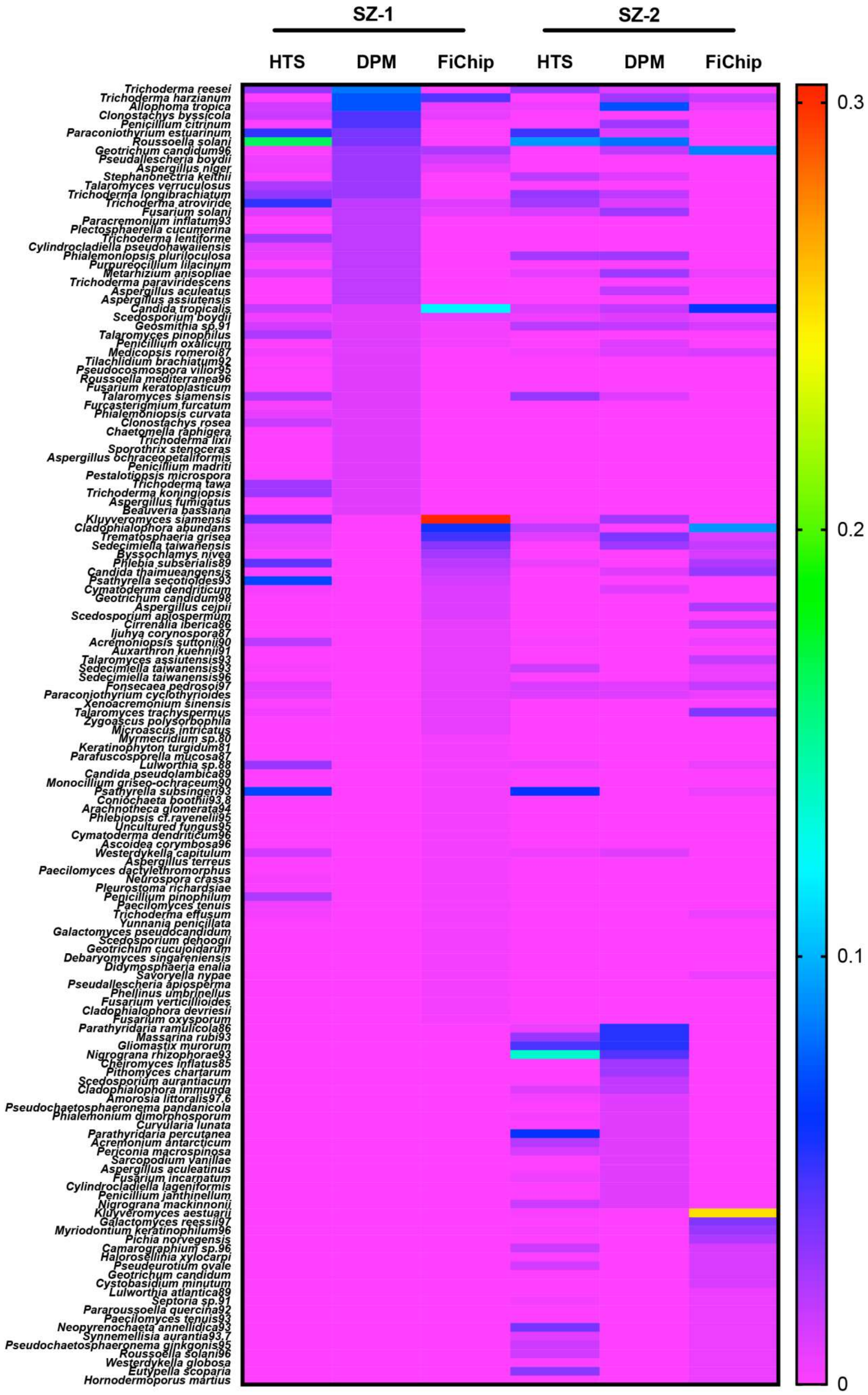
SZ-1
SZ-2
HTS
DPM
FiChip
HTS
DPM
FiChip
Trichoderma reesei
Trichoderma harzianum
Allophoma tropica
Clonostachys byssicola
Penicillium citrinum
Paraconiothyrium estuarinum
Roussoella solani
Geotrichum candidum96
Pseudallescheria boydii
Aspergillus niger
Stephanonectria keithii
Talaromyces verruculosus
Trichoderma longibrachiatum
Trichoderma atroviride
Fusarium solani
Paracremonium inflatum93
Plectosphaerella cucumerina
Trichoderma lentiforme
Cylindrocladiella pseudohawaiiensis
Phialemoniopsis pluriloculosa
Purpureocillium lilacinum
Metarhizium anisopliae
Trichoderma paraviridescens
Aspergillus aculeatus
Aspergillus assiutensis
Candida tropicalis
Scedosporium boydii
Geosmithia sp.91
Talaromyces pinophilus
Penicillium oxalicum
Medicopsis romeroi87
Tilachlidium brachiatum92
Pseudocosmospora vilior95
Roussoella mediterranea96
Fusarium keratoplasticum
Talaromyces siamensis
Furcasterigmium furcatum
Phialemoniopsis curvata
Clonostachys rosea
Chaetomella raphigera
Trichoderma lixii
Sporothrix stenoceras
Aspergillus ochraceopetaliformis
Penicillium madriti
Pestalotiopsis microspora
Trichoderma tawa
Trichoderma koningiopsis
Aspergillus fumigatus
Beauveria bassiana
Kluyveromyces siamensis
Cladophialophora abundans
Trematosphaeria grisea
Sedecimiella taiwanensis
Byssochlamys nivea
Phlebia subserialis89
Candida thaimueangensis
Psathyrella secotioides93
Cymatoderma dendriticum
Geotrichum candidum98
Aspergillus cejpii
Scedosporium apiospermum
Cirrenalia iberica86
Ijuhya corynospora87
Acremoniopsis suttonii90
Auxarthron kuehnii91
Talaromyces assiutensis93
Sedecimiella taiwanensis93
Sedecimiella taiwanensis96
Fonsecaea pedrosoi97
Paraconiothyrium cyclothyrioides
Xenoacremonium sinensis
Talaromyces trachyspermus
Zygoascus polysorbophila
Microascus intricatus
Myrmecridium sp.80
Keratinophyton turgidum81
Parafuscosporella mucosa87
Lulworthia sp.88
Candida pseudolambica89
Monocillium griseo-ochraceum90
Psathyrella subsingeri93
Coniochaeta boothii93.8
Arachnotheca glomerata94
Phlebiopsis cf.ravenelii95
Uncultured fungus95
Cymatoderma dendriticum96
Ascoidea corymbosa96
Westerdykella capitulum
Aspergillus terreus
Paecilomyces dactylethromorphus
Neurospora crassa
Pleurostoma richardsiae
Penicillium pinophilum
Paecilomyces tenuis
Trichoderma effusum
Yunnania penicillata
Galactomyces pseudocandidum
Scedosporium dehoogii
Geotrichum cucujoidarum
Debaryomyces singareniensis
Didymosphaeria enalia
Savoryella nypae
Pseudallescheria apiosperma
Phellinus umbrinellus
Fusarium verticillioides
Cladophialophora devriesii
Fusarium oxysporum
Parathyridaria ramulicola86
Massarina rubi93
Gliomastix murorum
Nigrograna rhizophorae93
Cheiromyces inflatus85
Pithomyces chartarum
Scedosporium aurantiacum
Cladophialophora immunda
Amorosia littoralis97.6
Pseudochaetosphaeronema pandanicola
Phialemonium dimorphosporum
Curvularia lunata
Parathyridaria percutanea
Acremonium antarcticum
Periconia macrospinosa
Sarcopodium vanillae
Aspergillus aculeatinus
Fusarium incarnatum
Cylindrocladiella lageniformis
Penicillium janthinellum
Nigrograna mackinnonii
Kluyveromyces aestuarii
Galactomyces reessii97
Myriodontium keratinophilum96
Pichia norvegensis
Camarographium sp.96
Halorosellinia xylocarpi
Pseudeurotium ovale
Geotrichum candidum
Cystobasidium minutum
Lulworthia atlantica89
Septoria sp.91
Pararoussoella quercina92
Paecilomyces tenuis93
Neopyrenochaeta annellidica93
Synnemellisia aurantia93.7
Pseudochaetosphaeronema ginkgonis95
Roussoella solani96
Westerdykella globosa
Eutypella scoparia
Hornodermoporus martius
0.3
0.2
0.1
0

Supplementary Fig. 2. The heatmap for the diversity of species from sediment samples SZ-1 and SZ-2 (color ranging represents relative abundance (%)). HTS, DPM, and FiChip indicate the results of high through-put sequencing, dilution-plate method, and *in situ* cultivation, respectively. Source data are provided as a Source data file.

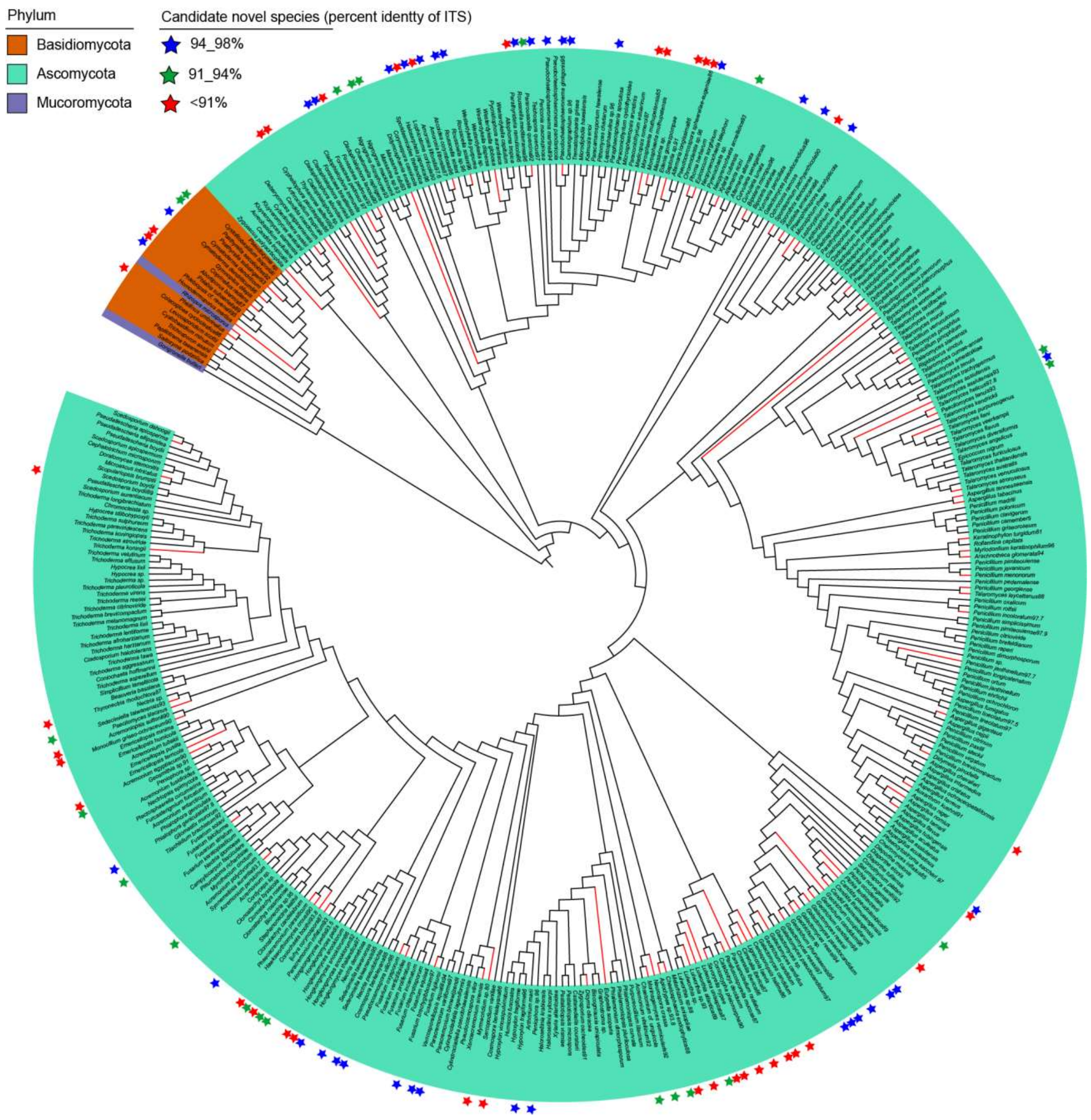

Supplementary Fig. 3. Phylogenetic tree of species isolated from 66 sediment samples. The phylogenetic tree was constructed from the full-length ITS gene sequences. Major phylum names are indicated and marked in different colors. Red branches on the trees indicate that the species was isolated only from new fungal culturomics techniques (FECM and FiChip). Candidate novel species are indicated by stars (blue stars indicate ITS sequence similarity between 94% and 98% comparing to the NCBI database, green stars indicate similarity between 91% and 94%, and red stars indicate similarity below 91%). In addition, detailed information of each species is shown in Additional file 2: Table S5.

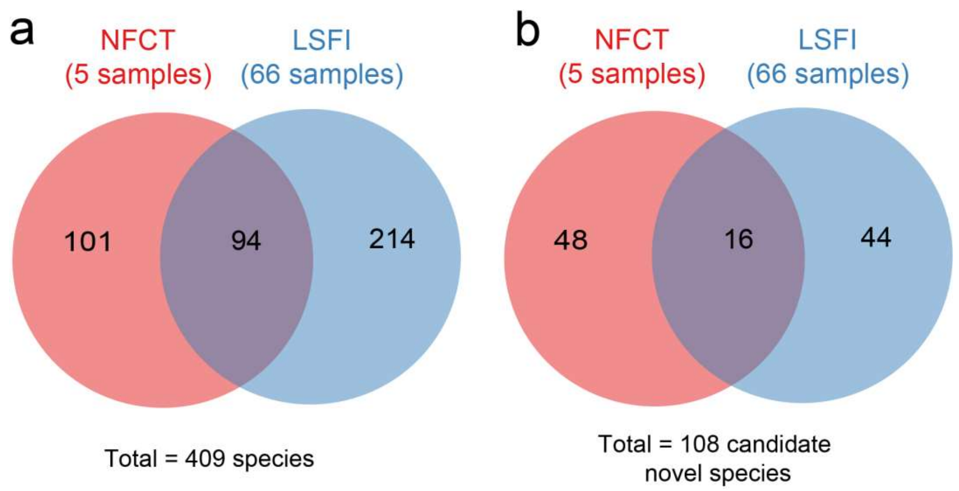

Supplementary Fig. 4. Venn diagram between new fungal culturomics techniques (NFCT) and large-scale fungal isolation (LSFI). (a). Comparison of species diversity between NFCT and LSFI. (b). Comparison of the number of potential novel species between NFCT and LSFI.

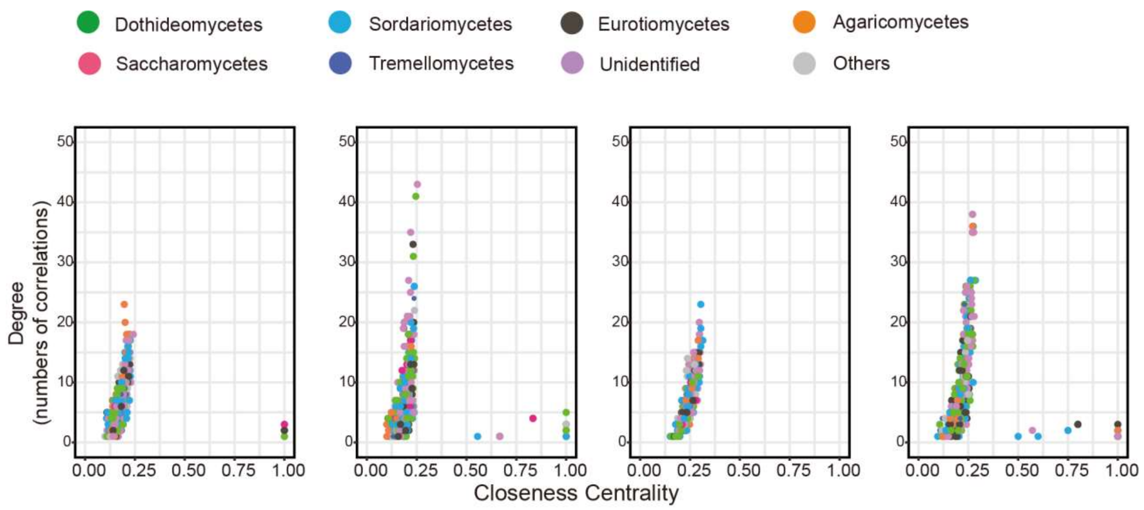

Supplementary Fig. 5. Comparison of node-level topological features in Fig. 5a (degree and closeness centrality) demonstrating the high degree and closeness centrality for the unidentified and other taxa at class level with the extension of enrichment period.

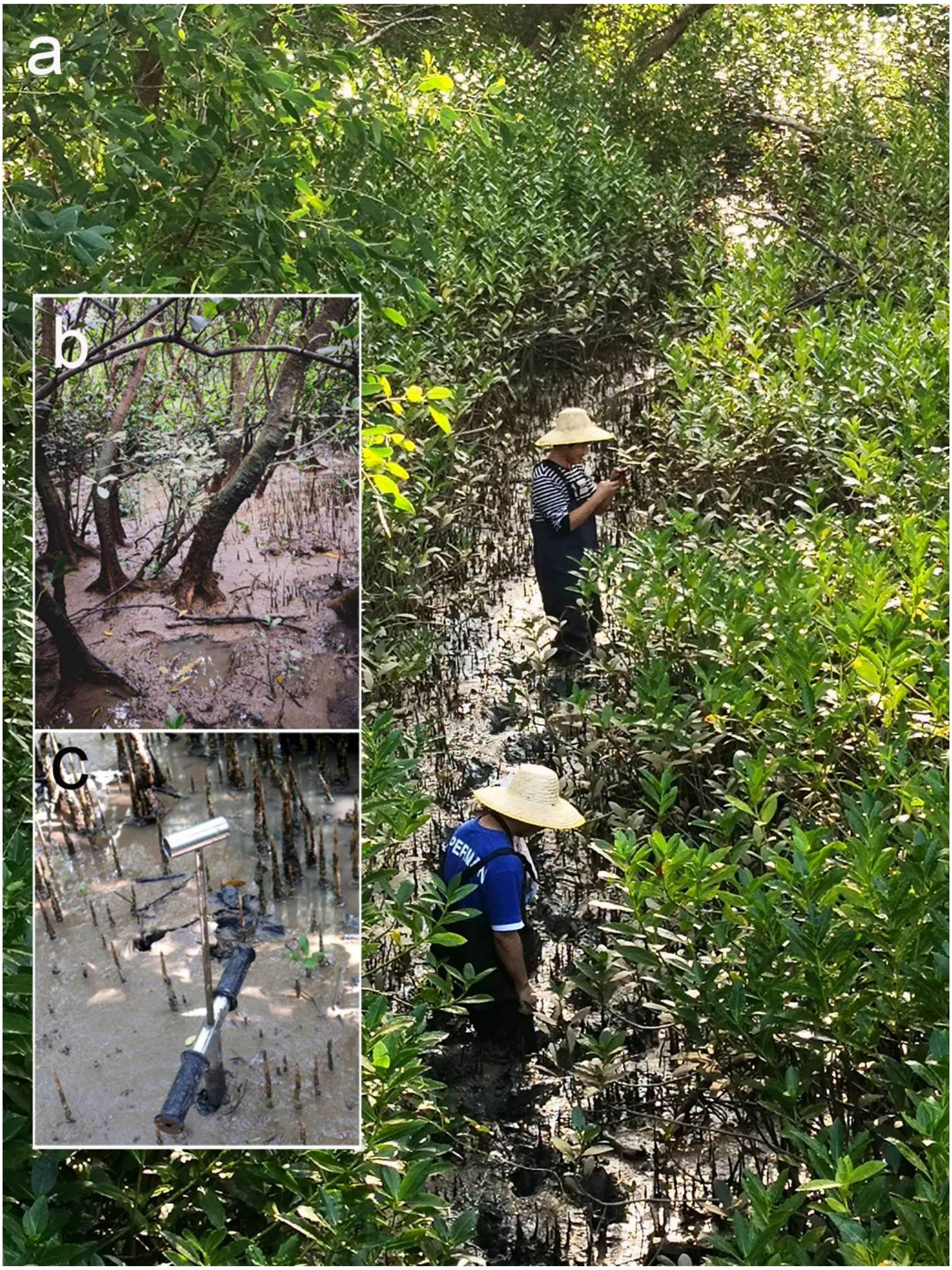

Supplementary Fig. 6 Scenes of visited mangrove forests in Guangdong Province, China. **a.** National Mangrove Nature Reserve of Zhanjiang. **b.** National Mangrove Nature Reserve of Futian Shenzhen. **c.** A surface sediment sampler.

Supplementary Table 6 Cultivable fungal numbers at different periods of enrichment cultivation

Sample	Enrichment-culture (days)	strains number	Species number
ZJ-1	0	66	30
ZJ-1	7	84	22
ZJ-1	14	72	29
ZJ-1	21	34	11
ZJ-2	0	77	34
ZJ-2	7	79	24
ZJ-2	14	46	21
ZJ-2	21	35	14
ZJ-3	0	73	28
ZJ-3	7	56	23
ZJ-3	14	36	11
ZJ-3	21	2	1

Supplementary Table 7 Primers and PCR programs used in this study

Locus	Primer	Sequence	PCR program
ITS	ITS1	5’-TCCGTAGGTGAACCTGCGG-3’	90 s initial denaturation at 94 °C, 35 cycles of 45 s at 94 °C, 15 s at 55 °C, and 45 s at 72 °C, with a final 10 min elongation at 72 °C.
	ITS4	5’-TCCTCCGCTTATTGATATGC-3’	
LSU	LR0R	5’-ACCCGCTGAACTTAAGC-3’	90 s initial denaturation at 94 °C, 35 cycles of 45 s at 94 °C, 15 s at 51 °C, and 45 s at 72 °C, with a final 10 min elongation at 72 °C.
	LR5	5’-TCCTGAGGGAAACTTCG-3’	
SSU	NS1	5’-GTAGTCATATGCTTGTCTC-3’	90 s initial denaturation at 94 °C, 35 cycles of 45 s at 94 °C, 15 s at 53 °C, and 45 s at 72 °C, with a final 10 min elongation at 72 °C.
	NS4	5’-CTTCCGTCAATTCCTTTAAG-3’	
rpb1	RPB1-F7	5'-CRACACAGAAGAGTTTGAAGG-3	90 s initial denaturation at 94 °C, 5 cycles of 94 °C 45 s, 58 °C 45 s, 72 °C 2 min; 5 cycles of 94 °C 45 s, 57 °C 45 s, 72 °C 2 min; 35 cycles of 45 s at 94 °C, 15 s at 56 °C, and 45 s at 72 °C, with a final 10 min elongation at 72 °C.
	RPB1-G2R	5'-GTCATYTGDGTDGCDGGYTCDCC-3'	
rpb2	RPB2-5F2	5'-GGGGWGAYCAGAAGAAGGC-3'	90 s initial denaturation at 94 °C, 35 cycles of 45 s at 94 °C, 15 s at 54 °C, and 45 s at 72 °C, with a final 10 min elongation at 72 °C.
	fRPB2-7cR	5'-CCCATRGCTTGYTTRCCCAT-3'	
tef1	983F	5'-GCYCCYGGHCAYCGTGAYTTYAT-3'	90 s initial denaturation at 94 °C, 35 cycles of 45 s at 94 °C, 15 s at 56 °C, and 45 s at 72 °C, with a final 10 min elongation at 72 °C.
	2218R	5'-ATGACACCRACRGCRACRGTYTG-3'	
tub2	Bt2a	5'-GGTAACCAAATCGGTGCTGCTTTC-3'	90 s initial denaturation at 94 °C, 35 cycles of 45 s at 94 °C, 15 s at 55 °C, and 45 s at 72 °C, with a final 10 min elongation at 72 °C.
	Bt2b	5'-ACCCTCAGTGTAGTGACCCTTGGC-3'	
cam	CF1	5'-GCCGACTCTTTGACYGARGAR-3'	90 s initial denaturation at 94 °C, 35 cycles of 45 s at 94 °C, 15 s at 55 °C, and 45 s at 72 °C, with a final 10 min elongation at 72 °C.
	CF4	5'-TTTYTGCATCATRAGYTGGAC-3'	

Supplementary Table 8 Strain and sequence accession numbers of new species

Species name	Srain No.	Type	Isolation method	Genbank accession numbers							
				ITS	LSU	SSU	*rpb2*	*tef1*	*rpb1*	*tub2*	*cam*
Apinisia pyriforme	CGMCC 3.22427	Holotype	FiChips	OQ798931	OQ758128	-	-	-	-	-	-
	CGMCC 3.22430		FiChips	OQ798927	OQ758124	-	-	-	-	-	-
	CGMCC 3.22431		FiChips	OQ798928	OQ758125	-	-	-	-	-	-
	CGMCC 3.22437		FiChips	OQ798929	OQ758126	-	-	-	-	-	-
	CGMCC 3.22438		FiChips	OQ798930	OQ758127	-	-	-	-	-	-
Arachnotheca pulvereum	CGMCC 3.22341	Holotype	FiChips	OQ798932	OQ758129	-	-	-	-	-	-
	CGMCC 3.22347		FiChips	OQ798933	OQ758130	-	-	-	-	-	-
Auxarthron pyriforme	CGMCC 3.22381	Holotype	FiChips	OQ798935	OQ758132	-	-	-	-	-	-
	CGMCC 3.22382		FiChips	OQ798934	OQ758131	-	-	-	-	-	-
Coniochaeta aurantiaca	CGMCC 3.22339	Holotype	FiChips	OQ798936	OQ758133	-	-	-	-	-	-
	CGMCC 3.22340		FiChips	OQ798937	OQ758134	-	-	-	-	-	-
Fusarium aseptatum	CGMCC 3.22496	Holotype	FECM	OQ798938	-	-	OQ808997	OQ809037	OQ756141	-	OQ808984
	CGMCC 3.22511		FECM	OQ798939	-	-	OQ808998	OQ809038	OQ756142	-	OQ808985
Heteroacremonium album	CGMCC 3.22389		FiChips	OQ798940	OQ758135	OQ758172	OQ808999	OQ809039	-	-	-
	CGMCC 3.22392		FiChips	OQ798941	OQ758136	OQ758173	OQ809000	OQ809040	-	-	-
	CGMCC 3.22405	Holotype	FiChips	OQ798942	OQ758137	OQ758174	OQ809001	OQ809041	-	-	-
	CGMCC 3.22409		FiChips	OQ798943	OQ758138	OQ758175	OQ809002	OQ809042	-	-	-
Heteroacremonium rugosum	CGMCC 3.22520	Holotype	FECM	OQ798944	OQ758139	OQ758176	OQ809003	OQ809043	-	-	-
	CGMCC 3.22522		FECM	OQ798945	OQ758140	OQ758177	OQ809004	OQ809044	-	-	-
Neomorinagamyces pyriformis	CGMCC 3.22317	Holotype	FiChips	OQ798946	OQ758141	-	OQ809005	-	-	OQ808971	-
	CGMCC 3.22326		FiChips	OQ798947	OQ758142	-	OQ809006	-	-	OQ808972	-

Neoroussoella sedimenticola	CGMCC 3.22468	Holotype	FiChips	OQ798948	OQ758143	-	OQ809007	OQ809045	-	-	-
	CGMCC 3.22470		FiChips	OQ798949	OQ758144	-	OQ809008	OQ809046	-	-	-
Nothoacremoniopsis irregularis	CGMCC 3.22386	Holotype	FiChips	OQ798951	OQ758146	OQ758179	OQ809010	OQ809048	-	-	-
	CGMCC 3.22387		FiChips	OQ798950	OQ758145	OQ758178	OQ809009	OQ809047	-	-	-
	CGMCC 3.22388		FiChips	OQ798952	OQ758147	OQ758180	OQ809011	OQ809049	-	-	-
Nothoacremoniopsis sedimenticola	CGMCC 3.22383	Holotype	FiChips	OQ798953	OQ758148	OQ758181	OQ809012	OQ809050	-	-	-
	CGMCC 3.22385		FiChips	OQ798954	OQ758149	OQ758182	OQ809013	OQ809051	-	-	-
Nothoroussoella irregularis	CGMCC 3.22466	Holotype	FiChips	OQ798955	OQ758150	OQ758183	OQ809014	OQ809052	-	-	-
	CGMCC 3.22467		FiChips	OQ798956	OQ758151	OQ758184	OQ809015	OQ809053	-	-	-
Okeanomyces guttulatus	CGMCC 3.22358	Holotype	FiChips	OQ798957	OQ758152	OQ758185	-	-	-	-	-
	CGMCC 3.22360		FiChips	OQ798958	OQ758153	OQ758186	-	-	-	-	-
	CGMCC 3.22371		FiChips	OQ798959	OQ758154	OQ758187	-	-	-	-	-
	CGMCC 3.22373		FiChips	OQ798960	OQ758155	OQ758188	-	-	-	-	-
Paramoleospora guttulata	CGMCC 3.22494	Holotype	FECM	OQ798961	OQ758156	OQ758189	-	-	-	-	-
	CGMCC 3.22495		FECM	OQ798962	OQ758157	OQ758190	-	-	-	-	-
Penicillium limoniforme	CGMCC 3.22512	Holotype	FECM	OQ798963	-	-	OQ809016	-	-	OQ808973	OQ808986
	CGMCC 3.22517		FECM	OQ798964	-	-	OQ809017	-	-	OQ808974	OQ808987
Penicillium sedimenticola	CGMCC 3.22527	Holotype	FECM	OQ798965	-	-	OQ809018	-	-	OQ808975	OQ808988
	CGMCC 3.22528		FECM	OQ798966	-	-	OQ809019	-	-	OQ808976	OQ808989
Phaeocollarina guttulata	CGMCC 3.22396	Holotype	FiChips	OQ798968	OQ758159	OQ758192	OQ809021	OQ809055	-	-	-
	CGMCC 3.22401		FiChips	OQ798967	OQ758158	OQ758191	OQ809020	OQ809054	-	-	-
Pseudocastanedospora	CGMCC 3.22534	Holotype	FECM	OQ798969	OQ758160	-	-	-	-	-	-

guangdongensis											
	CGMCC 3.22540		FECM	OQ798970	OQ758161	-	-	-	-	-	-
Pseudochaetosphaeronema irregulare	CGMCC 3.22458	Holotype	FiChips	OQ798972	OQ758163	OQ758194	OQ809023	OQ809057	-	-	-
	CGMCC 3.22461		FiChips	OQ798971	OQ758162	OQ758193	OQ809022	OQ809056	-	-	-
Pseudosynnemellisia favida	CGMCC 3.22477	Holotype	FiChips	OQ798973	OQ758164	OQ758195	OQ809024	OQ809058	-	-	-
	CGMCC 3.22483		FiChips	OQ798974	OQ758165	OQ758196	OQ809025	OQ809059	-	-	-
Sedecimiella alba	CGMCC 3.22402	Holotype	FiChips	OQ798975	OQ758166	OQ758197	OQ809026	OQ809060	-	-	-
	CGMCC 3.22404		FiChips	OQ798976	OQ758167	OQ758198	OQ809027	OQ809061	-	-	-
Sedecimiella funiculosus	CGMCC 3.22348	Holotype	FiChips	OQ798977	OQ758168	OQ758199	OQ809028	OQ809062	-	-	-
	CGMCC 3.22356		FiChips	OQ798978	OQ758169	OQ758200	OQ809029	OQ809063	-	-	-
Sedecimiella subulata	CGMCC 3.22330	Holotype	FiChips	OQ798979	OQ758170	OQ758201	OQ809030	OQ809064	-	-	-
	CGMCC 3.22332		FiChips	OQ798980	OQ758171	OQ758202	OQ809031	OQ809065	-	-	-
Talaromyces ellipsoideus	CGMCC 3.22439	Holotype	FiChips	OQ798985	-	-	OQ809036	-	-	OQ808981	OQ808994
	CGMCC 3.22442		FiChips	OQ798981	-	-	OQ809032	-	-	OQ808977	OQ808990
	CGMCC 3.22447		FiChips	OQ798982	-	-	OQ809033	-	-	OQ808978	OQ808991
	CGMCC 3.22456		FiChips	OQ798983	-	-	OQ809034	-	-	OQ808979	OQ808992
	CGMCC 3.22457		FiChips	OQ798984	-	-	OQ809035	-	-	OQ808980	OQ808993
Talaromyces phialiformis	CGMCC 3.22415	Holotype	FiChips	OQ798986	-	-	-	-	-	OQ808982	OQ808995
	CGMCC 3.22417		FiChips	OQ798987	-	-	-	-	-	OQ808983	OQ808996
